# Nitrite Oxidation during Ozonation Revisited: Mechanisms of Nitration Reactions

**DOI:** 10.1021/acs.est.6c02098

**Published:** 2026-06-18

**Authors:** Tarek Manasfi, Christoph Dieziger, Simon A. Rath, Daisuke Minakata, Urs von Gunten

**Affiliations:** † Swiss Federal Institute of Aquatic Science and Technology (Eawag), 8600 Dübendorf, Switzerland; ‡ Department of Civil, Environmental, and Geospatial Engineering, 3968 Michigan Technological University, Houghton, Michigan 49931, United States; § School of Architecture, Civil and Environmental Engineering (ENAC), Ecole Polytechnique Fédérale de Lausanne (EPFL), 1015 Lausanne, Switzerland

**Keywords:** hydroxyl radical, peroxynitrite, nitrogen dioxide, γ-radiolysis, ozone, nitrite, nitration, nitro compounds, diuron, nitrodiuron, carbendazim, nitrocarbendazim, municipal wastewater effluent, reactive nitrogen species

## Abstract

The formation of toxicologically relevant nitro compounds has been observed during ozonation of nitrite-containing secondary wastewater effluents, but their formation mechanism remains unknown. To identify key nitrating species, three reaction systems were investigated: ozonation of nitrite (O_3_/NO_2_^–^), peroxynitrite (ONOOH/ONOO^–^), and hydroxyl radical oxidation of nitrite with γ-radiolysis (γ/NO_2_^–^). Nitrite ozonation (O_3_/NO_2_^–^) yielded significant amounts of the nitrating agent nitrogen dioxide ^•^NO_2_ (9.4% at pH 7 to 22% at pH 12) besides the main product nitrate. No peroxynitrite formation was detected during ozonation of nitrite-containing waters, suggesting that ^•^NO_2_ is the key nitrating species. A comparison of nitro compound formation from 20 aromatic compounds in the three reaction systems showed a consistent formation of nitro compounds with minor differences. Furthermore, the pH-dependence patterns of nitration for two micropollutants (diuron and carbendazim) revealed similarities between the O_3_/NO_2_^–^ and γ-radiolysis (γ/NO_2_^–^) systems, unlike peroxynitrite. These trends, combined with the lack of detection of peroxynitrite during ozonation, a fast reaction of peroxynitrite with ozone (*k*_pH=9_ = (4.96 ± 0.40) × 10^6^ M^–1^s^–1^), suggest that a significant contribution of peroxynitrite is highly unlikely. Overall, nitration during ozonation involves nitrite oxidation to ^•^NO_2_, mostly by hydroxyl radical and to a minor extent by ozone.

## Graphical abstract

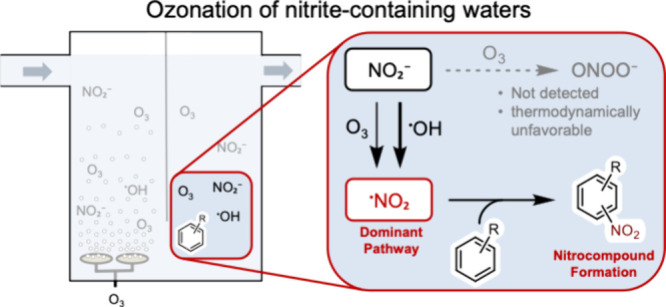

## 1 Introduction

Discharge of organic micropollutants from industrial processes, domestic use, and agriculture puts freshwater ecosystems and water resources for drinking water production at risk.^1^ Even though these chemicals are present at relatively low concentrations (ranging from ng/L to μg/L), they can still induce adverse impacts to human health and the ecosystem.^2^ One of the main pathways by which micropollutants enter aquatic systems is their discharge from municipal wastewater treatment plants (WWTPs).^3–6^ In conventional WWTPs, many micropollutants are not effectively removed by the typically applied physicochemical and biological processes.^7,8^ Among other processes (e.g., activated carbon), the addition of an ozonation step followed by biological postfiltration has been identified as an effective upgrade that mitigates micropollutants in municipal wastewater effluents.^9–13^ Ozone acts as a potent and selective oxidant that, along with hydroxyl radicals formed during its decomposition, can abate a wide variety of organic micropollutants.^14–16^ However, ozonation typically does not lead to the mineralization of micropollutants but rather to the formation of transformation products (TPs).^17–20^ Mostly, these TPs lose the original bioactivity of the target compounds, which is the desired outcome of such treatment.^20^ Nevertheless, during ozonation in the presence of water matrix components (e.g., natural organic matter, bromide), undesired and (potentially) harmful disinfection byproducts (DBPs) such as aldehydes, ketones, and bromate are formed.^21–24^

An increase in mutagenicity has been observed sometimes during ozonation of municipal wastewaters and a biological post-treatment often led to its abatement.^25–27^ A recent study by Manasfi et al.^28^ highlighted an increase in mutagenicity after ozonation of secondary municipal wastewater effluent in the presence of nitrite even at relatively low concentrations (≥50 μg_N_/L). Good wastewater treatment practice includes a nitrification process to reduce nitrite concentrations in secondary effluents; however, sometimes high nitrite concentrations remain, due to incomplete or missing nitrification related to seasonal effects.^29^ It was hypothesized that the increased mutagenicity during wastewater ozonation in the presence of nitrite is attributed to the formation of nitro (aromatic) compounds which have been demonstrated to be formed during oxidative water treatment in a previous study.^30^ During ozonation, nitro compounds can be formed from primary or secondary amines, but so far, there is limited evidence for their formation from non-nitrogenous compounds.^15,31–33^ However, the specific reactive nitrogen species (RNS) responsible and the dominant formation pathways during ozonation remain unclear, particularly for non-nitrogenous precursors.^34^ Many nitro compounds, in particular nitroaromatics, are known to be mutagenic, genotoxic, and/or carcinogenic.^35–38^ Therefore, the formation of this class of compounds during ozonation is of concern in advanced wastewater treatment. Elucidating the formation pathways of nitro compounds during ozonation of nitrite-containing waters is a crucial step toward the development of mitigation strategies.

Ozone reacts with nitrite rapidly and quantitatively to nitrate (*k*_O_3__ = 3.7 × 10^5^ M^–1^ s^–1^).^39^ However, as side reactions or en route to nitrate, other nitrogenous intermediates such as peroxynitrite and nitrogen dioxide radical (^•^NO_2_) have been postulated, both known for their nitrating capabilities.^40–43^

Previously, nitrophenols were detected during ozonation of solutions containing nitrite and phenols in synthetic waters.^30,44^ Furthermore, isomers of nitroaniline were detected upon ozonation of solutions containing aniline and nitrite in the pH range 7–10.^41^ Two potential mechanisms for nitration of aniline were proposed during ozonation of nitrite-containing solutions.^41^ The first proposed mechanism at neutral pH involves peroxynitrous acid, formed from the reaction of hydrogen peroxide and nitrite. Peroxynitrous acid is highly unstable and dissociates to a hydroxyl radical (^•^OH) and ^•^NO_2_.^41,45^ The second proposed pathway at alkaline pH involves ^•^OH and ^•^NO_2_, which is formed by the reaction of ^•^OH with nitrite.^41^ In another study, the formation of nitroaniline was demonstrated in aniline- and nitrite-containing waters for hydroxyl radical- and sulfate radical-based processes, which supports the previously proposed reaction pathways.^46^

The formation of peroxynitrite during ozonation of nitrite-containing solutions was reported at an alkaline pH with a yield of about 2%.^40^ However, little is known about the behavior of peroxynitrite under conditions relevant for water and wastewater treatment and about its role in the formation of nitro compounds during ozonation in the presence of nitrite.

This study aims to elucidate the main reactive species responsible for the nitration of organic compounds during ozonation of nitrite-containing waters. Two main RNS, peroxynitrous acid/peroxynitrite (ONOOH/ONOO^–^) (here referred to collectively as peroxynitrite) and ^•^NO_2_, hypothesized to be involved in inducing nitration reactions, were examined. Specifically, the following aspects were investigated: (i) formation of peroxynitrite from nitrite during ozonation and its decay under various water quality conditions (e.g., pH, presence of carbonate, nitrite concentrations), (ii) formation of ^•^OH during ozonation with an ensuing formation of ^•^NO_2_ from nitrite oxidation, (iii) formation of nitro compounds from selected model compounds, and (iv) effect of pH on the formation of nitro compounds from two selected model compounds (diuron and carbendazim) in the three reaction systems (ozone/nitrite, peroxynitrite, and ^•^NO_2_).

## 2 Materials and Methods

### 2.1 Reagents, Chemicals, and Stock Solutions

A summary of reagents and chemicals used in this study is provided in Table S1 (Supporting Information, SI). For the nitration product nitrodiuron, a reference standard was synthesized in-house, confirmed by NMR, and used for quantification (see Sections S1.1 and S10). For other nitrated compounds, product formation was assessed based on the peak area. The preparation of stock solutions is described in Section S1.1.

### 2.2 Assessment of Peroxynitrite Reactivity under Water Treatment Conditions

The influence of multiple factors including pH, the presence of dissolved CO_2_, ionic strength, and nitrite concentration on the stability of peroxynitrite was studied. Peroxynitrite concentrations were determined by spectrophotometry at 302 nm (ε = 1705 M^–1^cm^–1^).^47^ Under conditions of fast peroxynitrite decay, the rapid decrease of absorbance was measured continuously until the absorbance reached a stable value. Under conditions for which peroxynitrite was stable (half-life times of hours), the absorbance was measured at various time points until the absorbance decreased by 50%. Apparent first-order rate constants (*k*_obs_, s^–1^) were derived by plotting the natural logarithm of the relative absorbance as a function of time. These first-order rate constants, averaged from triplicates, were used to calculate the half-life time of peroxynitrite. Details for these experiments are presented in Section S2.

### 2.3 Sample Collection and Preparation

#### 2.3.1 Wastewater Samples

To assess the formation of nitro compounds under realistic conditions, a secondary wastewater effluent sample was collected. Water quality parameters of the Swiss wastewater are summarized in Table S3. Secondary wastewater effluent was filtered (0.45 μm, Pall Corporation, South Wagner Road, USA), spiked with a model compound (diuron or carbendazim at 1 μM) and nitrite (0.03, 0.1, and 0.5 mg_N_/L which corresponds to 2.1, 0.7, and 3.5 μM, respectively) and ozonated at specific ozone doses of 0.1, 0.5, and 1 gO_3_/g DOC.

#### 2.3.2 Formation of Nitro Compounds from Model Compounds in Three Reaction Systems

The selected model compounds (Table S2) including phenol-type compounds and pharmaceuticals, pesticides, and industrial chemicals with various functional groups were tested for nitro product formation in three reaction systems: (i) ozonation of nitrite-containing solutions, (ii) peroxynitrite-spiked solutions, and (iii) γ-radiolysis (^•^OH generated in nitrite-containing solutions). Oxidation of nitrite by ^•^OH represents the main proposed pathway during ozonation. Details of sample preparation are provided in Section S1.2.2.

In brief, aliquots of stock solutions of the selected model compounds were added to 10 mL glass vials to achieve a final concentration of 10 μM for most compounds (Table S2) in phosphate buffer pH 8 (10 mM in ozonation and γ-radiolysis experiments or 50 mM in peroxynitrite experiments). Nitrite and ^•^OH scavengers (tertiary butanol (*t*-BuOH) or dimethyl sulfoxide (DMSO)) were then added to the solutions. Even though no second-order rate constants for the reaction of ^•^NO_2_ with *t*-BuOH have been determined, aliphatic compounds have very low reactivity with ^•^NO_2_.^48^ The scavenging of peroxynitrite with DMSO is negligible, since the nitrodiuron formation is the same in the absence and presence of DMSO (Figure S14). The resulting solutions were treated further by adding aliquots of an ozone or a peroxynitrite stock solution (Section S1.1).

For γ-radiolysis experiments, solutions containing model compounds were sparged with N_2_O for around 20 min to reach saturation. Aliquots of the N_2_O-saturated solutions were added to LC autosampler vials to which an air-saturated nitrite solution and oxygen-saturated ultrapure water were added. The mixture had a percentage saturation of approximately 85:15% N_2_O/O_2_ (Section S1.2.2).

##### 2.3.2.1 Effect of pH and the Presence of the ^•^OH Scavenger on Nitro Products Formation from Diuron and Carbendazim

Diuron and carbendazim were used for an in-depth evaluation of the effects of pH and the presence of DMSO or *t*-BuOH on the formation of nitro products in the three reaction systems. Details for the preparation of these solutions are provided in Section S1.2.2.

Samples were analyzed using HPLC-UV and/or HPLC-high-resolution mass spectrometry (HRMS) to monitor the decrease of the parent compound and the formation of nitro products. The used analytical methods are described in Section S1.3.

#### 2.3.3 γ-Radiolysis and Production of ^•^NO_2_

γ-Radiolysis was performed using a ^60^Co γ-radiation source (Gammacell 220, Atomic Energy of Canada, Ltd.). The dose rate was determined by dosimetry in a formate-containing solution and was estimated at approximately 0.13 kGy/h.^83^ ^•^NO_2_ was formed by the reaction of nitrite with ^•^OH in the presence of N_2_O, according to eqs 1–3:

H2O→γeaq−+H++OH• 1

eaq−+N2O+H2O→OH•+N2+OH− 2

OH•+NO2−→OH−+NO2• 3

Further details regarding sample preparation are provided in Section S1.2.2.

### 2.4 Determination of ^•^OH Yield during Nitrite Ozonation and from Peroxynitrite Decay

The yield of ^•^OH produced from the ozone-nitrite reactions and from peroxynitrite was determined by quantifying formaldehyde formation in the presence of *t*-BuOH using the Hantzsch reagent.^49^ Details for this method are described in Section S4.1.

### 2.5 Determination of Second-Order Rate Constants *k*_O_3__

The second-order rate constants *k*_O_3__ for the reaction of carbendazim with ozone were determined by competition kinetics using cinnamic acid as a reference compound.,^50,51^ The formation of benzaldehyde, used as a proxy for cinnamic acid consumption, was quantified by HPLC-UV.^100^ Details for this method are described in Section S8.

### 2.6 Quantum Chemical Calculations

Gaseous-phase free energies of reaction and activation at a standard temperature of 298 K were determined by computing the wave function for given molecular and radical structures at the level of unrestricted correlation functional and basis set, B3LYP/6-311+G** theory.^52^ This exchange-correlation functional has been tested and used by a previous study investigating the reaction of O_3_ with nitrite.^40^ The aqueous-phase free energy of solvation was calculated using the universal solvation model (SMD) to determine the aqueous-phase free energies of reaction and activation of each elementary reaction.^53^ To correct the electronic energy resulting from the spin contamination of singlet, triplet, and quintet states, an approximate spin-projection method was used.^54,55^ All of the computations were performed using Gaussian 16 revision C.01.^56^

## 3 Results and Discussion

### 3.1 Potential of Peroxynitrite as a Nitrating Agent

#### 3.1.1 Peroxynitrite Stability in Aqueous Solution

To assess peroxynitrite formation during ozonation, first, the dependence of the stability of peroxynitrite on various water parameters (without ozone) was tested. Therefore, peroxynitrite from a commercially available stock (Table S1) was dosed to reach 0.05–0.5 mM in aqueous solutions with varying ionic strengths, pH, CO_2_, bicarbonate, and nitrite concentrations.

Peroxynitrite stability is strongly pH-dependent, with a very short half-life time (*t*_1/2_) of a few seconds at pH 7 (∼4 s at pH 7.2) and not detectable in cuvette experiments at pH 6.2 (Table S4). In contrast, peroxynitrite was very stable at high pH (*t*_1/2_ ∼ 3 h at pH 11.3, Table S4). The peroxynitrite stability was affected by the bicarbonate concentration with a decrease of *t*_1/2_ by a factor of 4 (from 16.3 to 4 s) when the bicarbonate concentration increased from 0 to 5 mM at pH 8.2 (Table S6). The carbonate effect in this study is comparable to a previous investigation.^57^ In contrast, ionic strength variation had no effect on peroxynitrite stability (Figure S4). Nitrite, which is present in excess when the peroxynitrite formation during ozonation is investigated, had a minor effect on peroxynitrite stability (Section S2.4). While no effect was detected at pH 7, the first-order rate constants for peroxynitrite decomposition increased ∼20% at pH 8 and ∼25% at pH 9, respectively, for the highest tested nitrite concentration (40-fold molar excess relative to peroxynitrite) (Figure S5). These findings are in agreement with a previous study in which very high excess of nitrite had only a marginal effect on peroxynitrite stability at pH 5.2.^58^ Overall, the pH plays the most important role for peroxynitrite stability in aqueous solutions, which was taken into account for the peroxynitrite formation tests during ozonation of nitrite-containing solutions.

#### 3.1.2 Peroxynitrite Formation during Ozonation of Nitrite

A previous study found that peroxynitrite is formed at a yield of 2.6% during ozonation of nitrite-containing waters (2.5 mM) at pH 11, and peroxynitrite was determined spectrophotometrically (302 nm).^40^ In the present study, peroxynitrite formation during ozonation of nitrite-containing waters was investigated at pH 9 and 11 in the absence and presence of ^•^OH scavengers (*t*-BuOH, DMSO) (Figures S6 and S7). However, the previous results could not be replicated,^40^ and peroxynitrite was not detected under any of the tested experimental conditions with a detection limit of 1 μM (Table S4 and Section S3.1 for details). Therefore, peroxynitrite formation during ozonation seems unlikely. In addition, the second-order rate constant of the ozone reaction with peroxynitrite at pH 9 (*k*_O_3__ = (4.96 ± 0.40) × 10^6^ M^–1^ s^–1^) determined using competition kinetics, as described in Section S3.2, suggests that a small yield of peroxynitrite would get quickly oxidized by ozone depending on the nitrite concentration and the ozone dose. Based on these findings, a significant contribution from peroxynitrite to the nitration of organic compounds during ozonation seems unlikely.

### 3.2 Potential of Nitrogen Dioxide as a Nitrating Agent during Ozonation: ^•^OH Formation from the Ozone-Nitrite Reaction and Yield of Nitrogen Dioxide

The formation of ^•^OH during ozonation of nitrite was tested to investigate the potential extent of ^•^NO_2_ formation from this reaction (eq 4). Figure 1 shows the ^•^OH yields (mol ^•^OH formed/mol O_3_ consumed) as a function of pH for the reaction of ozone with nitrite in molar excess of nitrite (for data, see Tables S8 and S9). For the experimental conditions described in Figure 1, the ozone consumption is fully controlled by nitrite even at pH 12, with a half-life time of about 4 ms,^59^ and ^•^OH was entirely quenched by *t-*BuOH and its yield increased from 9.4% at pH 7 to 22% at pH 12. The observed ^•^OH yields form a sigmoidal curve, suggesting a pH-dependence with an apparent p*K*_a_ of around 9.5, even though none of the added species in the solution has a p*K*_a_ in this order. Currently, the observed pH-dependence of the ^•^OH yield lacks a clear interpretation, and more detailed studies would be necessary to explore this behavior. Peroxynitrous acid, with a p*K*_a_ of 6.8, can undergo a peroxo bond homolysis producing ^•^OH and ^•^NO_2_ radicals.^60^ There has been no consensus regarding the yield of ^•^OH from peroxynitrite with significantly diverging yields reported previously.^61–63^ These divergences have been attributed to interferences from CO_2_ in earlier measurements because of the formation of an unstable adduct, ONOOC­(O)­O, decomposing rapidly to ^•^NO_2_ and carbonate (CO_3_^•–^) radicals.^64^ It should be noted that peroxynitrite as a nucleophile is known to react with carbonyls, although the rate is significantly lower than the reaction with CO_2_.^65^ This may influence the determined ^•^OH yields of some studies, when formaldehyde was used for the determination. When CO_2_ has been accounted for, ^•^OH yields between 10 and 30% have been mostly reported.^65–68^ In the present study, ^•^OH yields in the order of about 5–7% were measured from peroxynitrite decay at pH 7 and 8 (Figure S9). This suggests that, even if a minor peroxynitrite formation (≤2.5%) during ozonation is assumed, the contribution of peroxynitrous acid decay to the measured ^•^OH yield is minimal. At higher pH, the ^•^OH yield from peroxynitrous acid decomposition decreases, since peroxynitrite is more stable and does not yield ^•^OH.^69^ Therefore, the measured ^•^OH formation is dominated by the reaction of ozone with nitrite under these conditions.

**Figure 1:**
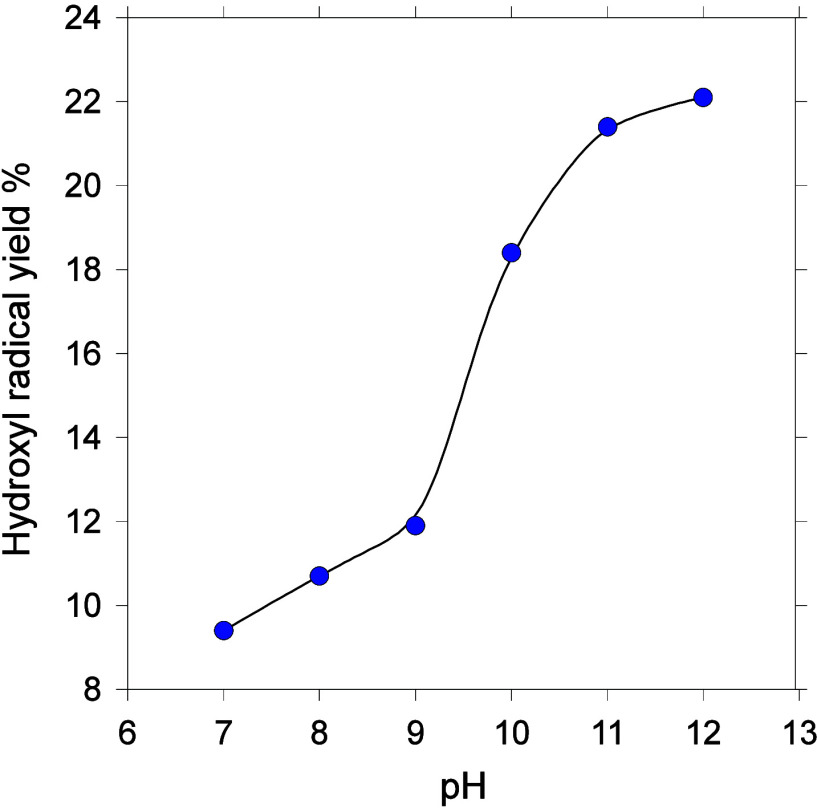
Ozonation of nitrite-containing solutions: Measured yields of ^•^OH (mol ^•^OH formed/mol O_3_ consumed) as a function of pH. Experimental conditions: Solutions containing nitrite (390 μM) and *t*-BuOH (200 mM) in phosphate buffer (10 mM) at pH 7–12 and ozone doses up to ≈350 μM. The yield of ^•^OH (Table S9) was obtained by performing linear regression of the formed formaldehyde at different ozone doses (Table S8). The ^•^OH yield was estimated to be twice the measured formaldehyde yield.^49^ The line is shown to guide the eye.

The formation of ^•^OH during ozonation of nitrite occurs through an electron-transfer reaction (eq 4),^40^ which is a minor but not negligible pathway compared to direct nitrate formation by an oxygen-transfer reaction (eq 5).^70^ Nitrite reacts with ozone to nitrogen dioxide and ozonide radicals (eq 4), which forms ^•^OH (eqs 6 and 7)^16^

NO2−+O3→NO2•+O3•− 4

$${{\mathrm{NO}}_{2}}^{-}+O_{3}\to {{\mathrm{NO}}_{3}}^{-}+O_{2}$$ 5

$${O_{3}}^{•-}⇋O^{•-}+O_{2}$$ 6

O•−+H2O⇋OH•+OH− 7

The significant formation of ^•^OH during ozonation of nitrite is an indirect evidence for nitrogen dioxide formation, which highlights its potential role in nitration reactions.

### 3.3 Quantum Chemical Calculations for the Ozone-Nitrite Reaction

To complement the experimentally observed three pathways of O_3_ with NO_2_^–^, quantum chemical computations were performed to investigate the gaseous- and aqueous-phase elementary reactions undergoing oxygen-atom and single-electron transfers.

Distinctive different underlying reaction mechanisms were observed between gaseous- and aqueous phases. In the gaseous phase, quantum chemical computations suggest that both the oxygen atom-transfer reaction to form nitrate (eq 5, blue pathway in Figure 2a) and the reaction of ozone with nitrite forming peroxynitrite (NO_2_^–^ + O_3_ → ONOO^–^ + ^1^O_2_, red pathway in Figure 2a) are multistep processes. Each reaction occurs via formation of a pre- and a postreaction adduct. Overall, the formation of nitrate is endergonic, and transition state 1 (TS1) is the only kinetic barrier (blue pathway). In the red pathway, the formation of peroxynitrite is slightly exergonic (i.e., −0.7 kcal/mol of gaseous-phase free energy of reaction, $\text{Δ}G_{\mathrm{gas},\mathrm{calc}}^{\mathrm{react}}$ ). However, the oxygen transfer from a stable postadduct of the first elementary reaction through TS2–2 to the final reaction product peroxynitrite (O_2_N–OOO^–^ → ONOO^–^ + ^1^O_2_) appears kinetically inhibitive with 29.6 kcal/mol of free energy of activation, $\text{Δ}G_{\mathrm{gas},\mathrm{calc}}^{\mathrm{act}}$ . In conclusion, the oxygen atom-transfer (eq 5, blue pathway) is the dominant reaction pathway compared to peroxynitrite formation in the gaseous phase.

**Figure 2:**
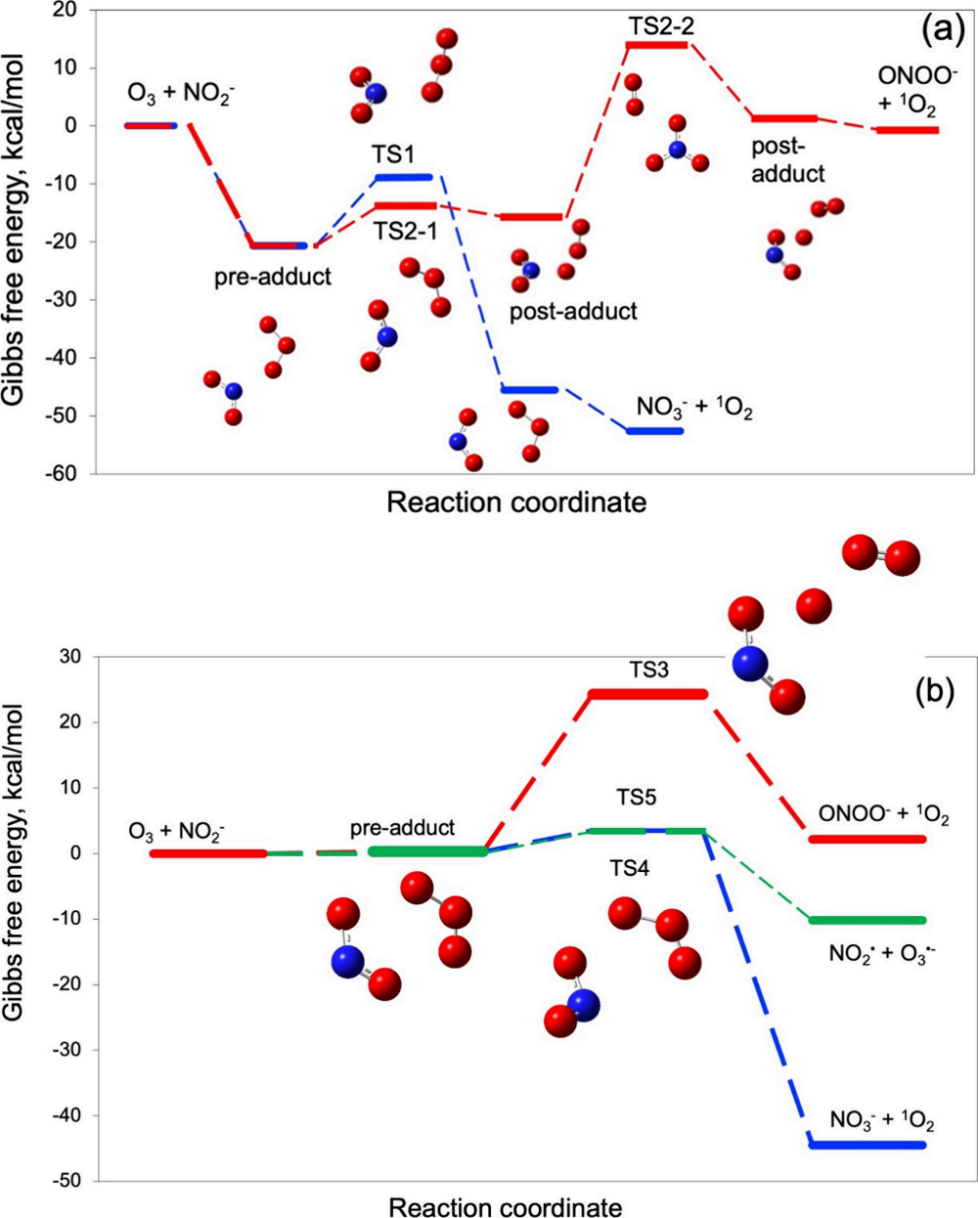
Gibbs free energy profiles at 298 K for the reaction of ozone with nitrite in (a) gaseous (oxygen atom-transfer pathway in blue and peroxynitrite formation pathway in red) and (b) aqueous phase (peroxynitrite formation pathway in red, oxygen atom-transfer pathway in blue, and single electron-transfer pathway in green). TS: transition state. Gibbs free energies were calculated at the level of unrestricted correlation functional and basis set of B3LYP/6-311+G**. A SMD solvation model was used for the aqueous phase energies.

Aqueous-phase computations also showed the formation of a preadduct (ONO–OOO^–^), though all three reactions were observed as single step (Figure 2b). A reaction forming peroxynitrite is endergonic with a slightly positive aqueous-phase free energy of reaction, $\text{Δ}G_{\mathrm{aq},\mathrm{calc}}^{\mathrm{react}}$ , of 0.3 kcal/mol (NO_2_^–^ + O_3_ → ONOO^–^ + ^1^O_2_, red pathway in Figure 2b). This reaction requires a significantly larger free energy of activation, $\text{Δ}G_{\mathrm{aq},\mathrm{calc}}^{\mathrm{act}}$ , value of 24.3 kcal/mol for TS3. In comparison, the reaction forming nitrate through oxygen atom-transfer is endergonic and requires only 3.5 kcal/mol of $\text{Δ}G_{\mathrm{aq},\mathrm{calc}}^{\mathrm{act}}$ for TS4 (eq 5, blue pathway in Figure 2b). The $\text{Δ}G_{\mathrm{aq},\mathrm{calc}}^{\mathrm{act}}$ value for a single electron-transfer reaction was determined using Marcus theory to be 3.4 kcal/mol for TS5 (eq 4, green pathway in Figure 2b). In summary, dominant aqueous-phase reactions are shown in the green and blue pathways compared to the endergonic reaction forming peroxynitrite in the red pathway. These computations strongly support that the formation of peroxynitrite seems unlikely in accordance with the experimental observations above.

### 3.4 Formation of Nitro Compounds during Oxidation Processes

#### 3.4.1 Formation of Nitro Compounds during Ozonation, with Peroxynitrite and during γ-Radiolysis in Ultrapurified Water

The nitration potential of selected compounds during ozonation in the presence of nitrite, peroxynitrite, and γ-radiolysis/NO_2_^–^ was evaluated (for chemical structures, see Table S11). Table 1 shows the selected aromatic target compounds tested for nitro product formation at pH 8.2 for ozonation in the presence of nitrite and *t*-BuOH (first column), peroxynitrite (second column), and γ-radiolysis/NO_2_^–^ (third column). The ozonation experiments were performed with various molar ozone:nitrite ratios of 0–4 (experimental details in caption of Table 1). Several compounds, where no nitro products were detected upon ozonation and peroxynitrite exposure, were not tested in γ-radiolysis (1,2-benzothiazole-3­(2*H*)-one, 2,4,6-trimethylphenol, 1-methoxynaphthalene, 2-naphthoxyacetic acid, 2,7-naphthalenedisulfonic acid, butylhydroxytoluene, quinoline, hydroquinone, and trolox). For ranitidine, a nitro compound was not detected in any of the three reaction systems. Details about the detection conditions of the nitro compounds along with the extracted ion chromatograms and MS spectra are shown in Section S9. A clear distinctive pattern for the formation of nitro compounds among the selected chemical structures and reaction systems was not found. Since in the γ-radiolysis/NO_2_^–^ system a nitro compound was detected for all except one compound, nitro product formation is generally possible in a ^•^OH-dominated system, while the other oxidants have a somewhat higher selectivity.

**Table 1:**
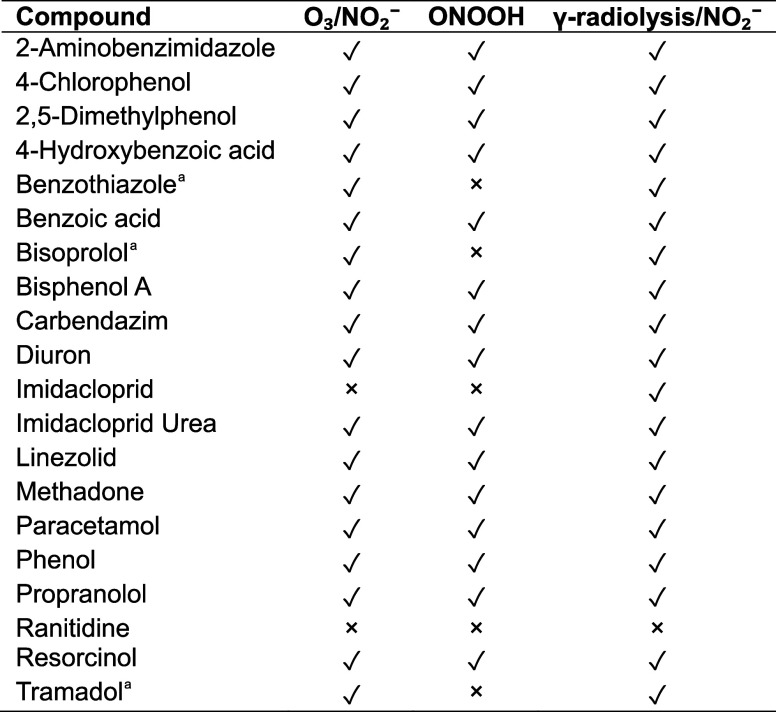
Nitro compound Formation from Selected Phenolic Compounds/Aromatic Micropollutants in Ultrapurified Water at pH 8 (10 mM or 50 mM Phosphate Buffer) under Various Conditions (Tick for Formation, Cross for No Formation)^b^

a Compounds with different outcomes for ozonation and peroxynitrite exposure.

b Selected phenolic compounds/aromatic micropollutants and the detected nitro compounds in either the ozone/nitrite system (O_3_/NO_2_^–^), in the presence of peroxynitrite (ONOO^–^), or in the γ-radiolysis/nitrite experiments. O_3_/NO_2_^–^: target compounds (compounds at 10 μM except phenol and butylhydroxytoluene at 100 μM), nitrite 10 μM (most compounds) or 100 μM (for phenol and butylhydroxytoluene), *t*-BuOH 15 mM (most compounds) or 150 mM (for phenol and butylhydroxytoluene), ozone doses 0, 10, 20, and 40 μM (most compounds) or 100 μM (for phenol and butylhydroxytoluene). ONOO^–^: target compounds (10 μM) peroxynitrite added at concentrations from 1 to 10 μM. γ-Radiolysis/NO_2_^–^: N_2_O/O_2_ 85%:15% (saturation), nitrite 0.67 mM, target compounds 10 μM, irradiation times 0–24 min. For structures of the precursor compounds, see Table S11.

For the majority of the selected model compounds, the reaction with peroxynitrite leads to the same nitro compounds as in the ozone/nitrite system (Table 1). Nevertheless, there were some differences: benzothiazole, bisoprolol, and tramadol did not produce nitro derivatives from the reaction with peroxynitrite, whereas in the ozone-nitrite system, trace amounts were detected (Table 1). These differences might be due to analytical sensitivities for the detection of these nitro compounds rather than due to a lack of formation. Overall, these results show that peroxynitrite has a similar potential for nitro compound formation as the combination of ozone with nitrite, with potential differences in selectivity. During ozonation and the application of peroxynitrite, ^•^NO_2_ is formed, which may be the crucial species for nitro compound formation.^71,72^ At pH 8 at which most experiments were performed, the half-life time of peroxynitrite is about 16 s (Table S4) and still controlled by ONOOH, which accounts for about 2.5% of the total peroxynitrite (p*K*_a_ = 6.8). The same formation pattern of nitro compounds in γ-radiolysis/nitrite experiments (Table 1, third column) and O_3_/NO_2_^–^ supports the role of ^•^NO_2_ as a driver for the nitration reactions, since the γ-radiolysis/nitrite experiments were optimized for ^•^NO_2_ formation (with high nitrite excess). Nevertheless, the ONOOH system also forms nitro compounds from most precursors in Table 1, and the pH-dependent nitro compound formation pattern will be investigated below to further distinguish potential formation pathways.

#### 3.4.2 Nitro Compound Formation during Secondary Wastewater Effluent Ozonation

Diuron and carbendazim were selected for a detailed study on nitro compounds formation because their nitro products have a good ionizability in HRMS allowing for sensitive detection (in contrast to, e.g., nitrophenols). Figure 3 shows the relative abatement of (a) diuron and (b) carbendazim and the formation of the corresponding nitro compounds (concentrations for nitrodiuron and peak areas for nitrocarbendazim in LC-HRMS/MS chromatograms) in secondary municipal wastewater effluent as a function of the specific ozone dose for different doses of nitrite (0.03 mg_N_/L (squares), 0.1 mg_N_/L (triangles), and 0.5 mg_N_/L (diamonds)). The abatement of the target compounds is shown in Figure 3 for 0.03 mg_N_/L. For all conditions shown in Figure 3, the abatement of the target compounds is controlled by ozone and a hydroxyl radical and not RNS. Very similar trends were observed for the other nitrite levels (Figure S10). Generally, a higher nitro compound formation can be observed for higher nitrite concentrations. This may be caused by a higher proportion of ozone and ^•^OH reacting with nitrite with the ensuing formation RNS. Both nitrodiuron and nitrocarbendazim go through maxima as a function of the specific ozone dose. This is likely caused by the interplay between abatement of the target compound and nitro compound formation and its further reaction to other products. For increasing specific ozone doses, the fractions of ozone and ^•^OH reacting with nitrite and diuron/carbendazim remain the same, but the absolute formation of RNS increases, whereas the diuron/carbendazim concentrations decrease. For a specific ozone dose of 1 gO_3_/g DOC, a significant abatement of carbendazim can be expected from its significant reactivity with ozone (*k*_O_3__ ∼ 5 × 10^3^ M^–1^ s^–1^, this study, Section S8).^12,73^ Therefore, for higher specific ozone doses, the formation of nitrocarbendazim decreases. A similar pattern is observed for diuron, despite its much lower ozone reactivity (*k*_O_3__ ∼ 15 M^–1^ s^–1^)^74–76^ compared to carbendazim. It is expected that the diuron abatement is mostly controlled by ^•^OH reactions leading to a very similar relative abatement (Figure 3a).^10,12,73^

**Figure 3:**
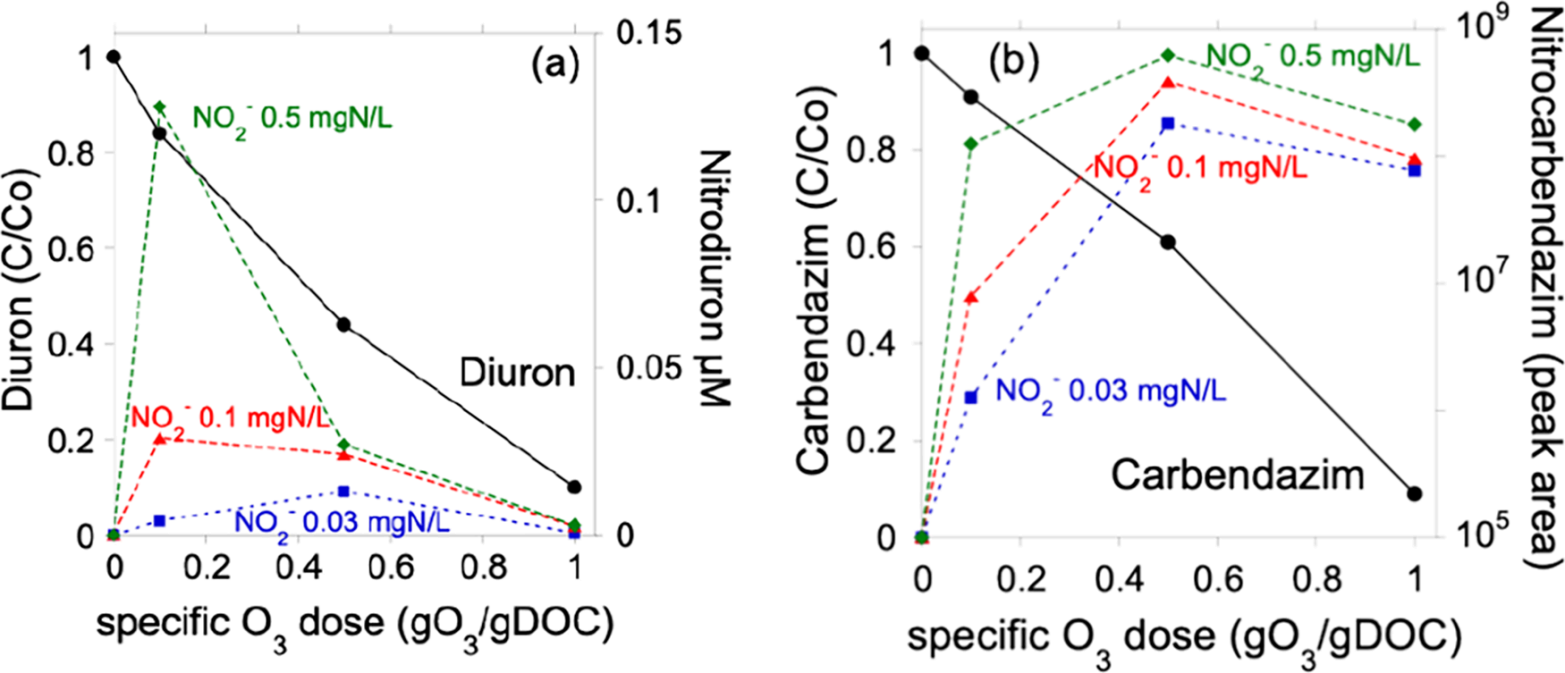
Transformation of (a) diuron and (b) carbendazim (black solid lines) and formation of the corresponding nitro compounds (colored dashed lines) during ozonation of secondary wastewater effluent (Table S3) as a function of the specific ozone dose. Concentrations are shown for nitrodiuron, peak areas are shown for nitrocarbendazim. The data for the abatement of the target compounds with a nitrite concentration of 0.03 mg_N_/L is shown, but it is very similar for all nitrite doses (Figure S10). Conditions: secondary wastewater effluent (DOC: 6 mg/L, alkalinity 5.8 mmol/L, pH 8.3), target compounds spiked at a concentration of 1 μM. Lines are shown to guide the eye.

### 3.5 Effect of pH on the Formation of Nitro Compounds in the Three Experimental Test Systems (O_3_/NO_2_^–^, ONOOH, γ-Radiolysis/NO_2_^–^)

The lack of peroxynitrite detection during nitrite ozonation experiments at an alkaline pH (pH 11) suggests that peroxynitrite is unlikely to be the driving nitrating agent. To corroborate these findings, the trend of formation of nitro compounds from the two selected precursors (diuron and carbendazim) was monitored in the pH range 6–12 in the three reaction systems. The two selected precursor compounds have different reactivities with ozone, which may influence nitro compound formation during ozonation due to different degrees of abatement of the target compounds (see below). Furthermore, the two selected compounds have distinct acid–base speciation, and therefore, further compounds should be tested in future investigations to get a full understanding of the pH dependence of nitro compound formation.

#### 3.5.1 Diuron

##### 3.5.1.1 O_3_/NO_2_^–^

As shown in Figure 4a (blue line), in the absence of an ^•^OH scavenger, the formation of nitrodiuron isomer-1 during ozonation of nitrite- and diuron-containing water increases only slightly from pH 6–9, with a more significant increase for higher pH values. A second isomer (isomer-2) with the nitro group in a different position (structures shown in Scheme 1) shows the same pattern (Figure S11a). This is consistent with an enhancement of the electron-transfer reaction between ozone and nitrite (eq 4), with a higher yield of ^•^NO_2_ for pH > 9 (Figure 1) yielding a stoichiometric formation of ^•^NO_2_ and ^•^OH (reactions 4, 6, and 7). Ozonation experiments were also performed in the presence of DMSO as a scavenger for ^•^OH (Figure S11a). The yields of nitrodiuron (relative to the initial diuron concentration) and relative residual diuron (relative to the initial diuron concentration, %) as a function of pH in the presence and absence of ^•^OH scavengers are shown in Figure S12. The nitrodiuron yield was significantly reduced in the presence of DMSO (0.06–0.7%) compared to that in the absence of DMSO (0.9–4.5%) (Figure S12a). This is accompanied by a lower diuron consumption in the presence of DMSO (0.6–8.6%) than in the absence of DMSO (11–16.8%) in the pH range 6–11 (Figure S12a). A similar experiment in the absence and presence of *t*-BuOH (as the ^•^OH scavenger) showed very similar trends for nitrodiuron formation (Figures S11b and S12b). Based on these findings, it can be concluded that ^•^OH plays a crucial role for the formation of nitro derivatives during ozonation of nitrite-containing waters.

**Figure 4:**
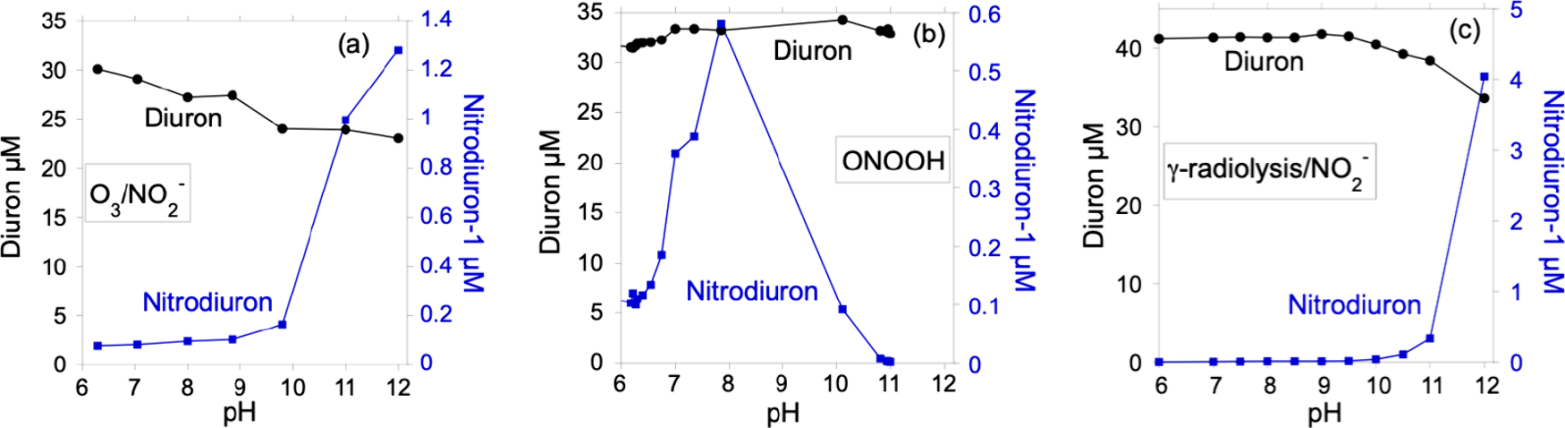
Residual diuron concentration (p*K*_a_ = 13.5) and the formed concentrations of nitrodiuron isomer-1 (two different positions of nitration, structures are shown in Scheme 1) as a function of the pH. Note that the initial diuron concentrations are in the range of 30–40 μM. The large differences in nitrodiuron formation are not caused by the relatively small variations in initial diuron concentrations. (a) Ozonation of nitrite-containing solutions in the absence of DMSO: diuron 34 μM; phosphate buffer 10 mM, nitrite 0.50 mM, ozone dose 0.25 mM; calculated fraction of ^•^OH scavenged by nitrite and diuron is approximately 99% and 1%, respectively. (b) Exposure to peroxynitrite in the absence of DMSO (black line): diuron 35.5 μM in phosphate buffer (50 mM), peroxynitrite 50 μM. Note that the formation of nitrodiuron only accounts for 2% of the initial diuron concentration and is therefore not visible in the diuron concentration profile. (c) γ-Radiolysis of nitrite- and diuron-containing solutions; phosphate buffer 10 mM, diuron 42 μM, nitrite 2 mM, solutions with percentage saturation N_2_O/O_2_ 85%:15%; irradiation time: 40 min. Estimated ^•^OH scavenging by nitrite and diuron are 99.6% and 0.4%, respectively. All solutions were stored overnight at room temperature in the dark before they were measured. The lines are shown to guide the eye.

**Scheme 1:**
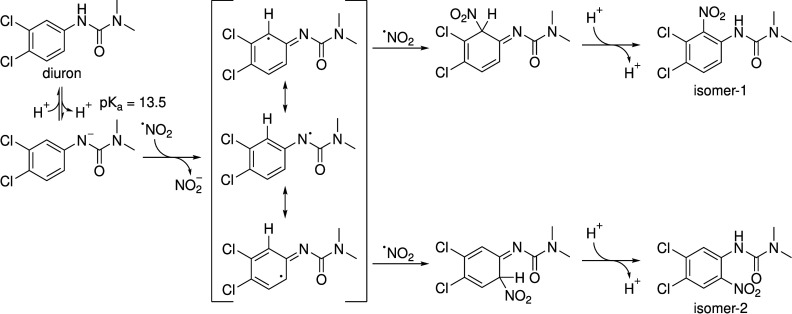
Nitrodiuron Formation from the Reaction of Diuron with Nitrogen Dioxide^a^

a Diuron reacts with an electron-transfer reaction with ^•^NO_2_ to a radical (resonance structures are shown), which reacts with a second ^•^NO_2_ to the two detected diuron isomers.

##### 3.5.1.2 ONOOH

Figure 4b shows the nitrodiuron formation (isomer-1) as a function of the pH for peroxynitrite dosing to diuron-containing solutions in the absence of an ^•^OH scavenger. The formation trend of nitrodiuron had a very different pH-dependence compared to ozonation with a maximum at pH 8, a decrease for pH > 8, and very little formation of nitrodiuron at pH 11. These findings can be explained by the pH-dependent mechanism of peroxynitrite decomposition and reactivity with the acidic form being the principal reacting species.^77^ No experiments could be done in the pH range 8–10, because of limitations of the used phosphate buffer (peroxynitrite stock solutions are highly alkaline; a borate buffer was not used since it interferes with peroxynitrite).^78^ Therefore, the nitrodiuron formation trend with peroxynitrite contrasts markedly from the ozone/nitrite system where at pH ≥ 10 nitrodiuron formation increased in the absence of DMSO (Figure 4a). In addition, the presence of DMSO had only a minor effect on the nitrodiuron formation by peroxynitrite, unlike during ozonation where the ^•^OH scavenger had a significant effect (Figures S11, S12, and S14). Overall, the different pH-dependent nitrodiuron formation pattern for peroxynitrite compared to ozonation experiments is a strong indication, that nitrodiuron might not be formed via peroxynitrite during ozonation. In addition, it was shown above that peroxynitrite could not be detected during ozonation. Furthermore, quantum chemical computations confirmed that its formation is kinetically/thermodynamically unfavorable.

##### 3.5.1.3 γ-Radiolysis/NO_2_^–^

During γ-radiolysis, ^•^NO_2_ is formed from the reaction of nitrite with ^•^OH (eq 3). The formation of nitrodiuron isomer-1 and isomer-2 (Scheme 1) increased significantly for pH > 9.5 (Figures 4c and S13). Since during γ-radiolysis the formation rate of ^•^NO_2_ is independent of the pH, this pH-effect might be related to the speciation of diuron with a p*K*_a_ of 13.5 (secondary amide function).^79^ At higher pH, a partial deprotonation of the amide group will enhance the electron density of the aromatic ring (Scheme 1). At pH 10, this can become important if the deprotonated diuron has a ^•^NO_2_-reactivity which is approximately 3000 times higher than diuron itself. Since ^•^NO_2_ is quite a selective oxidant, such differences seem reasonable.^80,81^ Because ^•^NO_2_ reacts mostly by electron-transfer reactions,^80,81^ it is hypothesized that ^•^NO_2_ attacks diuron forming a radical (similar to a previous study)^46^ which then combines with another ^•^NO_2_ to form the observed nitro compounds (Scheme 1). Hydroxyl radicals are quenched to 99% by nitrite and therefore not important under these conditions (caption Figure 4).

The pH-dependence trend of nitrodiuron formation during γ-radiolysis (Figures 4c and S13, 40 min irradiation time) is similar to ozonation, particularly in the absence of an ^•^OH scavenger (Figures 4a and S14). The similarities of pH-dependence for ozonation and γ-radiolysis experiments and the completely different trend for peroxynitrite suggest that ^•^NO_2_ may be the decisive species for nitrodiuron formation.

Alternatively, N_2_O_4_ which is formed by the self-reaction of ^•^NO_2_ (4.5 × 10^8^ M^–1^s^–1^) could be a potential nitrating agent.^82^ Aromatic compounds have second-order rate constants *k* for the reactions with ^•^NO_2_ on the order of 10^7^ M^–1^ s^–1^.^48^ Since the concentrations of the precursor compounds are in the range of 10–100 μM, it is unlikely that ^•^NO_2_ undergoes a self-reaction, because its steady-state concentration is about 2 × 10^–10^ M:

$$[{\mathrm{NO}}_{2}{]}_{\mathrm{ss}}=\frac{\mathrm{fr}}{k\times [\mathrm{TC}]}=\frac{2\times 10^{-8}}{10^{7}\times 10^{-5}}=2\times 10^{-10}\;M$$

fr: NO_2_ formation rate = 20 nm/s in γ-radiolysis;^83^ *k* = 10^7^ M^–1^s^–1^; TC: target compound concentration (10 μM). Therefore, N_2_O_4_ can be ruled out as an oxidant under our experimental conditions.

#### 3.5.2 Carbendazim

##### 3.5.2.1 O_3_/NO_2_^–^

Carbendazim has two p*K*_a_ values of 4.5 and 10.8,^84^ and only the latter is relevant for typical water treatment conditions (for acid–base speciation of carbendazim, see Scheme S1).^200^ The apparent second-order rate constants for the reaction of carbendazim with ozone were determined at pH 7, 7.5, and 8 in excess of ozone, and by extrapolation, the species-specific second-order rate constants can be determined (Section S8). Two species-specific second-order rate constants *k*_O_3__ values for the reaction with ozone could be determined as (4.3 ± 0.2) × 10^3^ M^–1^ s^–1^ and approximately 4 × 10^6^ M^–1^ s^–1^ for the protonated and deprotonated species, respectively (the uncertainty of the value of the deprotonated species is large due to the uncertainty of the p*K*_a_, see discussion in Section S8).

The relative residual concentration of carbendazim during the ozonation of nitrite-containing solutions as a function of pH is shown in Figure 5a. There is a gradual relative decrease in carbendazim with increasing pH. Even though there is an excess of nitrite relative to ozone and carbendazim (see Figure 5), for pH values above the p*K*_a_ of carbendazim, the fraction of ozone reacting with carbendazim can reach about 25%, which can be calculated based on the apparent second-order rate constants for the ozone reactions with carbendazim and nitrite. Considering the lower ozone stability at higher pH, a full degradation of carbendazim is not expected; nevertheless, a substantial abatement is still possible in comparison to that of diuron (Figure 4a). The resulting lower concentrations of the precursor carbendazim at higher pH lead to a lower formation of nitrocarbendazim. Furthermore, the nitro group might lower the p*K*_a_ of nitrocarbendazim relative to carbendazim, which may lead to a higher apparent ozone reactivity due to a higher fraction of the deprotonated form and therefore a higher consumption of nitrocarbendazim. In analogy, the reactivities of phenol and *p*-nitrophenol are similar at pH 8, despite the much higher species-specific second-order rate constant for the ozone reaction of the phenolate relative to the *p*-nitrophenolate.^85^ This effect is caused by a p*K*_a_ depression of 2.7 units from that of phenol (p*K*_a_ = 9.9) to that of *p*-nitrophenol (p*K*_a_ = 7.2).

**Figure 5:**
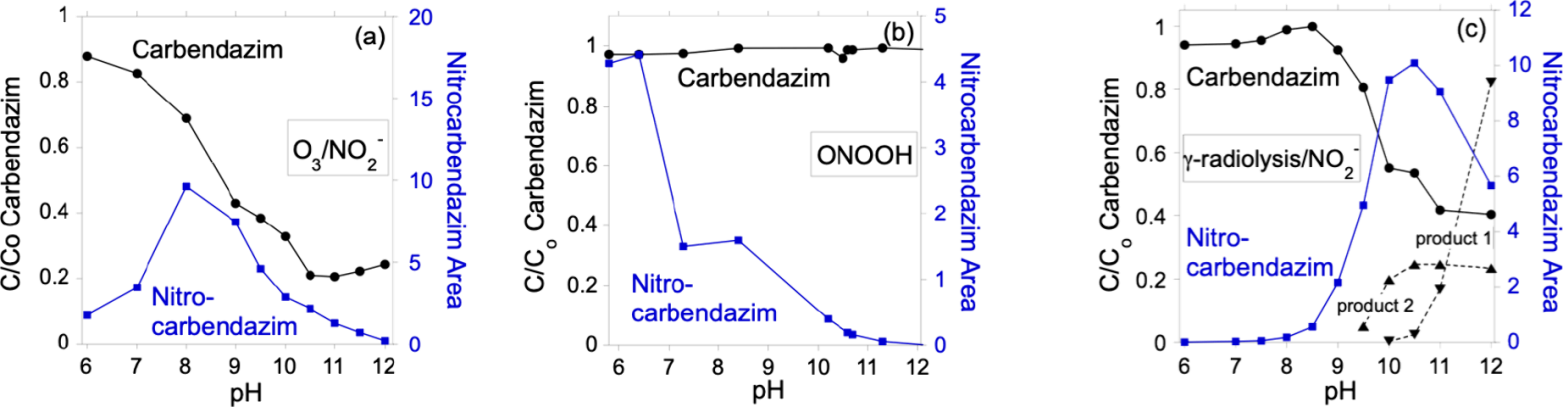
Relative residual concentrations of carbendazim (black) (p*K*_a_ = 10.8) and formation of nitrocarbendazim (blue) as a function of pH for (a) ozonation, (b) peroxynitrite, and (c) γ-radiolysis. Products 1 and 2 (γ-radiolysis samples, panel (c)) are shown in absolute areas represented by the left *Y*-axes (see Figure S16 for structure). (a) Ozonation of nitrite-containing solutions without DMSO: carbendazim 26 μM; phosphate buffer 10 mM, nitrite 0.50 mM, ozone dose 0.25 mM; calculated fraction of ^•^OH scavenging 99% and 1% by nitrite and carbendazim, respectively. (b) Exposure to peroxynitrite in the absence of DMSO: carbendazim (28 μM) in phosphate buffer (50 mM) and peroxynitrite (50 μM). Note that the formation of nitrocarbendazim only accounts a few % of the initial carbendazim concentration and is therefore not visible in the carbendazim concentration profile. (c) γ-Radiolysis of nitrite- and carbendazim-containing solutions: phosphate buffer 10 mM, carbendazim 14 μM, nitrite 2 mM, solutions with percentage saturation N_2_O/O_2_ 85%:15%; irradiation time = 40 min, estimated ^•^OH scavenging by nitrite 99.8% and carbendazim 0.2%. All solutions were stored overnight in the dark at room temperature before measurements. The lines are shown to guide the eye. Peak areas refer to measurements by HPLC-UV.

##### 3.5.2.2 ONOOH

For peroxynitrite, nitrocarbendazim formation had a maximum at low pH and decreased gradually with an increase in pH (Figure 5b). As discussed for diuron, this is most likely governed by the chemistry of peroxynitrite, which is a much better nitrating agent at low pH but has lower stability under such conditions. At higher pH, it has higher stability, but the nitration is less effective. Overall, the observed pattern is a result of the interplay between the reactivity and stability of peroxynitrite.

##### 3.5.2.3 γ-Radiolysis/NO_2_^–^

During γ-radiolysis, the nitrocarbendazim formation increased significantly with increasing pH in particular for pH > 8.5 (Figure 5c). This is similar to the evolution of nitrodiuron (Figure 4c). However, the nitrocarbendazim has a maximum formation at around pH 10.5 and decreases at higher pH levels in conjunction with a higher extent of carbendazim abatement. With a p*K*_a_ for carbendazim of 10.8, this might be related to a higher reactivity of the deprotonated form with ^•^NO_2_, leading to a faster abatement of the target compound. As a consequence, the nitrocarbendazim reaches concentrations in a similar range as carbendazim and will compete for radicals which lead to its abatement. This is accompanied by the formation of two new products (Products 1 and 2, Figure 5c, Product 1: carbendazim oxidation product, and Product 2: nitrocarbendazim degradation product, structures and annotated MS^2^ spectra are shown in Figures S16–S19). Product 1 is formed by the oxidation of carbendazim by ^•^OH (phenol formation is typical for ^•^OH reactions with aromatic compounds),^86^ while Product 2 is formed due to hydrolysis of nitrocarbendazim.

Overall, in contrast to diuron, in the case of carbendazim, the pH-dependent trends for nitrocarbendazim formation are more complex for a clear distinction between the three systems, which highlights the effects of compound properties on determining its reactivity with RNS. In the case of carbendazim, this can be attributed to a more complex reactivity with relatively high and pH-dependent ozone and radical reactivity.^16^

## 4 Proposed Nitration Reaction Mechanism

Scheme 2 shows the potential reaction pathways of reactive nitrogen species during ozonation of nitrite-containing solutions. Although nitrate is the main product of nitrite oxidation by ozone, nitrogen dioxide can be formed by ozone and by a hydroxyl radical. Peroxynitrite formation by ozone has also been suggested previously. In this study, the formation of nitro products from diuron and carbendazim was investigated by three different systems: O_3_/NO_2_^–^, peroxynitrite, and ^•^OH/NO_2_^–^. During ozonation in this study, peroxynitrite formation was not observed, and its formation seems thermodynamically unfavorable. Furthermore, the significantly different pH-dependent pattern of nitro compound formation for peroxynitrite compared to the other two reaction systems suggests that peroxynitrite is not the main reactant for nitro product formation. Since in both the O_3_/NO_2_^–^ and ^•^OH/NO_2_^–^ systems ^•^NO_2_ is formed, it is hypothesized its reaction with aromatic compounds is the main pathway to nitro compound formation. Based on the lower extent of formation of nitro products during ozonation in the presence of a hydroxyl radical scavenger and the observed nitro compound formation in γ-radiolysis experiments, it is further hypothesized that oxidation of nitrite by ^•^OH to ^•^NO_2_ is key for the formation of nitro compounds during ozonation.

**Scheme 2:**
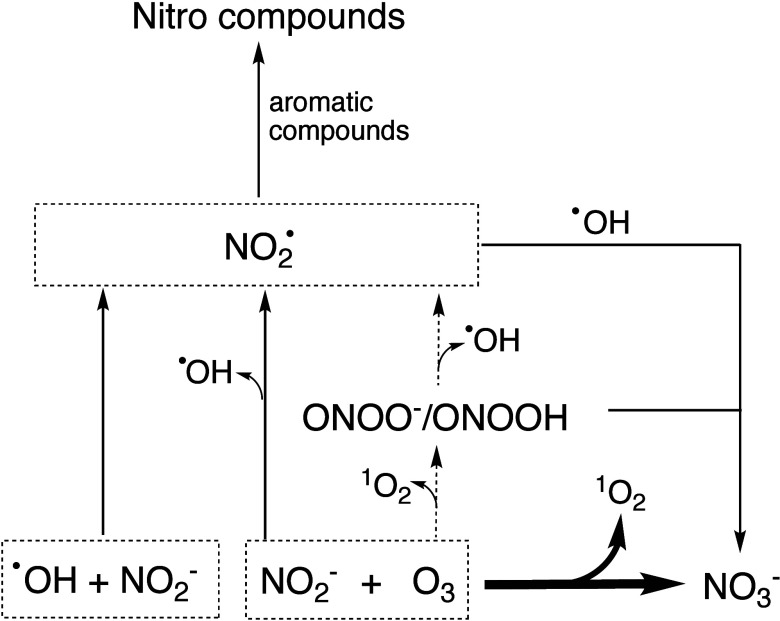
Proposed Reaction Mechanisms for the Formation of Nitrogen Dioxide (Solid Lines) and the Unlikely Formation of Peroxynitrite (Dashed Line) during Ozonation En Route to Nitro Compounds^87^

Peroxynitrite also decomposes to ^•^NO_2_ and could therefore contribute to this pathway as well. Since peroxynitrite could not be detected even at an ozone dose of 700 μM with a detection limit of 1 μM (Figure S6), its yield is <0.2%. This would result in 0.5 μM under typical ozonation conditions as provided in Figure 4. From Figure 1, we concluded that the ^•^NO_2_ yield from the ozone reaction with nitrite is at the minimum 10%, which would result in integrated concentrations of 25 μM not even including the contribution of hydroxyl radicals. In addition, peroxynitrite is consumed quickly by ozone. Overall, these estimates demonstrate that peroxynitrite can not contribute more than 2% to the overall ^•^NO_2_ formation even if optimistic scenarios are considered.

## 5 Practical Implications

The present study elucidates the mechanisms through which the ozonation of nitrite-containing waters leads to the formation of nitro compounds. To avoid the formation of these undesired products, it is important to perform a full nitrification before ozonation of secondary municipal wastewater effluents, even though it is tempting to oxidize nitrite with ozone to nitrate. Although the majority of nitrite reacts with ozone to nitrate by an oxygen-transfer reaction, a minor fraction undergoes an electron-transfer reaction with ozone and hydroxyl radical forming ^•^NO_2_, which appears to be the main nitrating species in the ozone-nitrite system.

This minor pathway can have practical implications due to the formation of nitro compounds, because of their potential toxicological relevance. Therefore, an understanding of the main chemical reactive species in the ozone/nitrite system is crucial for predicting the formation of nitro compounds and factors which affect it. The nitro compounds formed during wastewater ozonation are potentially responsible for an observed enhanced mutagenicity formation reported in the presence of nitrite. The formation yields of nitro compounds observed in this study were low, with a maximum conversion of diuron to nitrodiuron of approximately 0.2% under wastewater ozonation conditions. For typical micropollutant concentrations in secondary effluents (0.1–1 μg/L), this corresponds to estimated concentrations of individual nitro products in the low ng/L range. In a previous study, it has been demonstrated that the total concentration (list of 550 target compounds) of micropollutants in a secondary wastewater effluent before ozonation is about 50 μg/L.^12^ If we assume a similar average relative nitro compound formation of 0.2%, this yields a total concentration of about 0.1 μg/L. After a 1000-fold enrichment of wastewater (performed during sample preparation for Ames microplate format (MPF) assay), these concentrations would fall in a μg/mL range in the extract used for the Ames MPF assay. These concentrations are in the lower range in which mutagenicity is commonly detected for mutagenic chemicals tested individually in the Ames MPF assay including nitro compounds such as 2-nitropropane and 2,4-dinitrotoluene.^88^ Given the uncertainty of such an estimation, detection of mutagenicity cannot be excluded. However, further investigations are needed to address this issue.

Nitrite oxidation also occurs during other oxidation/disinfection processes including chlorination, application of chlorine dioxide, and advanced oxidation processes,^46,89,90^ and the formation of the ensuing nitrophenols and nitroanilines has been reported in the presence of chlorine.^30,46^ Nitrite oxidation by ^•^OH to ^•^NO_2_ may lead to nitro compounds in nitrite-/nitrate-containing waters in the advanced oxidation process UV/H_2_O_2_. Also, other radical-based processes involving halogen-containing radicals could lead to the formation of nitro compounds in the presence of nitrite. UV-based processes during which nitrate photolysis occurs have also a high potential for the formation of nitro compounds^91^ but additionally lead to nitrite, which can lead to further nitro compound formation during postchlorination.

## Supplementary Material



## Notes

Reagents, chemicals, instruments and analytical methods, wastewater composition, formation of nitro compounds from model compounds, peroxynitrite reactivity, peroxynitrite formation during ozonation, reactivity of peroxynitrite with ozone, hydroxyl radical yield from the ozone-nitrite reaction and peroxynitrite decomposition, diuron and carbendazim abatement during ozonation of nitrite-containing wastewater, formation of nitrodiuron in nitrite-containing waters, formation of nitrocarbendazim and other transformation products in nitrite-containing waters, determination of the second-order rate constant for the reaction of ozone with carbendazim, MS spectra of nitro products, and NMR experiments (PDF)
