# Supplementary material for: Nitrite Oxidation during Ozonation Revisited: Mechanisms of Nitration Reactions
Source: Environ Sci Technol. 2026 Jun 18;60(25):18270–82. doi: 10.1021/acs.est.6c02098 (PMC13325883; doi:10.1021/acs.est.6c02098)
Supplement: Supplementary file 1 [file es6c02098_si_001.pdf]

# Supporting Information

# Nitrite oxidation during ozonation revisited: Mechanisms of nitration reactions

Tarek Manasfi[1], Christoph Dieziger[1], Simon A. Rath[1], Daisuke Minakata[2], Urs von Gunten[1,3*]

[1]Swiss Federal Institute of Aquatic Science and Technology (Eawag), 8600 Dübendorf, Switzerland

[2]Department of Civil, Environmental, and Geospatial Engineering, Michigan Technological University, Houghton, MI 49931, US

[3]School of Architecture, Civil and Environmental Engineering (ENAC), Ecole Polytechnique Fédérale de Lausanne (EPFL), 1015 Lausanne, Switzerland

* Corresponding author: Urs von Gunten, vongunten@eawag.ch

# Table of Contents


S1 Chemicals, reagents, samples, methods and instruments ..... 3
S1.1 Reagents, chemicals, stock solutions, and instruments ..... 3
S1.1.1 Synthesis of nitro diuron isomers ..... 4
S1.1.2 Micropollutant Stock Solutions ..... 4
S1.1.3 Generation of ozone stock solutions ..... 4
S1.1.4 Instruments ..... 5
S1.2 Sample collection and preparation ..... 5
S1.2.1 Wastewater Samples ..... 5
S1.2.2 Formation of nitro compounds from model compounds ..... 5
S1.3 Sample analyses ..... 6
S1.3.1 HPLC-UV ..... 6
S1.3.2 LC-HRMS ..... 7
S1.4 Measurement of nitrate and nitrite ..... 8
S2 Peroxynitrite reactivity under water treatment conditions ..... 9
S2.1 Influence of pH ..... 9
S2.2 Influence of dissolved $CO_2$ ..... 10
S2.3 Ionic strength ..... 12
S2.4 Influence of nitrite ..... 13
S3 Assessment of peroxynitrite formation during ozonation of nitrite-containing solutions ..... 16
S3.1 Testing of peroxynitrite formation by direct measurement ..... 16
S3.2 Determination of the second-order rate constant of the reaction of ozone with peroxynitrite 18
S4 Hydroxyl radical yield ..... 20
S4.1 Determination of hydroxyl radical yields during ozonation of nitrite ..... 20
S4.2 Hydroxyl radical yield from the decomposition of peroxynitrite ..... 21
S5 Abatement of diuron and carbendazim during wastewater ozonation in presence of nitrite ..... 22
S6 Formation of nitrodiuron and nitrocarbendazim in the three investigated reaction systems ..... 23
S6.1 Diuron ..... 23
S6.1.1 $O_3/NO_2^-$ ..... 23
S6.1.2 γ-radiolysis/$NO_2^-$ ..... 25
S6.1.3 Nitro-diuron yields in the three reaction systems ..... 25
S6.2 Carbendazim ..... 26
S7 Carbendazim byproducts ..... 27
S8 Determination of the second-order rate constant for the ozone reaction with carbendazim ..... 29
S9 MS Spectra of nitro products ..... 32
S10 NMR Experiments ..... 43
S11 References ..... 48

# S1 Chemicals, reagents, samples, methods and instruments

## S1.1 Reagents, chemicals, stock solutions, and instruments

Table S1 provides a list of chemicals and reagents used in this study.

**Table S1** – Alphabetical list of reagents and chemicals used in this study.

| Chemical | Formula | Purity | Supplier |
|---|---|---|---|
| Acetic acid | $C_2H_4O_2$ | 100% (glacial) | Merck |
| Acetylacetone | $C_5H_8O_2$ | ≥99.9% | Sigma-Aldrich |
| Ammonium acetate | $C_2H_7NO_2$ | ≥98% | Sigma-Aldrich |
| Argon | Ar | 99.99% | Alphagaz |
| Benzaldehyde | $C_7H_6O$ | ≥99.9% | Sigma-Aldrich |
| Benzoic acid | $C_7H_6O_2$ | ≥99.5% | Fluka |
| Butylhydroxytoluene | $C_{15}H_{24}O$ | ≥99% | Sigma-Aldrich |
| 4-Chlorophenol | $C_6H_5ClO$ | ≥98% | Fluka |
| trans-Cinnamic acid | $C_9H_8O_2$ | ≥99.9% | Sigma-Aldrich |
| 2,5-Dimethylphenol | $C_8H_{10}O$ | ≥99% | Sigma-Aldrich |
| Dimethyl sulfoxide | $C_2H_6SO$ | ≥99.9% | Sigma-Aldrich |
| Formaldehyde | $CH_2O$ | 32.9% (titration) | Sigma-Aldrich |
| Hydrochloric acid | HCl | 32% | Merck |
| Hydroquinone | $C_6H_6O_2$ | ≥99% | Sigma-Aldrich |
| 4-Hydroxybenzoic acid | $C_7H_6O_3$ | ≥99% | Sigma-Aldrich |
| Methanol | $CH_4O$ | HPLC grade | Fisher Chemical |
| 1-Methoxynaphthalene | $C_{11}H_{10}O$ | ≥98% | Sigma-Aldrich |
| *N*-(1-Naphthyl)ethylene- | $C_{12}H_{14}N_2 \cdot 2HCl$ | ≥98% | VWR |
| Ultrapure water | $H_2O$ | >18 MΩ·cm | Barnstead, Diamond |
| Oxygen | $O_2$ | 99.99% | Carbagaz |
| Peroxynitrite | ONOO- | 160–200 mM in NaOH 4.7% | Merck |
| Phenol | $C_6H_6O$ | ≥99.5% | Thermo Scientific |
| Phosphoric acid | $H_3PO_4$ | ≥85% | Sigma-Aldrich |
| Quinoline | $C_9H_7N$ | 98% | Sigma-Aldrich |
| Resorcinol | $C_6H_6O_2$ | ≥98% | Sigma-Aldrich |
| Sodium bicarbonate | $NaHCO_3$ | ≥99% | Merck |
| Sodium chloride | NaCl | ≥99% | Merck |
| Sodium hydroxide | NaOH | 40% (v/v) | Merck |
| Sodium nitrate | $NaNO_3$ | 99.0% | Merck |
| Sodium nitrite | $NaNO_2$ | 99.0% | Merck |
| Sodium phosphate monobasic | $NaH_2PO_4 \cdot H_2O$ | ≥99% | Merck |
| Sodium phosphate dibasic | $Na_2HPO_4 \cdot 2H_2O$ | ≥98.5% | Sigma-Aldrich |
| Sulfanilamide | $C_6H_8N_2O_2S$ | ≥99% | Sigma-Aldrich |
| *tert*-Butanol | $C_4H_{10}O$ | ≥98.5% | Sigma-Aldrich |
| Tramadol hydrochloride | $C_{16}H_{25}NO_2 \cdot HCl$ | ≥99% | Sigma-Aldrich |
| 2,4,6-Trimethylphenol | $C_9H_{12}O$ | ≥97% | Sigma-Aldrich |
| Trolox | $C_{14}H_{18}O_4$ | ≥97% | Sigma-Aldrich |

### S1.1.1 Synthesis of nitro diuron isomers

Nitrodiuron isomers were synthesized by direct nitration of diuron according to the procedure described by Nélieu et al. (2010).[1] Nuclear magnetic resonance (NMR) confirmed the identity of the two nitrodiuron isomers which were formed in a relative abundance of 2.4:10 (Isomer-1: Isomer-2) (Figure S1). Details on the performed NMR experiments and the assignment of compounds in the synthesis product mixture based on $^{1}H$ and $^{13}C$ NMR chemical shift are provided in Section S10.

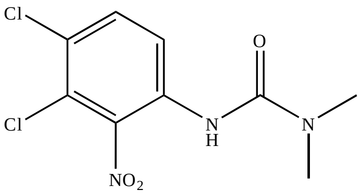


3-(3,4-dichloro-2-nitrophenyl)-1,1-dimethylurea
Nitrodiuron Isomer 1

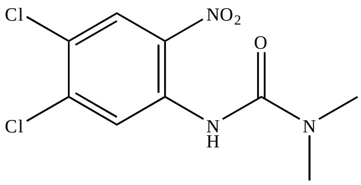


3-(4,5-dichloro-2-nitrophenyl)-1,1-dimethylurea
Nitrodiuron Isomer 2

**Figure S1** – Structures of the synthesized nitrodiuron isomers-1 and -2.

### S1.1.2 Micropollutant Stock Solutions

Stock solutions of organic compounds were prepared in ethanol or acetonitrile at a concentration of 1 g/L for the compounds summarized in Table S2. For oxidation experiments, the solvents were removed by evaporation (see details below).

**Table S2** – List of compounds sorted by functional groups and their final concentrations (see footnote) for experiments with ozone, peroxynitrite, and $\gamma$-radiolysis.

| Functional Group | Compounds[a] |
|---|---|
| Amines | 2-aminobenzimidazole, bisoprolol, methadone, propranolol, tramadol |
| Heterocycles | 1,2-benzothiazole-3(2H)-one[b], benzothiazole, carbendazim, linezolid, ranitidine, quinoline[b] |
| Benzoic acid derivatives | benzoic acid, 4-hydroxybenzoic acid |
| Naphtalene derivatives | 1-methoxynaphtalene[b], 2-naphthoxyacetic acid[b], 2,7-methoxynaphthalenedisulfonic acid[b] |
| Urea derivatives | diuron, imidacloprid urea |
| Phenol derivatives | bisphenol A, butylhydroxytoluene[b], 4-chlorophenol, hydroquinone[b], paracetamol, phenol, resorcinol, 2,4,6-trimethylphenol[b], trolox[b] |

[a] Concentration during spiking was 10 µM, except for phenol and butylhydroxytoluene which were spiked at 100 µM
[b] Compounds tested only in ozonation and peroxynitrite exposure experiments

### S1.1.3 Generation of ozone stock solutions

An ozone ($O_3$) generator (BMT 803 BT, BMT Messtechnik, Berlin) producing ozone-containing gas from pure oxygen (Carbagas, 99.995%) was used. The ozone stock solutions were prepared by sparging the generated ozone/oxygen gas mixture into ice-cooled ultrapure water. [2] The concentrations of the

ozone stock solutions were in the range of 1.1-1.5 mM and were determined directly with a spectrophotometer (Cary 100, Varian, USA) at 260 nm, with a molar absorption coefficient of ε = 3200 $M^{-1}$ $cm^{-1}$. [3]

### S1.1.4 Instruments

The following instruments were used in this study: UV-VIS Spectrophotometer Cary 100, Varian, USA), Ozone generator (BMT 803 BT, BMT Messtechnik, Berlin), HPLC-DAD (Dionex, Ultimate 3000), HPLC-HRMS (Dionex Ultimate 3000 - Q Exactive Plus, Thermo Scientific), pH Meter (Metrohm seven multi). For analytical methods see below, Section S1.3.

## S1.2 Sample collection and preparation

### S1.2.1 Wastewater Samples

**Table S3** – Water quality parameters of the collected municipal wastewater (WW) effluent sample for ozonation experiments with diuron and carbendazim.

| Experiment | Matrix | pH | DOC (mg/L) | Alkalinity (mmol/L) | $NH_4^+$ (µgN/L) | $NO_2^-$ (µgN/L) |
|---|---|---|---|---|---|---|
| Diuron/carbendazim ozonation | Secondary wastewater effluent | 8.3 | 6 | 5.8 | 16 | 30 |

### S1.2.2 Formation of nitro compounds from model compounds

A selection of organic compounds including phenols, benzoic acid derivatives and other compounds with different functional groups (amines, heterocycles, naphthalene derivatives, and urea derivatives) (Table S2) were tested to examine their potential to form nitro compounds in the three reaction systems (ozone/nitrite, peroxynitrite, γ-radiolysis/nitrite).

**Ozone/nitrite**

Stock solutions (1 g/L) of model compounds were used to achieve a final concentration of 10 µM in phosphate buffer (10 mM, pH 8) in 10 mL glass vials. Nitrite was also spiked with a dose of 10 µM. For compounds for which stock solutions where in organic solvents (methanol or acetonitrile), an aliquot was added to the glass vial and a gentle flow of $N_2$ was applied for 10-15 min (until complete solvent evaporation) before the addition of the phosphate buffer. After addition of the other components (nitrite, *t*-BuOH or DMSO in samples with hydroxyl radical scavenger) the samples were mixed well. Finally, aliquots of an ozone stock solution (Section S1.1) where added to the samples under mixing.

**Peroxynitrite**

Model compounds (details about identity and concentrations in Table S2) were exposed to different doses of peroxynitrite (0.1, 0.5, 1, 2, 5, 10, 50, 100 μM, with the two highest concentrations applied only for compounds spiked at 100 μM). An appropriate volume of the corresponding stock solution of each compound was spiked into a glass vial and dissolved in phosphate buffer (50 mM, pH 8). For compounds with stock solutions in an organic solvent (methanol or ethanol), the solvent was evaporated by applying a gentle nitrogen flow into the glass vial for 10-15 min (until complete evaporation). Stock solutions (1 mM and 50 μM) of peroxynitrite were prepared in NaOH (1 mM) from a commercial peroxynitrite solution (160-200 mM) (Table S1). After spiking peroxynitrite, solutions were stirred for at least 5 min and the final pH was measured. A significant pH increase (pH ≥ 9) was observed only in solutions spiked with a peroxynitrite concentration ≥ 10 μM.

**γ-radiolysis**

To air-saturated nitrite solution (100 μL) and oxygen-saturated ultrapurified water (200 μL), an aliquot (1.2 mL) of a $N_2O$-saturated solution containing model compounds in phosphate buffer (12.5 mM, pH 8 for all model compounds except for carbendazim and diuron for which buffers were at pH 6-12) was added. This mixture resulted in solutions with a percentage saturation of approximately 85:15% $N_2O$:$O_2$ (corresponds to a concentration of 20.4 and 0.195 mmol/L for $N_2O$ and $O_2$ respectively) and a final phosphate buffer concentration of 10 mM. Samples were treated by γ-radiolysis (Gammacell 220, Atomic Energy of Canada, Ltd., for dose rate see main text) with different irradiation times (0, 4, 6, 8, 20, 40 min). After irradiation, samples were stored overnight in the dark at room temperature before analyses by HPLC-UV and/or HPLC-HRMS.

## S1.3 Sample analyses

### S1.3.1 HPLC-UV

The second-order rate constant for the reaction between ozone and peroxynitrite was measured by competition kinetics with cinnamic acid as a competitor. Cinnamic acid and its ozone reaction product benzaldehyde were quantified using high-performance liquid chromatography (HPLC) coupled to a diode array detector (DAD) (Dionex Ultimate 3000). The separation process utilized a reversed-phase Atlantis column (2.1 mm × 150 mm, 3.5 μm) with a guard column, maintained at 30 °C. The flow rate was set at 0.3 mL/min under isocratic conditions, with the mobile phase comprising 60% of a 50 mM phosphoric acid solution in ultra-purified water at pH 2.2, and 40% methanol. The running time of the chromatographic method was 20 min and each sample was kept at 4 °C in the autosampler before injection (injection volume 100 μL). Cinnamic acid and benzaldehyde were detected at 254 nm. Quantification was performed based on external calibration in the range of 0.5 -100 μM for 5-2000 μM with LOQs of 0.1 and 1 μM for benzaldehyde and cinnamic acid, respectively.

For the determination of the second-order rate constant for the reaction of carbendazim with ozone (Section 8, SI), carbendazim and benzaldehyde (from quenching the reaction with cinnamic acid) were analysed by a HPLC (Ultimate 3000, Thermo) equipped with DAD. Chromatographic separation was performed using a COSMOSIL 5C18-MS-II (3.0 × 150 mm, 5 µm) column equipped with a guard-column at 25 °C. The flow rate was set at 0.6 mL/min and the following gradient method was applied: Start conditions: 20% methanol, 80% 10 mM $H_3PO_4$; 0.5 min: 20% methanol, 80% 10 mM $H_3PO_4$; 7.5 min: 60% methanol, 40% 10 mM $H_3PO_4$; 9.5 min: 60% methanol, 40% 10 mM $H_3PO_4$; 10.5 min: 20% methanol, 80% 10 mM $H_3PO_4$; 13 min: stop run. Each sample was kept at 10 °C in the autosampler before injection (injection volume 100 µL). Benzaldehyde (RT=8.0 min) was detected at 250 nm and carbendazim (RT=3.5 min) at 275 nm. Quantification was performed based on external calibration in the range of 0.5 to 5 µM for benzaldehyde and 0.05 to 0.5 µM for carbendazim. LOQs were determined as 0.01 and 0.02 µM for benzaldehyde and carbendazim, respectively.

### S1.3.2 LC-HRMS

1.4 mL of sample was added to HPLC vials with 0.1 mL internal standard mix containing 150 tramadol-d6 and diuron-d6 (C = 150 µg/L). Samples were analyzed by HPLC (Dionex Ultimate 3000) coupled to a high-resolution hybrid quadrupole-orbitrap mass spectrometer (Q Exactive Plus, Thermo Scientific) equipped with an electrospray ionization interface (ESI). Separation was performed using a reversed-phase column (Atlantis 2.1 mm × 150 mm, 3.5 µm particle size with guard column) at 30 °C, with a flow rate of 0.3 mL/min and an injection volume of 100 µL. Eluents used were (A) ultrapurified water + 0.1 % formic acid and (B) methanol + 0.1 % formic acid. Samples were measured in both positive and negative modes. All data evaluation was performed with the software Thermo Xcalibur™ and Thermo Freestyle™. All integrated areas were normalized with the corresponding internal standards (diuron-d6 for negative-, tramadol-d6 for positive mode) to account for ionization variation of the device during the measurement.

The LC method consisted of a gradient starting with 95% solvent A for the first minute, followed by an increase in solvent B from 5% to 95% over 16 minutes, maintaining this composition until 25 min. Subsequently, the system was returned to the initial conditions of 95% A and 5% B for 2 mins, holding for an additional 2 min to stabilize. To prevent phosphate buffer from contaminating the mass spectrometer, the flow was directed to waste during the initial 5 min.

Analyte ionization was performed using an electrospray ionization (ESI) source, set at a spray voltage of 4 kV and a capillary temperature of 320 °C. The mass spectrometer operated in both positive and negative modes, scanning a mass range of 100 to 1000 m/z in full-scan mode. Prior to measurements, mass calibration in the positive and negative modes was achieved using an amino acid solution,

achieving a mass accuracy below 5 ppm. During measurements, samples were kept at 4 °C in the autosampler.

## S1.4 Measurement of nitrate and nitrite

Nitrate levels were quantified by ion chromatography at the AuA Laboratory, Eawag, with a LOQ of 0.1 mg $NO_3$-N/L.

For measuring nitrite concentrations, the Griess method was used.[4] A 0.2 mL sample was first diluted to 10 mL with ultra-purified water. To this end, 0.2 mL of a sulfanilamide solution (10 mg/mL) was added and allowed to react for 10 minutes. Then, 0.2 mL of a *N*-(1-napthyl)-ethylenediamine-dihydrochloride solution (1 mg/L) was mixed in, resulting in the formation of a pink-coloured compound. After a 10-minute reaction period, the absorbance was recorded at 543 nm in a 1-cm quartz cuvette. Nitrite concentrations were calculated using an external standard calibration with a LOQ = 0.3 µM.

# S2 Peroxynitrite reactivity under water treatment conditions

The influence of multiple factors including pH, the presence of dissolved $CO_2$, ionic strength, and nitrite concentration on the peroxynitrite stability was investigated. The absorbance of peroxynitrite at 302 nm ($\varepsilon_{ONOO-}$ = 1705 $M^{-1}cm^{-1}$)[5] was monitored in a spectrophotometer using 1-cm quartz cuvettes. When peroxynitrite decayed rapidly, absorbance readings were taken continuously until stabilization. At conditions for which peroxynitrite was stable for hours, the absorbance was measured at specific time points until it dropped to at least 50% of its initial value. The apparent first-order rate constants for peroxynitrite decay ($k_{obs}$, $s^{-1}$) were determined by plotting the natural logarithm of the relative absorbances ($abs/abs_0$) as a function of time (s) and calculating the slope which corresponds $k_{obs}$ by linear regression. The average and standard deviation of these apparent first-order rate constants were then computed from triplicate measurements. From the apparent first-order rate constants ($k_{obs}$), the half-life time of peroxynitrite was calculated by eq. S1:

$$t_{1/2} = \ln 2 / k_{obs} \qquad \text{(S1)}$$

## S2.1 Influence of pH

The lifetime of peroxynitrite in phosphate-buffered water was determined using a UV-VIS spectrophotometer at various pH values at room temperature. Four phosphate buffers (70 mM) at pH 6.2, 7.2, 8.2, and 11.3 and an ionic strength of approximately 0.3 M, were prepared. For pH 6.2 -10.0, a peroxynitrite stock solution (300 µL, 5 mM in 25 mM NaOH) was diluted in each buffer solution (2700 µL) to a final concentration of about 0.5 mM peroxynitrite in a 1-cm quartz cuvette. Upon addition of peroxynitrite (alkaline stock solution), the buffer at pH 8.2 increased to pH 9, which enabled to make a measurement at this pH. To maintain pH 8.2, a 25 mM HCl solution (300 µL) was combined with the phosphate buffer (2400 µL) prior to the addition of peroxynitrite. For pH 11.3, peroxynitrite stock solution was mixed with the phosphate buffer at pH 11.3. For pH 12, 5 mM NaOH solutions were used. An additional control solution was prepared using the same buffer which was sparged with argon for 20 minutes to remove dissolved $CO_2$. The decay of peroxynitrite was monitored by measuring absorbance in spectrophotometry at 302 nm at reaction times of 0, 30, 60, 120 and 240 min. All samples were prepared and measured in triplicate.

The apparent first-order decay rate constants for peroxynitrite are presented in Table S4 and Figure S2. Except for the experiments at pH 6.2, which exhibited a very fast peroxynitrite loss, it was possible to measure peroxynitrite absorbance and its exponential decrease over time. These measurements were restricted to approximately 5 to 10 s needed for mixing of the solutions with a pipette before starting the measurements in the cuvette. According to Molina et al. 2013[6], the expected half-life time of peroxynitrous acid is less than 1 s. As the pH increased from 7.2 to 11, the apparent first-order decay

rate constants decreased by an order of magnitude per pH unit increase. Correspondingly, the half-life time increased with pH, from 4.1 s at pH 7.2 to over 11'000 s (approximately 3 h) at pH 12. The observed half-live times at pH 7.2 (2.2 s) and 8.2 (9.4 s) were roughly a factor of two higher than those reported by Molina et al. 2013.[6] This difference might be explained by the slightly elevated ionic strength of 0.3 M in our experimental setup.

**Table S4** – pH-dependence of apparent first-order decay rate constants and their standard deviations (σ) of peroxynitrite (0.5 mM) in phosphate buffer (70 mM, I = 0.3 M) and the corresponding half-life times ($t_{1/2}$). The pH-dependent half-life times for peroxynitrite for reaction with ozone with an ozone dose of 0.25 mM are also shown.

| pH | $k_{obs}$ ($s^{-1}$) | σ ($s^{-1}$) | $t_1/_2$ (s) | $t_1/_2$ (s) (0.25 mM $O_3$) |
|---|---|---|---|---|
| 6.2 | n.d. | n.d. | n.d. | ~ $2 \times 10^{-3}$ |
| 7.2 | $1.70 \times 10^{-1}$ | $7.48 \times 10^{-3}$ | 4.1 | ~ $8 \times 10^{-4}$ |
| 8.2 | $4.26 \times 10^{-2}$ | $1.14 \times 10^{-3}$ | 16.3 | ~ $6 \times 10^{-4}$ |
| 9 | $6.23 \times 10^{-3}$ | $4.34 \times 10^{-4}$ | 111 | ~ $5 \times 10^{-4}$ |
| 10 | $1.35 \times 10^{-3}$ | $5.67 \times 10^{-5}$ | 512 | |
| 11.3 | $6.32 \times 10^{-5}$ | $4.34 \times 10^{-6}$ | $1.06 \times 10^{4}$ | |
| 12 | $6.04 \times 10^{-5}$ | $4.48 \times 10^{-6}$ | $1.15 \times 10^{4}$ | |

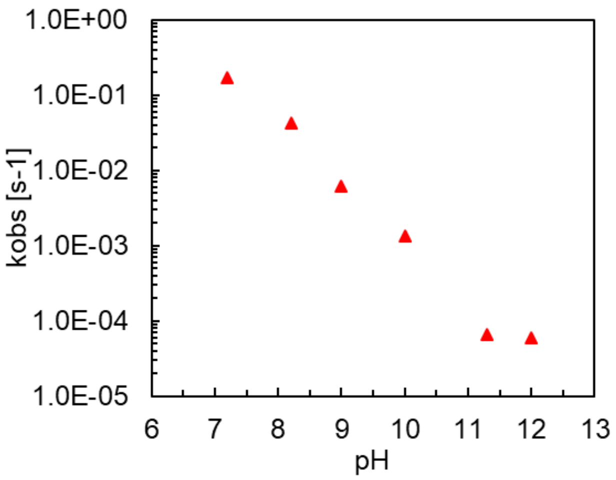


**Figure S2** – Apparent first-order decay rate constants of peroxynitrite (0.5 mM) as a function of the pH (phosphate buffer, 70 mM, I = 0.3 M).

## S2.2 Influence of dissolved $CO_2$

Peroxynitrite ($ONOO^-$) is known to rapidly react with $CO_2$ with a second-order rate constant ($k_{ONOO^-,CO_2}$) of $2.9 \times 10^4$ $M^{-1}s^{-1}$ at physiological levels to create a strong oxidizing adduct, $ONOOCO_2^-$. The acid form of this compound, peroxynitrous acid (ONOOH), does not exhibit reactivity with bicarbonate ($HCO_3^-$) or $CO_2$. Nevertheless, it was not clear whether the concentration of dissolved $CO_2$, which comes from the equilibrium with air in the water/buffer systems, is sufficient to significantly impact peroxynitrite

degradation in these experiments. To explore this, the role of varying bicarbonate concentrations was examined.

Apparent first-order decay rate constants of peroxynitrite were compared in presence of bicarbonate to simulate varying $CO_2$ concentrations. Sodium bicarbonate was added to phosphate buffers (70 mM) at pH 8.2 and 9 in concentrations ranging from 0.05 to 50 mM, and the peroxynitrite decay was monitored as described in Section S2. The pH of the reaction mixture was measured, and each bicarbonate level was tested in triplicate. Various $CO_2$ concentrations were achieved in equilibrium with bicarbonate (Table S5). The carbonate and peroxynitrite speciation, illustrated in Figure S3, was accounted for in the analysis. Apparent first-order decay rate constants ($k_{obs}$) were determined for these experiments.

**Table S2** – Calculated concentrations of $H_2CO_3^*$ (or dissolved $CO_2$) at pH 8.2 for various initial bicarbonate concentrations. The applied equilibrium constants were $K_1 = 10^{-6.3}$ M and $K_2 = 10^{-10.3}$ M for the dissociation of carbonic acid and bicarbonate, respectively.[7]

| $NaHCO_3$ (mM) | $H_2CO_3^*$ (mM) |
|---|---|
| $5.00 \times 10^{1}$ | $6.20 \times 10^{-1}$ |
| 5.00 | $6.20 \times 10^{-2}$ |
| $5.00 \times 10^{-1}$ | $6.20 \times 10^{-3}$ |
| $5.00 \times 10^{-2}$ | $6.20 \times 10^{-4}$ |

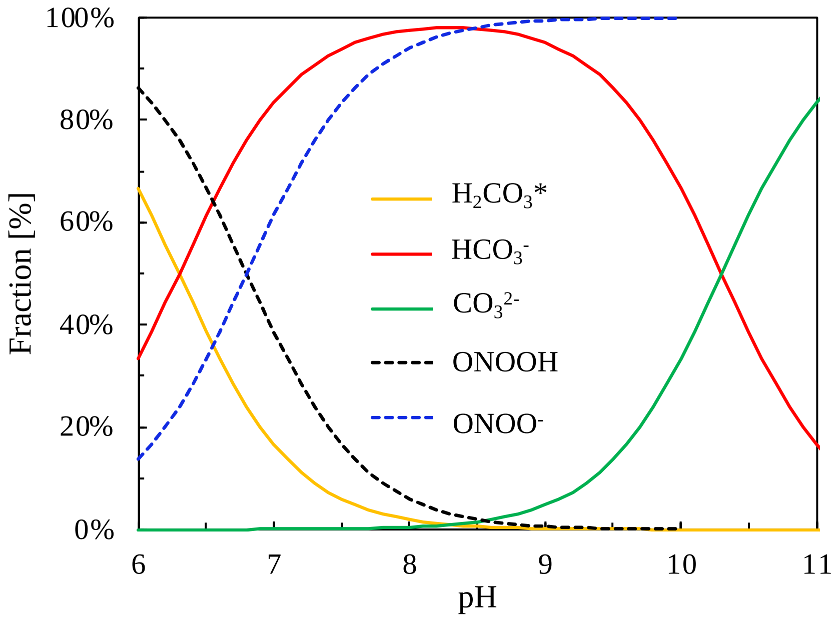


**Figure S3** – pH-dependent speciation of carbonate and peroxynitrite.

Table S6 shows the apparent first-order decay rate constants and the resulting half-life times for peroxynitrite at different bicarbonate levels at pH 8.2. For low tested bicarbonate concentrations (0.5 and 0.05 mM), no significant increase in the first-order decay rate constants was observed. When the $HCO_3^-$ concentration reached 50 mM, peroxynitrite decayed immediately, making it impossible to

calculate $k_{obs}$. At a lower $HCO_3^-$ concentration of 5 mM, the peroxynitrite degradation was notably accelerated, with a half-life of 4.1 s, approximately four times lower than for the control (16.3 s). In this case, the added 5 mM of $HCO_3^-$ at pH 8.2 represented an eight-fold excess over peroxynitrite in terms of dissolved $CO_2$. Such conditions, with a pH between 7.8 and 8.2 and $CO_2$ (aq) concentrations corresponding to an alkalinity of 3 mM, are typical for secondary wastewater effluents. This implies that under typical wastewater treatment conditions, dissolved $CO_2$ could play a crucial role in affecting peroxynitrite stability.

**Table S6** – Measured apparent first-order rate constants with SDs for the decay of peroxynitrite (0.5 mM) at pH 8.2 in presence of varying concentrations of $HCO_3^-$ in a 70 mM phosphate buffer, along with associated half-life times($t_{1/2}$). The value provided for 0 mM $HCO_3^-$ is taken from Section S2.1.

| $[NaHCO_3]$ (mM) | $k_{obs}$ ($s^{-1}$) | σ ($s^{-1}$) | $t_1/_2$ (s) |
|---|---|---|---|
| 0 | $4.26 \times 10^{-2}$ | $1.14 \times 10^{-3}$ | 16.3 |
| 0.05 | $3.94 \times 10^{-2}$ | $2.67 \times 10^{-3}$ | 17.6 |
| 0.5 | $4.89 \times 10^{-2}$ | $4.19 \times 10^{-3}$ | 14.2 |
| 5 | $1.68 \times 10^{-1}$ | $3.94 \times 10^{-2}$ | 4.1 |

## S2.3 Ionic strength

The ionic strength has been reported to influence the $pK_a$ which in turn influences the stability of peroxynitrite since the conjugated acid is very unstable.[6] To investigate the impact of ionic strength on peroxynitrite stability, experiments were conducted for varying ionic strengths. The experimental procedure was the same as described in Section S2.1 with a different phosphate buffer pH 7.8 and I = 0.21 M so that upon addition of the alkaline peroxynitrite stock solution (0.3 mL in NaOH 25 mM) the resulting pH was 8. Moreover, three sub-samples of this buffer were spiked with increasing amounts of NaCl yielding different ionic strengths of 0.23 M, 0.26 M and 0.32 M. After the addition of the peroxynitrite stock solution to each buffer sub-sample, the calculated ionic strengths were 0.19, 0.21, 0.24 and 0.29 M. The ionic strength of the samples was calculated as follows (eq. S2)[8].

$$I = \frac{1}{2} . \sum_{i=0}^{n} c_i . z_i^2 \qquad \text{(S2)}$$

where $c_i$ corresponds to the concentration of the ion and $z_i$ to its charge.

Measurements were taken in triplicates and the pH of each set of triplicates was measured at the end. The solutions were mixed at room temperature and the peroxynitrite decay was monitored and the apparent first-order decay rate constants were determined.

The apparent first-order rate constants for peroxynitrite decay as a function of the ionic strength are shown in Figure S4, and the corresponding half-life times are provided in Table S7. None of the ionic

strengths tested showed a significant effect on the apparent first-order decay rate constant for peroxynitrite. Yet, at an ionic strength of 0.29 M, which is a 0.1 M increase from the control, there was a slight effect. At this ionic strength, the first-order rate constant was marginally reduced ((3.90± 0.05) × $10^{-2}$ $s^{-1}$) in comparison to the control at 0.19 M ((4.27± 0.19) × $10^{-2}$ $s^{-1}$). These results align with findings of Molina et al. 2013,[6] who suggested that peroxynitrite becomes more stable at higher ionic strengths. Nevertheless, the difference was minimal for the 0.1 M change in ionic strength, with the half-life only slightly decreasing from 17.8 s to 16.2 s. Therefore, peroxynitrite decay is not significantly impacted if the change in ionic strength is $\leq$ 0.1 M.

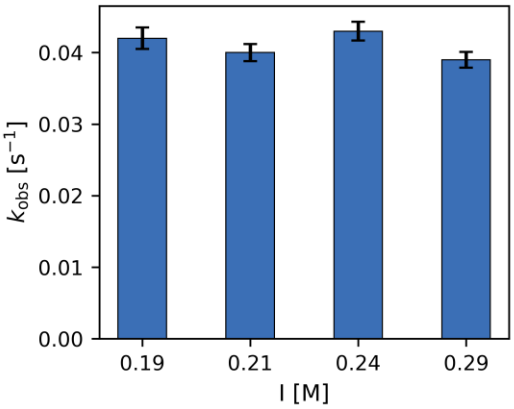


**Figure S4** – Measured apparent first-order decay rate constants for 0.5 mM peroxynitrite in a 70 mM phosphate buffer at pH 8, with variable ionic strengths achieved by addition of NaCl.

**Table S7** – Apparent first-order decay rate constants with SDs of peroxynitrite (0.5 mM) at pH 8 and different ionic strengths (I) and the corresponding half-life times (same data as in Figure S4).

| **I (M)** | **$k_{obs}$ ($s^{-1}$)** | **σ ($s^{-1}$)** | **$t_{1/2}$ (s)** |
|---|---|---|---|
| 0.19 | 4.27 × $10^{-2}$ | 1.09 × $10^{-3}$ | 16.2 |
| 0.21 | 3.09 × $10^{-2}$ | 1.12 × $10^{-3}$ | 17.4 |
| 0.24 | 4.25 × $10^{-2}$ | 4.53 × $10^{-4}$ | 16.3 |
| 0.29 | 3.90 × $10^{-2}$ | 4.91 × $10^{-4}$ | 17.8 |

## S2.4 Influence of nitrite

The decay of peroxynitrite at different nitrite concentrations was investigated at pH 7, 8 and 9 in phosphate buffer (70 mM). At each pH, peroxynitrite-containing solutions (0.5 mM) were spiked with increasing nitrite concentrations (0, 0.5, 5, and 20 mM). Additionally, to simulate ozonation conditions where nitrite is oxidized to nitrate, the decay of 50 µM peroxynitrite in the presence of 2 mM nitrate and 18 mM nitrite was monitored at each pH. The pH of each set of triplicates was checked after spiking and measurements were performed to ensure that it did not deviate from the expected

values (within ± 0.1 units). UV-VIS absorbance was measured in a 5-cm cuvette and the apparent first-order decay rate constants were determined. For samples containing > 0.5 mM nitrite, blanks (buffer and nitrite at different concentrations) were prepared and the measured absorbance at 302 nm was subtracted from the absorbance of the corresponding samples.

Peroxynitrous acid can oxidize nitrite yielding nitrate.[9] This reaction has been reported to be rather slow and of limited influence since even when nitrite was present at a concentration 500 times higher than peroxynitrous acid, the increase in the observed first-order decay rate constant ($k_{obs}$) was insignificant, rising from (1.12 ± 0.07) $s^{-1}$ to (1.25 ± 0.10) $s^{-1}$ at pH 5.2 according to a study of Maurer et al., 2003.[9]

In the present study, peroxynitrite decay was monitored in the presence of various nitrite concentrations. Figure S5 presents the average first-order decay rate constants for peroxynitrite at pH 7, 8, and 9 for different nitrite concentrations. The decay of peroxynitrite was faster at 20 mM nitrite, a 40-fold excess over peroxynitrite, except at pH 7. Here, despite an increase in the mean apparent first-order decay rate constant, no significant difference from the control was observed, potentially due to the relatively high standard deviations at pH 7 (± 7 - 12%). The observed increase in the apparent first-order decay rate constant at pH 8 (+14%) and particularly at pH 9 (+27%) exceeds the +12% relative change reported by Maurer et al. 2003[9] for a 500-fold excess of nitrite over peroxynitrite at pH 5.2. The effect was even more pronounced at pH 9 compared to pH 8, and at 5 mM nitrite, a noticeable enhancement in the apparent first-order decay rate constant was observed only at pH 9. In summary, a more substantial degradation of peroxynitrite occurs at pH 8 and 9 with a 40-fold excess of nitrite, especially at pH 9, where even a 10-fold excess leads to enhanced decomposition compared to the control. These findings suggest interactions between nitrite and peroxynitrite beyond the oxidation of nitrite by peroxynitrous acid. Nevertheless, during ozonation, such relatively small differences in peroxynitrite stability do not seem to be relevant.

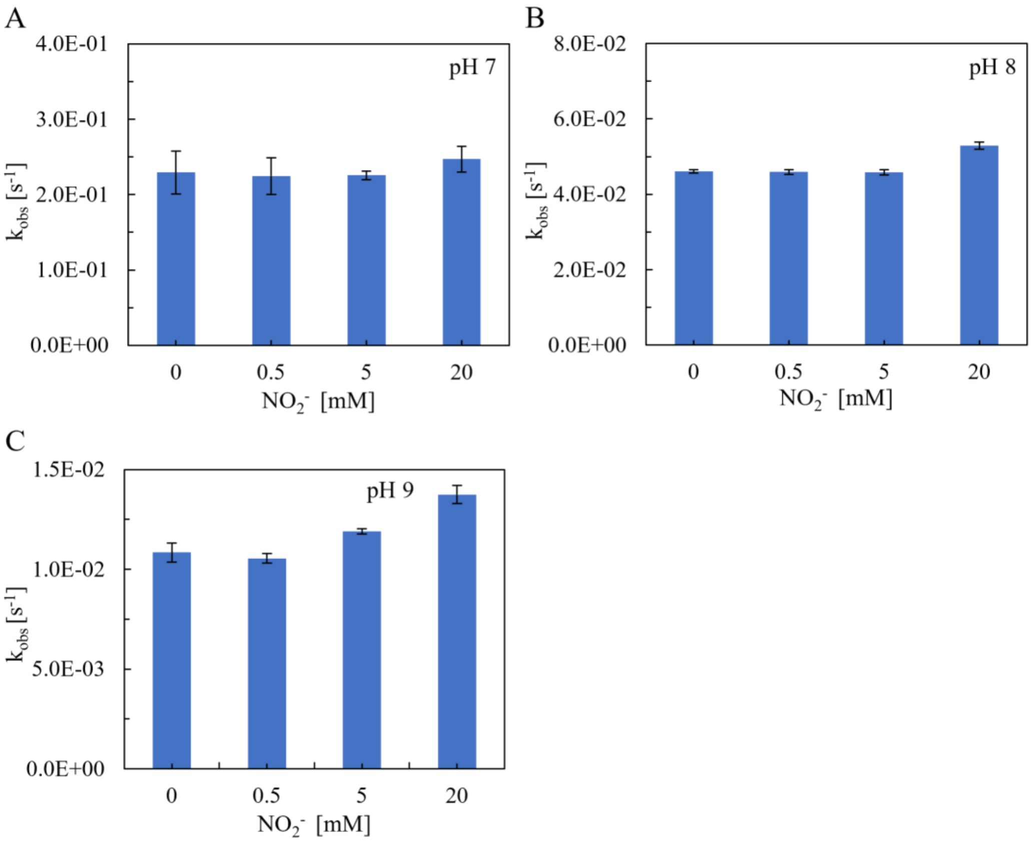


**Figure S5** – Measured apparent first-order decay rate constants for 0.5 mM peroxynitrite at (A) pH 7, (B) pH 8, and (C) pH 9 in a 70 mM phosphate buffer, with an ionic strength of approximately 0.2 M, at various nitrite concentrations.

# S3 Assessment of peroxynitrite formation during ozonation of nitrite-containing solutions

## S3.1 Testing of peroxynitrite formation by direct measurement

Naumov et al. 2010 reported that the ozonation of an alkaline solution (pH = 11) containing 2.5 mM nitrite resulted in a 2.6% yield of peroxynitrite, identified by an increase in absorption at 302 nm observed in a 5-cm quartz cuvette.[10]

An experiment in this study aimed to determine peroxynitrite formation during nitrite ozonation at various pH, including those typical for secondary wastewater effluent. For this purpose, 10 mM nitrite solutions in sodium hydroxide (0.01 mM at pH 9 and 1 mM at pH 11) were dosed with ozone doses of 200 to 800 µM. During the process, the solution was stirred gently, and ozone was added gradually. Upon the addition of ozone, the resulting mixture was transferred to a 10-cm quartz cuvette for immediate absorbance measurement at 302 nm to detect peroxynitrite. Additionally, absorbance readings for a blank solution containing 10 mM nitrite and a control solution with 10 mM nitrite with approximately 10 µM peroxynitrite were recorded at each pH level. The control absorbance was continuously monitored at 302 nm for a duration of 2 to 10 min. All measurements were conducted in duplicate, and the pH of each solution was subsequently recorded. The observed absorbance was compared to two calculated absorbance values. The first calculation was based on the assumption that no peroxynitrite formed, considering only the oxidation of nitrite to nitrate. The second calculation took into account the potential formation of peroxynitrite with yields ranging from 0.5 to 2%.

To ensure that hydroxyl radicals ($^{\bullet}OH$) did not affect the experiments, additional control experiments were conducted using *t*-BuOH or DMSO as $^{\bullet}OH$ scavengers. These scavengers were added in large excess, ensuring a quenching rate for $^{\bullet}OH$ at least 20-fold higher than the reaction with nitrite. Control experiments were also conducted to assess any direct reactivity between peroxynitrite and the scavengers, as well as to check for their potential co-absorbance at 302 nm.

The experimental data generated in the absence of $^{\bullet}OH$ scavengers are shown in Figure S6. No formation of peroxynitrite was observed at pH 9 and 11, with a detection limit of 1 µM for peroxynitrite. In the ozonated samples, all measured absorbance levels were below the control sample, which had been spiked with approximately 10 µM peroxynitrite simulating a yield of about 1.5% for the highest applied ozone dose of approximately 700 µM, and below the absorbance expected in case peroxynitrite was generated at a yield of 0.5% to 2%. The absorbance values were quite similar to those predicted under the assumption that all nitrite was converted to nitrate. A minor decreasing trend in the measured absorbance with increasing ozone concentrations was noted, suggesting a

greater conversion of nitrite to nitrate, which absorbs less at 302 nm compared to nitrite. This observation is in line with the predicted outcomes assuming oxidation of nitrite to nitrate without a significant formation of peroxynitrite. Overall, peroxynitrite formation during ozonation of nitrite was not detected, in contrast to Naumov et al. 2010 [10] under similar experimental conditions.

For a better understanding of the reaction system, the reactivity of ozone with peroxynitrite and the $^{\bullet}OH$ yield from ozone nitrite reaction were determined. The results of these experiments are presented in Section S3.2.

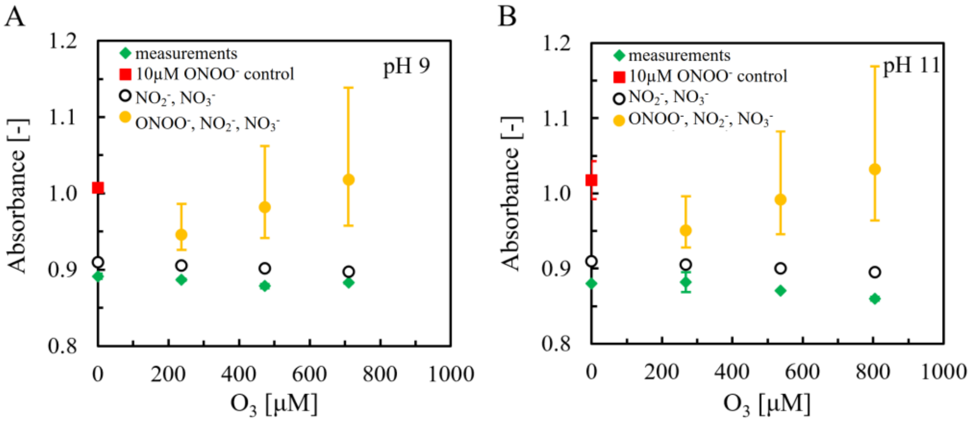


**Figure S6** – Absorbance results of ozonation of 10 mM nitrite as a function of the ozone dose in solutions of (A) 10 µM NaOH at pH 9 and (B) 1 mM NaOH at pH 11, measured in a 10-cm quartz cuvette at 320 nm. The presented data includes absorbance of the experimental samples (green diamonds) and a control sample containing 10 µM peroxynitrite and 10 mM nitrite (red square). The predicted absorbance values are also shown for the assumption that all nitrite is converted to nitrate (black circles), as well as the expected absorbance if peroxynitrite is formed at a 1% yield (yellow circles). Additionally, the anticipated absorbance range for peroxynitrite yields fluctuating between 0.5% (lower ends of yellow bars) and 2% (upper ends of yellow bars) is shown.

Additional experiments were performed in the presence of a large excess of *t*-BuOH or DMSO to rule out potential reactions between $^{\bullet}OH$ and peroxynitrite (Figure S7). Furthermore, the concentration of nitrite was reduced from 10 mM to 1 mM to lower the ratio of nitrite to peroxynitrite, to exclude the slightly increased degradation of peroxynitrite in presence of high nitrite concentrations (see Section 2.4)

The findings, shown in Figure S7, are similar to those in absence of hydroxyl radical scavengers; no increase in absorbance was noted at either pH 9 or 11 in presence of *t*-BuOH or DMSO. Instead, absorbance values diminished as ozone doses increased.

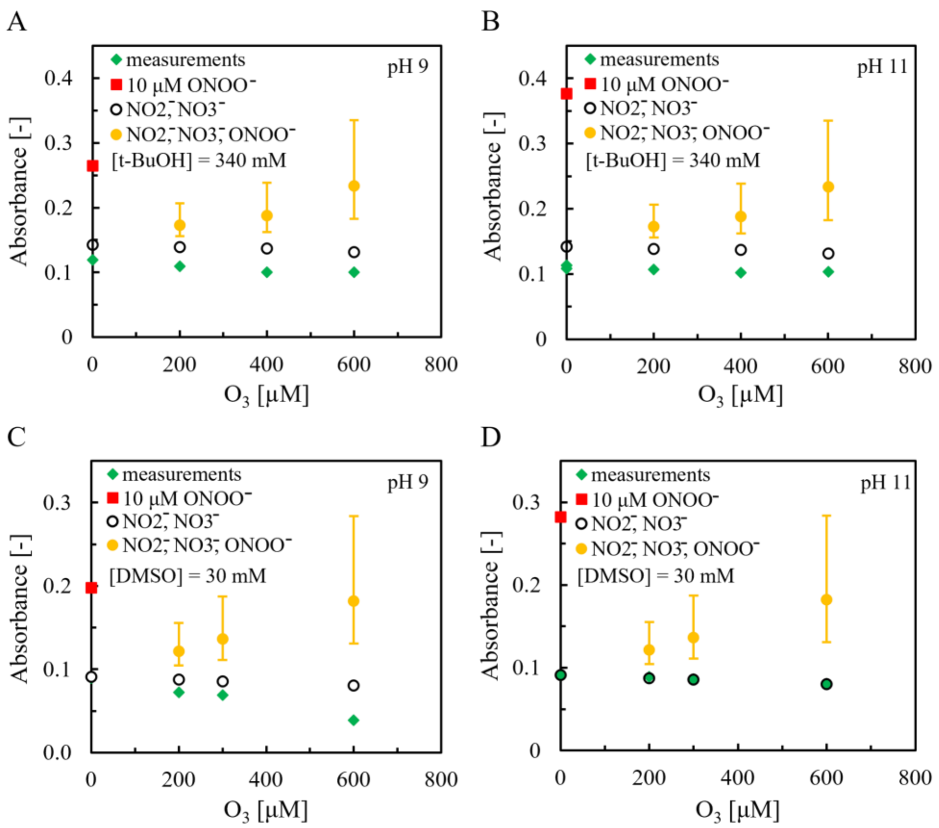


**Figure S7** – Absorbance in a 10-cm quartz cuvette at 320 nm as function of the ozone dose for the ozonation of 1 mM nitrite in presence of 420 mM *t*-BuOH at (A) pH 9 and (B) pH 11 or 30 mM DMSO at (C) pH 9 and (D) pH 11 (for experimental conditions see Figure S6). The data show absorbance of the measured samples (green diamonds) and a control containing 10 µM peroxynitrite (red square), calculated absorbance if nitrite is completely oxidized to nitrate (black circles), calculated absorbance assuming peroxynitrite is formed at a yield of 1 % (yellow circles), and calculated absorbance range assuming peroxynitrite yields varying from 0.5 % (yellow bars, lower end) to 2 % (yellow bars, higher end). For the calculated absorbance values in samples with *t*-BuOH, the absorbance was corrected by subtracting the absorbance of *t*-BuOH (measured as 0.05 at 320 nm based on a blank containing only *t*-BuOH).

## S3.2 Determination of the second-order rate constant of the reaction of ozone with peroxynitrite

The potential reaction between peroxynitrite and ozone was studied to explore the fate of peroxynitrite during ozonation of nitrite in aqueous solutions. Pryor et al. 1995[11] suggested that the second-order rate constant of the ozone reaction with peroxynitrite is comparable to that of ozone reaction with the hydroperoxide anion ($5.5\times10^{6}$ $M^{-1}$ $s^{-1}$ [11]). Accordingly, cinnamic acid was chosen as an appropriate competitor candidate with $7.6\times10^{6}$ $M^{-1}$ $s^{-1}$ at T = 22 °C for the anionic form.[12] Control experiments confirmed the absence of the reaction between cinnamic acid and peroxynitrite and allowed the selection of an appropriate alkaline pH condition (pH 9) where peroxynitrite did not degrade quickly and benzaldehyde formation was equimolar to cinnamic acid disappearance.

Two different sets of solutions containing different concentrations of cinnamic acid (0.1 – 2 mM) and an excess of *t*-BuOH (600 mM) in phosphate buffer (pH 8.5 or 9) were prepared and sparged with

argon. To one set in phosphate buffer pH 9, ozone was dosed at 45 µM while mixing with a magnetic stirrer. The second set in phosphate buffer (pH 8.5) was spiked with 0.1 mM peroxynitrite (in NaOH 10 mM) resulting in a solution with a final pH of 9 to which ozone was dosed subsequently at 45 µM while mixing with a magnetic stirrer. The vials were closed with caps and left on the magnetic stirrer for about 10 min. All solutions were prepared in triplicates. From each sample, a 100 µL aliquot was diluted with 900 µL ultra-purified water and analyzed by HPLC-DAD to quantify the disappearance of cinnamic acid and the formation of benzaldehyde during ozonation.

Results of the competition kinetics experiment based on Munoz and von Sonntag 2000 [13] are shown in Figure S8. The second-order rate constant for the ozone-peroxynitrite reaction was deduced from the concentration of the formed benzaldehyde, with and without peroxynitrite. A linear regression of the relative benzaldehyde formation as a function of the molar ratio of peroxynitrite:cinnamic acid allowed the determination of the second-order rate constant. Using the second-order rate constant for the reaction of ozone with cinnamic acid ($7.6\times10^{6}$ $M^{-1}$ $s^{-1}$) determined in Wolf et al. 2018 [12] at 22 °C, the second-order rate constant for the reaction of peroxynitrite with ozone was determined as $(4.96 \pm 0.40) \times 10^{6}$ $M^{-1}$ $s^{-1}$ which is close to the suggested value for the hydroperoxide anion (see above).

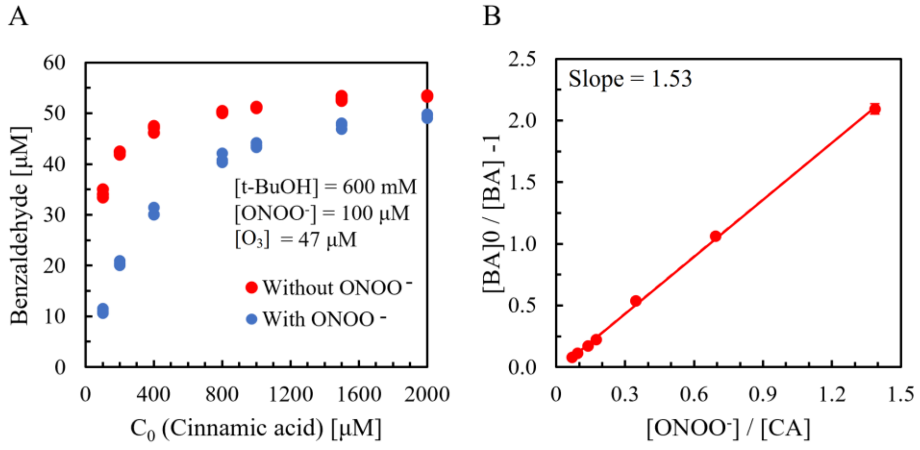


**Figure S8** – Determination of the second-order rate constant at pH 9 for the reaction of ozone with peroxynitrite by competition kinetics with cinnamic acid. (A) Observed formation of benzaldehyde upon ozonation of different concentrations of cinnamic acid with 47 µM ozone and 100 µM peroxynitrite. (B) Competition kinetics plot in which the slope corresponds to the ratio of the second-order rate constant of the two competitors (here peroxynitrite and ozone, slope=1.53±0.015) according to von Sonntag and von Gunten 2012[3] and Munoz and von Sonntag 2000.[13]

# S4 Hydroxyl radical yield

## S4.1 Determination of hydroxyl radical yields during ozonation of nitrite

The yields of hydroxyl radicals for the reaction of ozone with nitrite were measured in the pH range 7 -12 by ozonation of nitrite in the presence of excess *t*-BuOH and measuring the subsequent production of formaldehyde according to Flyunt et al. 2003.[14] In addition, concentrations of nitrite and nitrate were measured (Section **Error! Reference source not found.**) to complete the mass balance of the system.

Solutions containing nitrite (390 µM) and *t*-BuOH (200 mM) in phosphate-buffered solutions (10 mM) at various pH levels were treated with different doses of ozone (up to approximately 350 µM). During and after ozone addition, solutions were stirred for 5 minutes, after which the final pH was verified, and measurements were taken in triplicates.

Formaldehyde was detected by the Hantzsch reaction.[14] To prepare the Hantzsch reagent, 100 g of ammonium acetate was dissolved with 20 mL acetic acid and 0.8 mL acetylacetone in ultra-purified water, bringing the total volume to 400 mL. Then, 5 mL of the sample was mixed with 2 mL of the Hantzsch reagent, heated at 50 °C for 30 min, and the absorbance was recorded at 412 nm using 5-cm narrowed-quartz cuvettes (smaller volume required compared to a regular 5-cm cuvette). An external calibration curve was used for formaldehyde quantification. The formaldehyde yield was calculated by linear regression as a function of the ozone dose (Table S8). The $^{\bullet}OH$ yield (Table S9) was estimated to be twice the formaldehyde yield and its pH-dependence is also shown in Figure 1 in the main text.[14]

**Table S7** – Apparent first-order decay rate constants with SDs of peroxynitrite (0.5 mM) at pH 8 and different ionic strengths (I) and the corresponding half-life times (same data as in Figure S4).

| pH | Slope | S.D. Slope | Intercept | S.D. Intercept | $R^2$ |
|---|---|---|---|---|---|
| 7 | $4.72 \times 10^{-2}$ | $1.65 \times 10^{-3}$ | $-3.86 \times 10^{-1}$ | $2.89 \times 10^{-1}$ | 0.977 |
| 8 | $5.37 \times 10^{-2}$ | $1.28 \times 10^{-3}$ | $-1.70 \times 10^{-1}$ | $2.64 \times 10^{-1}$ | 0.989 |
| 9 | $5.93 \times 10^{-2}$ | $2.55 \times 10^{-3}$ | $-8.01 \times 10^{-2}$ | $3.70 \times 10^{-1}$ | 0.966 |
| 10 | $9.20 \times 10^{-2}$ | $4.68 \times 10^{-3}$ | $-9.96 \times 10^{-1}$ | $5.74 \times 10^{-1}$ | 0.967 |
| 11 | $1.07 \times 10^{-1}$ | $5.09 \times 10^{-3}$ | $-2.24 \times 10^{0}$ | $7.94 \times 10^{-1}$ | 0.959 |
| 12 | $1.11 \times 10^{-1}$ | $2.66 \times 10^{-3}$ | $-1.80 \times 10^{0}$ | $5.50 \times 10^{-1}$ | 0.989 |

**Table S9** – Measured $^{\bullet}OH$ yields (based on consumed ozone) and standard deviation from the ozonation of nitrite in the pH range 7-12.

| pH | Yield | S.D. |
|---|---|---|
| 7 | 0.094 | $3.31 \times 10^{-3}$ |
| 8 | 0.107 | $2.56 \times 10^{-3}$ |
| 9 | 0.119 | $5.09 \times 10^{-3}$ |
| 10 | 0.184 | $9.37 \times 10^{-3}$ |
| 11 | 0.214 | $1.02 \times 10^{-2}$ |
| 12 | 0.221 | $5.32 \times 10^{-3}$ |

## S4.2 Hydroxyl radical yield from the decomposition of peroxynitrite

Different doses of peroxynitrite (0, 5, 25, 50, 75 and 100 μM) were spiked into phosphate buffer (10 mM) pH 7 and 8 containing a large excess of *t*-BuOH (30 mM). The formation of formaldehyde was determined as described in Section S4.1. $^{\bullet}OH$ yields formed during the decomposition of peroxynitrite are shown in Figure S9.

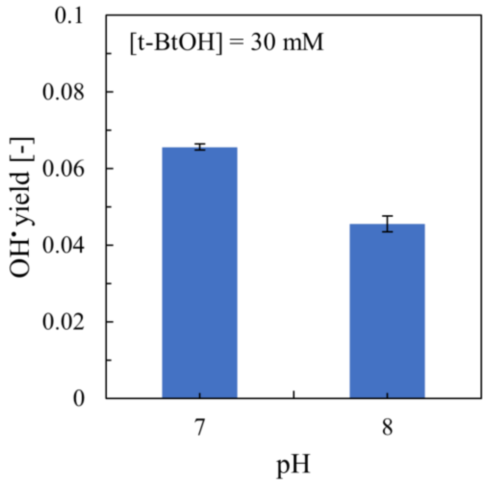


**Figure S9** – Observed hydroxyl radical yields for the decomposition of peroxynitrite in phosphate-buffered solutions at pH 7 and 8. Hydroxyl radical yields are based on consumed peroxynitrite.

## S5 Abatement of diuron and carbendazim during wastewater ozonation in presence of nitrite

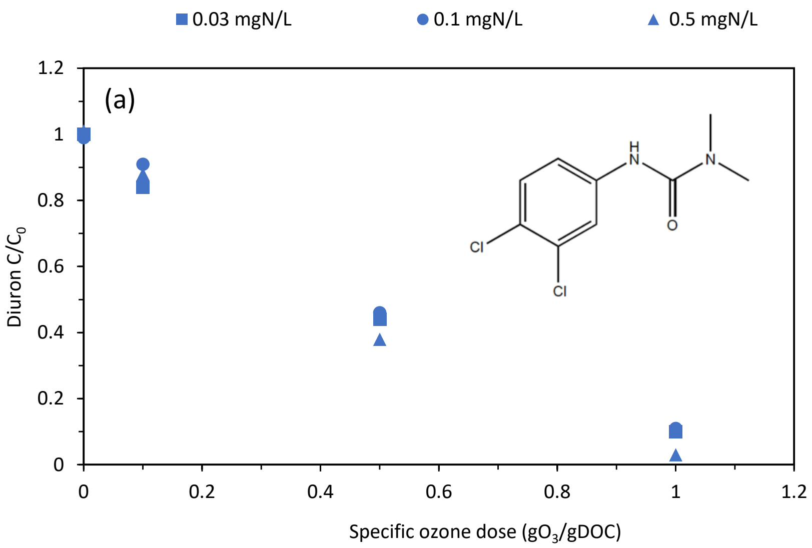


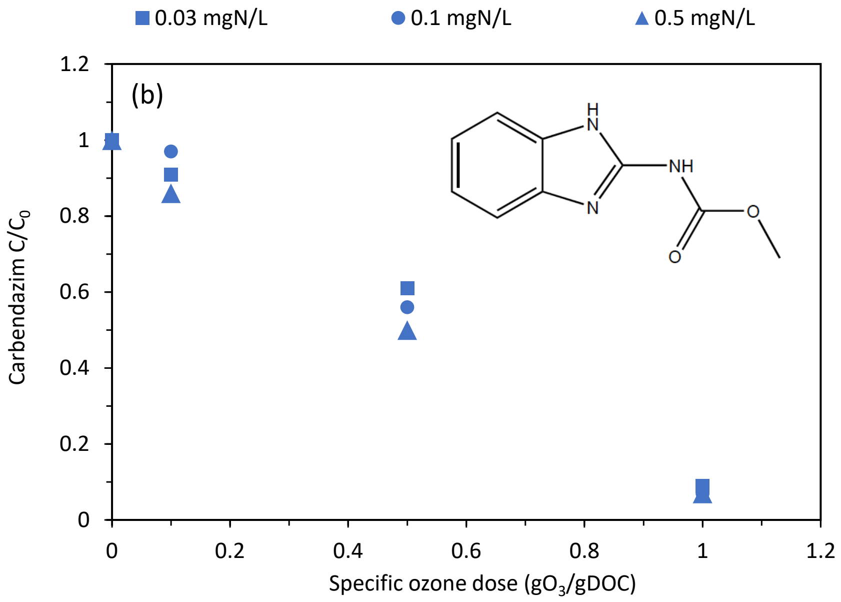


**Figure S10** – Relative residual concentrations of (a) diuron and (b) carbendazim during ozonation of secondary wastewater effluent as a function of the specific ozone dose at three different nitrite concentrations (0.03, 0.1 and 0.5 mgN/L). Conditions: secondary wastewater effluent (DOC: 6 mg/L, alkalinity 5.8 mmol/L, pH 8.3, Table S3), target compounds were spiked at a concentration of 1 µM.

# S6 Formation of nitrodiuron and nitrocarbendazim in the three investigated reaction systems

## S6.1 Diuron

### S6.1.1 $O_3/NO_2^-$

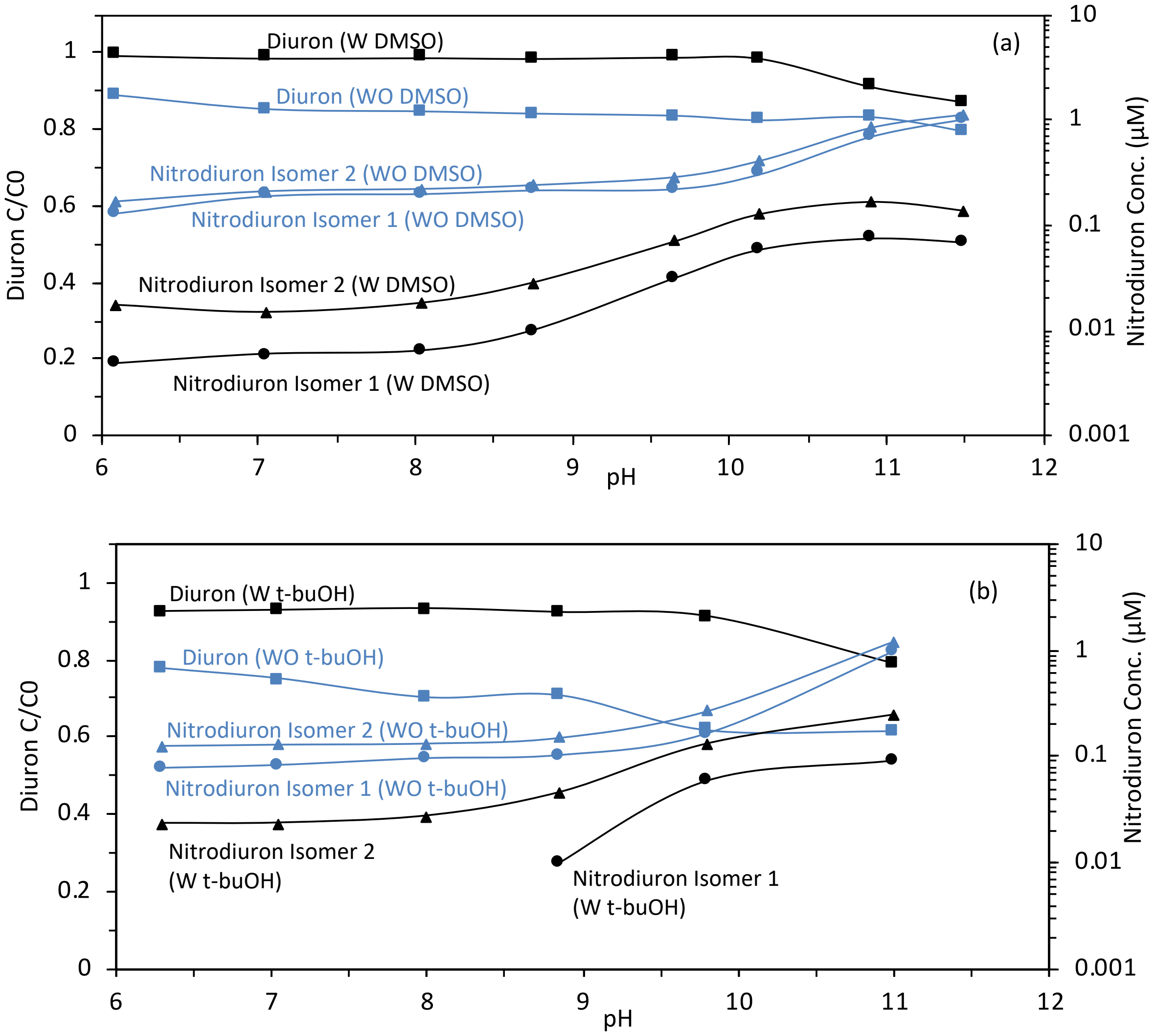


**Figure S11** – Ozonation of diuron. Relative residual concentration of diuron and logarithm of the formed nitro diuron (isomers-1 and -2, see Figure S1) concentrations during ozonation of nitrite-containing solutions as a function of the pH. (a) In presence of DMSO (W, black lines) and without DMSO (WO, blue lines): diuron 34 µM; phosphate buffer 10 mM, nitrite 0.50 mM, ozone 0.25 mM, DMSO 21.3 mM; calculated fraction of $^{\cdot}OH$ scavenged by DMSO (W), nitrite, and diuron is approximately 96.7%, 3.2% and 0.04%, respectively; in absence of DMSO (WO): 98.7% and 1.3% scavenged by nitrite and diuron, respectively. (b) In the presence of *t*-BuOH (W, black lines) and without *t*-BuOH (WO, blue lines): diuron 38.8 µM; phosphate buffer 10 mM, nitrite 0.5 mM, ozone 0.25 mM, *t*-BuOH 0 or 200 mM; calculated fraction of $^{\cdot}OH$ scavenged by *t*-BuOH (W) and nitrite is approximately 96% and 4% respectively; without *t*-BuOH (WO): calculated 98.5% and 1.5% of $^{\cdot}OH$ scavenged by nitrite and diuron, respectively. The lines are shown to guide the eye.

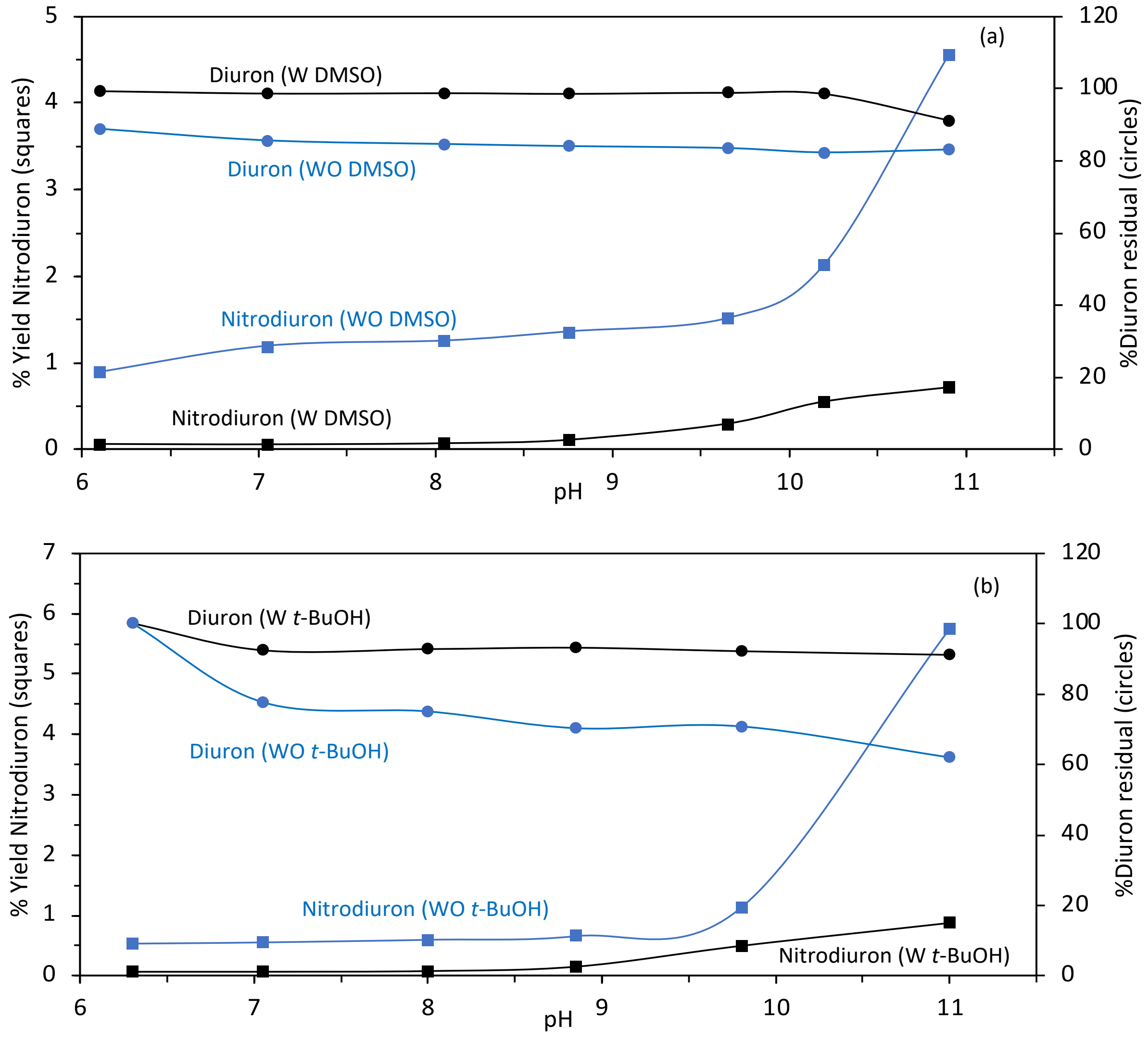


**Figure S12** – Ozonation of diuron. Yields of formation of nitrodiuron (blue symbols) (relative to the initial diuron concentration) and the relative residual diuron concentration as a function of the pH in the presence and absence of ·OH scavengers (a) DMSO, (b) *t*-BuOH). (a) Ozonation of nitrite-containing solutions containing diuron in the absence (blue circles/line) or presence (black circles/line) of DMSO and the corresponding formation of nitrodiuron (blue/black squares/lines); diuron 34 µM; phosphate buffer 10 mM, nitrite 0.50 mM, ozone 0.25 mM; DMSO 0 or 21.3 mM; calculated fraction of ·OH scavenged by DMSO and nitrite is approximately 97% and 3% respectively; without DMSO approximately 99%, and 1% by nitrite and diuron, respectively. (b) Ozonation of nitrite-containing solutions containing diuron in the absence (blue circles/line) or presence (black circles/line) of *t*-BuOH and the corresponding formation of nitrodiuron (blue/black squares/lines); diuron 38.8 µM; phosphate buffer 10 mM, nitrite 0.50 mM, ozone 0.25 mM; *t*-BuOH 0 or 200 mM; calculated fraction of ·OH scavenged by *t*-BuOH and nitrite is approximately 96% and 4% respectively; without *t*-BuOH: 98.5% and 1.5% ·OH scavenged by nitrite and diuron, respectively. The lines are shown to guide the eye.

## S6.1.2 γ-radiolysis/$NO_2^-$

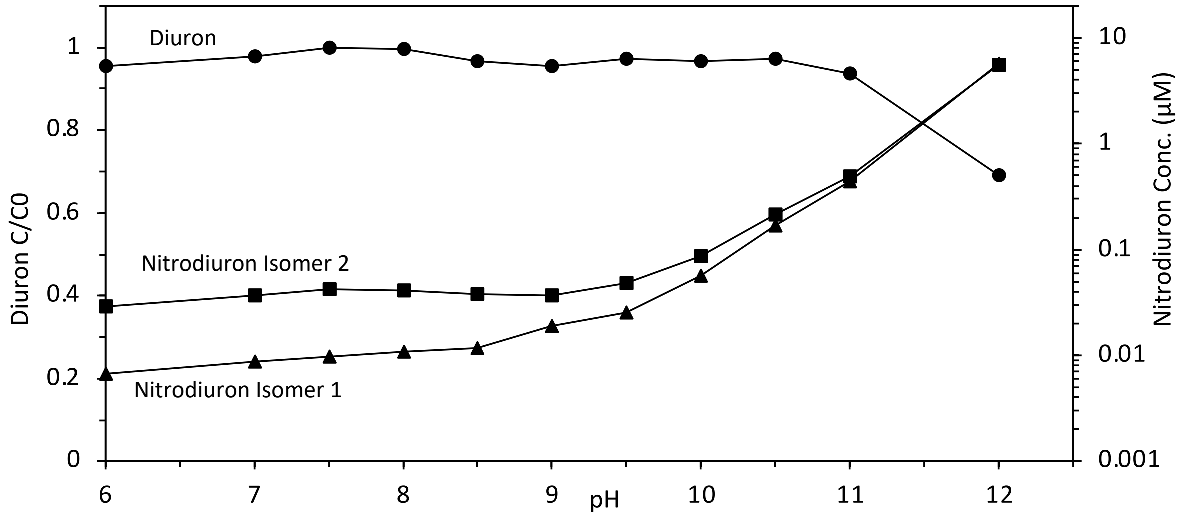


**Figure S13** – γ-Radiolysis experiments with diuron. Relative residual concentration of diuron and logarithm of the formed concentration of nitrodiuron isomers-1 and -2 (see Figure S1 for structures) as a function of the pH during γ-radiolysis of nitrite- and diuron-containing solutions; phosphate buffer 10 mM, diuron 42 µM, nitrite 2 mM, solutions with percentage saturation of 85:15% $N_2O$:$O_{2;}$ irradiation time 40 min. Estimated relative ·OH scavenging 99.6% and 0.4% by nitrite and diuron, respectively. All solutions were stored overnight at room temperature in the dark before analysis of diuron and the nitrodiuron isomers. Lines are shown to guide the eye.

## S6.1.3 Nitro-diuron yields in the three reaction systems

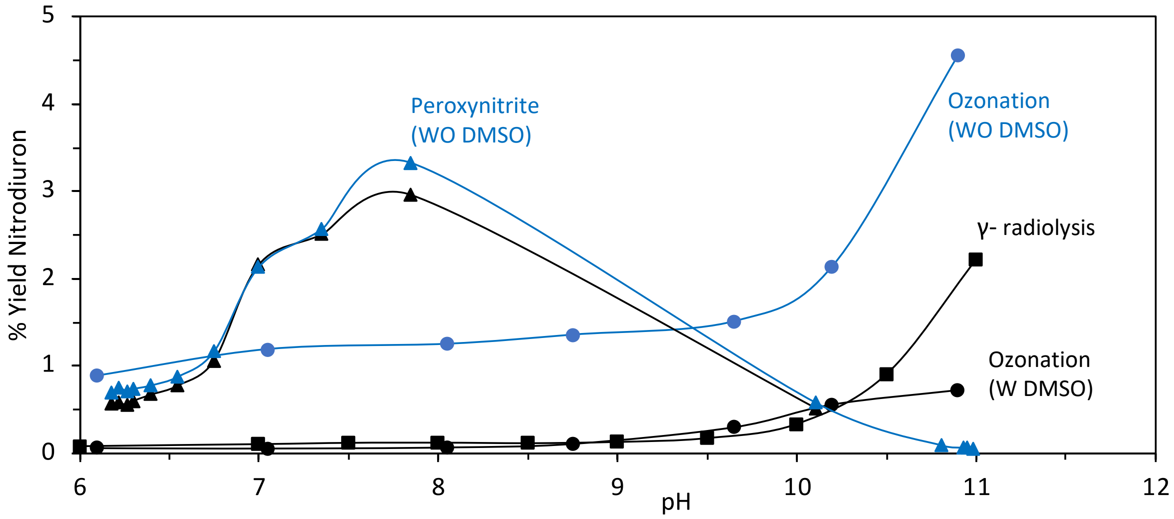


**Figure S14** – Formation of nitrodiuron (% molar yield relative to initial diuron concentration) in the three reaction systems (ozonation, peroxynitrite, γ-radiolysis) as a function of the pH. Ozonation of nitrite-containing solutions in the absence and presence of DMSO (blue and black circles, respectively); diuron 34 µM; phosphate buffer 10 mM, nitrite 0.5 mM, ozone dose 0.25 mM. Exposure to peroxynitrite in the absence and presence of DMSO (blue and black triangles, respectively); diuron 35.5 µM in phosphate buffer (50 mM), peroxynitrite 50 µM. γ-Radiolysis of nitrite- and diuron-containing solutions; phosphate buffer 10 mM, diuron 42 mM, nitrite 2 mM, solutions with $N_2O$:$O_2$ 85:15%; irradiation time: 40 min (black squares). All solutions were stored overnight at room temperature in the dark before analyses. The lines are shown to guide the eye.

## S6.2 Carbendazim

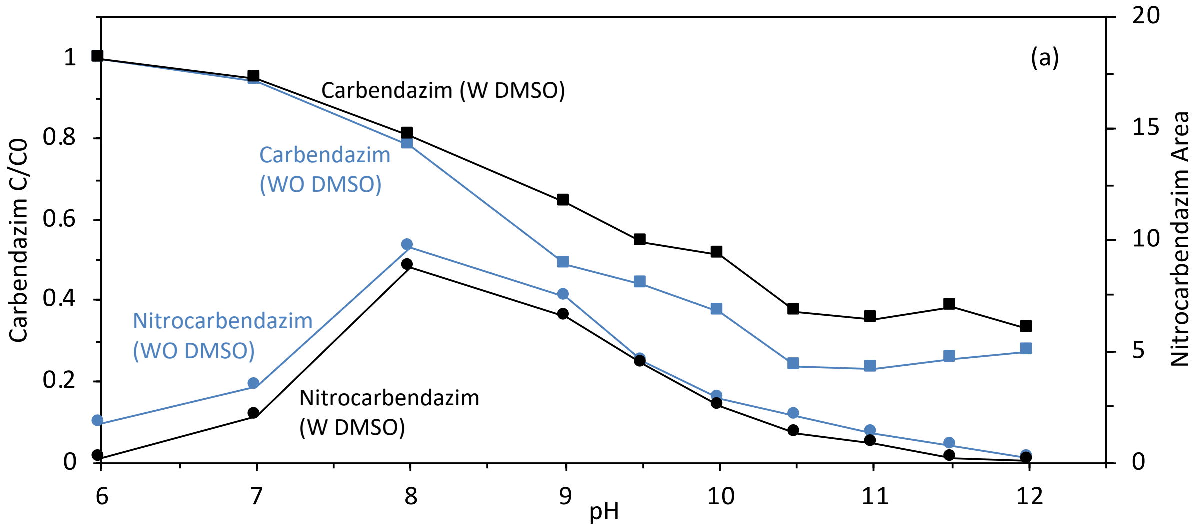


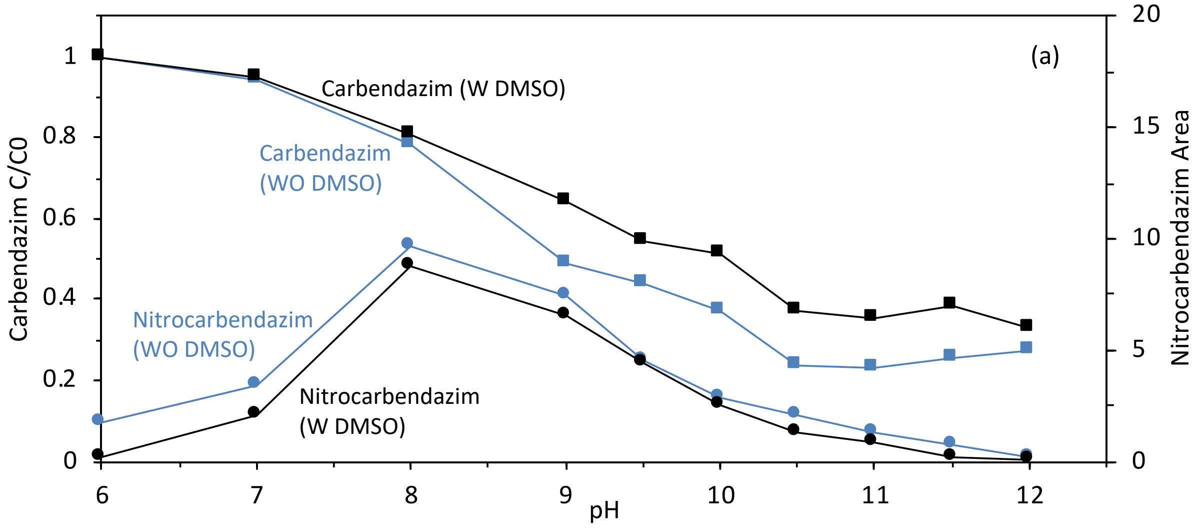


**Figure S15** – Ozonation of carbendazim. Relative residual concentrations of carbendazim and formation of nitrocarbendazim as a function of pH. (a) During ozonation of nitrite-containing solutions with DMSO (W, black symbols) and without DMSO (WO, blue symbols); carbendazim 26 µM; phosphate buffer 10 mM, nitrite 0.50 mM, ozone 0.25 mM, DMSO 21.3 mM (with DMSO); calculated fraction of $^{\cdot}$OH scavenged by DMSO, nitrite, and carbendazim is approximately 96.7, 3.2 and 0.03%, respectively; without DMSO: calculated scavenging 99% and 1% by nitrite and carbendazim, respectively. (b) Ozonation of nitrite-containing solutions with *t*-BuOH (black symbols) and without *t*-BuOH (blue symbols); carbendazim 26 µM; phosphate buffer 10 mM, nitrite 0.50 mM, ozone 0.25 mM, *t*-BuOH 0 or 200 mM; calculated fraction of $^{\cdot}$OH scavenged by *t*-BuOH and nitrite is approximately 96% and 4% respectively; without *t*-BuOH: 99% and 1% $^{\cdot}$OH scavenged by nitrite and carbendazim, respectively. The lines are shown to guide the eye.

# S7 Carbendazim byproducts

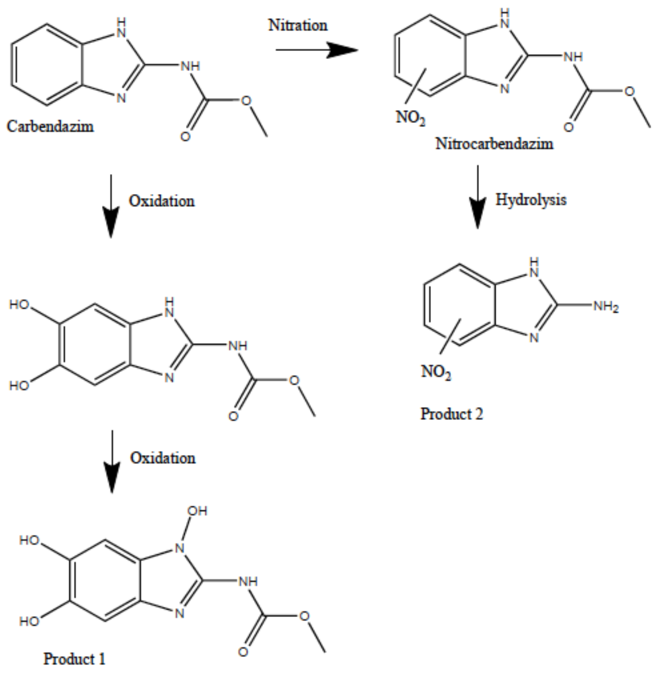


**Figure S16** – Carbendazim transformation scheme with structures of carbendazim nitration and oxidation products observed in γ-radiolysis of nitrite- and carbendazim-containing solutions (Figure 5c in main text).

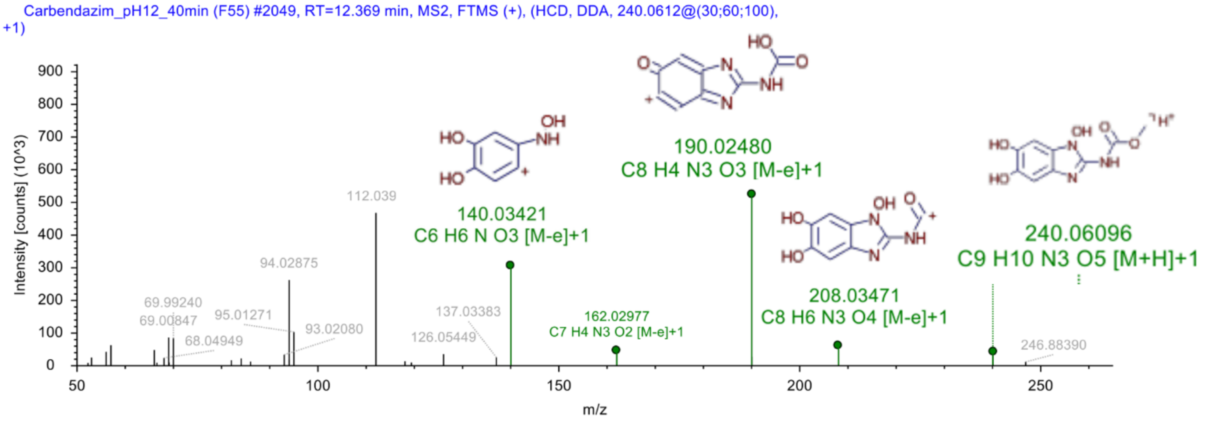


**Figure S17** – Annotated MS$^2$ spectrum of Carbendazim oxidation product (Product 1).

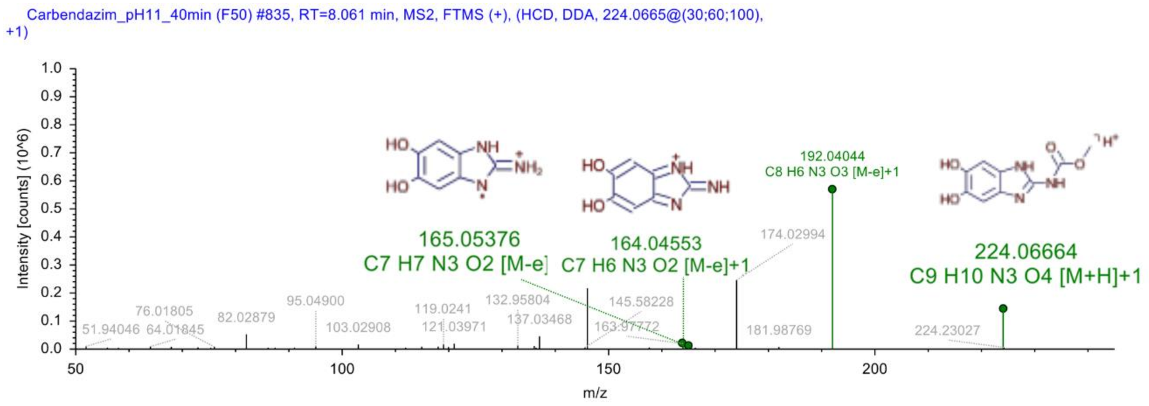


**Figure S18** – Annotated MS$^2$ spectrum of carbendazim intermediate oxidation product (intermediate *en route* to product 1).

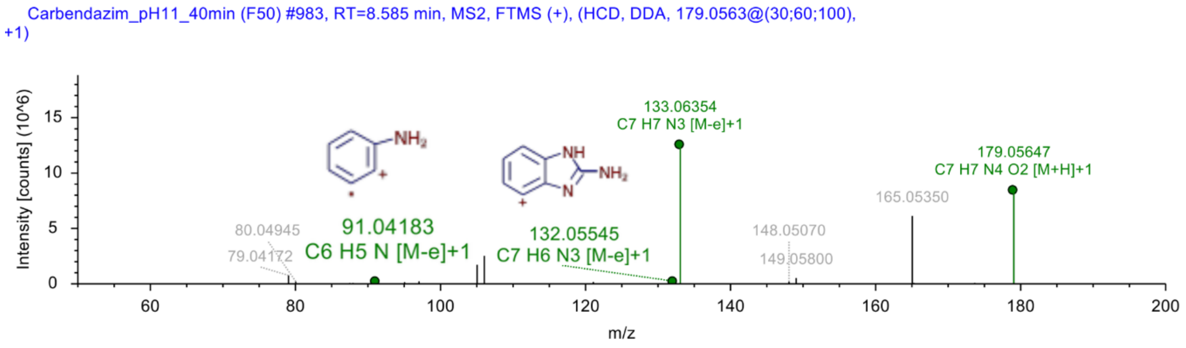


**Figure S19** – Annotated MS$^2$ spectrum of nitrocarbendazim hydrolysis product (product 2).

# S8 Determination of the second-order rate constant for the ozone reaction with carbendazim

The second-order rate constant for the reaction of ozone with carbendazim $k(O_3$, carbendazim) was determined by measuring the decrease of carbendazim under pseudo first-order conditions with a ten-fold excess of ozone (5 µM). A 250 mL glass bottle equipped with a bottle-top dispenser was used as a reaction vessel.[15] An ozone stock solution was added to the target compound solution, containing 0.5 µM carbendazim, 10 mM phosphate buffer (pH 7.00, 7.50 and 8.05) and 10 mM *t*-BuOH. Samples were withdrawn through the dispenser and quenched with cinnamic acid (min. 5-fold excess compared to ozone). The residual ozone concentration was determined by quantifying the reaction product of ozone with cinnamic acid, benzaldehyde, via HPLC (see above) and the decrease of carbendazim was also measured by HPLC (see Section S1.3.1). Second-order rate constants were obtained by plotting the relative natural logarithmic decrease of the target compound $\ln(c/c_0)$ as a function of the ozone exposure $\int[O_3]dt$. The apparent second-order rate constants can be calculated from the negative slope of the linear regression of such plots. The experiments were conducted in triplicates (experiment 4 at pH 7.5 was flagged as an outlier, see Figure S20 and Table S10). The linear regressions are shown in Figure S20 and the measured values are provided in Table S10.

**Table S10** – Measured apparent second-order rate constants for the reaction of carbendazim with ozone at varying pH values.

| Experiment | pH | $k_{app}$ | Coeff. of determination ($R^2$) |
|---|---|---|---|
| 1 | 7.00 | 5461 | 0.9992 |
| 2 | 7.01 | 4831 | 0.9976 |
| 3 | 7.00 | 5003 | 0.9961 |
| 4[a] | 7.49 | 8963 | 0.9980 |
| 5 | 7.50 | 6082 | 0.9925 |
| 6 | 7.51 | 5979 | 0.9949 |
| 7 | 8.05 | 11739 | 0.9955 |
| 8 | 8.05 | 11383 | 0.9982 |
| 9 | 8.07 | 10976 | 0.9957 |

[a] This experiment was marked as an outlier and has not been used for the linear regression.

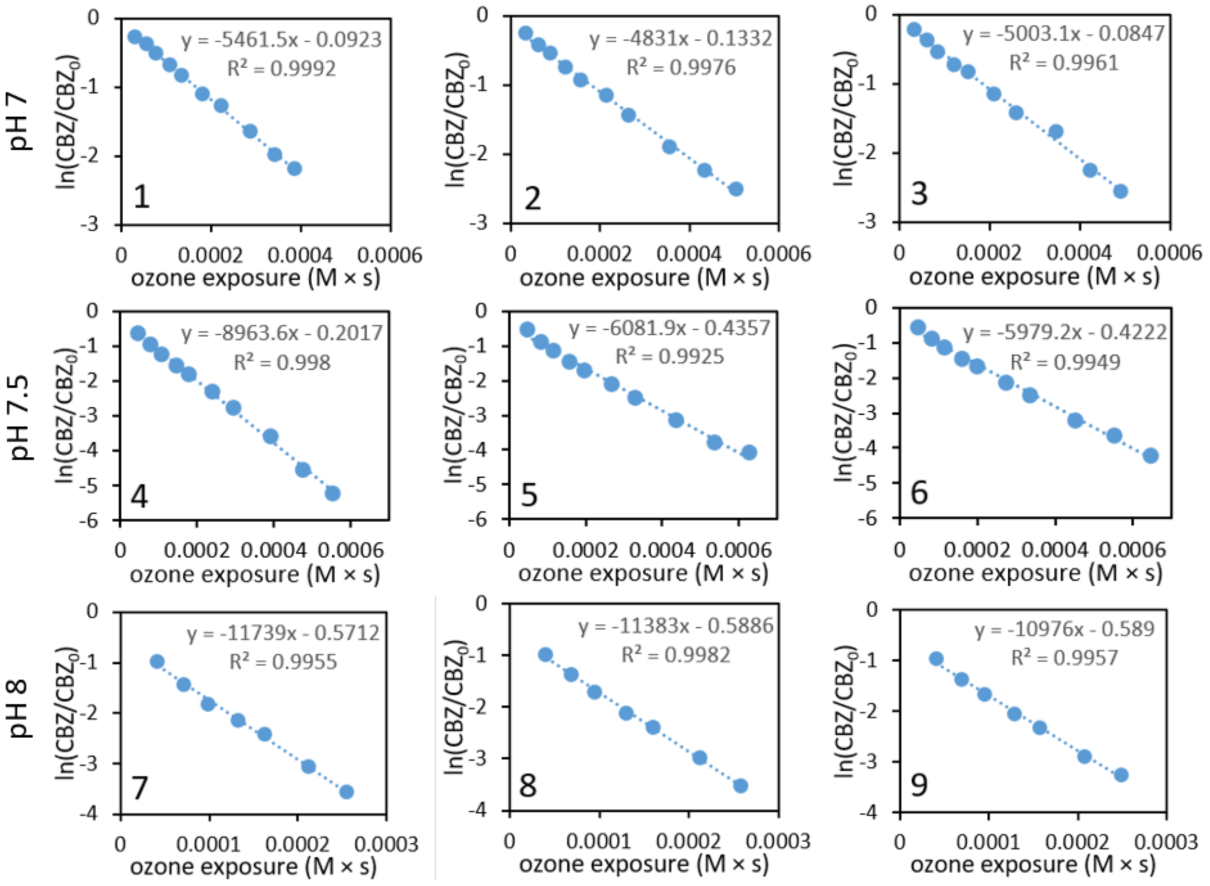


**Figure S20** – Linear regressions of the logarithmic relative residual concentration of carbendazim ln(CBZ/CBZ$_0$) as a function of the ozone exposure at pH 7 (Experiment 1-3), 7.5 (Experiment 4-6) and 8.05 (Experiment 7-9). Experiment 4 was marked as an outlier and has not been used for the calculation of the species-specific second-order rate constants.

The acid-base speciation of carbendazim is shown in Scheme 1. The lower p$K_a$ means that in the pH range of this study, the corresponding functional group will always be fully deprotonated and reacts much more slowly than the neutral form, which dominates a pH 5.5. This contrasts deprotonation, where the $k_{O_3}$ is 3 orders of magnitude higher, and thus the apparent $k_{O_3}$ already is influenced 3 pH units away from the p$K_a$. Therefore, any observed pH-dependence is due to the higher p$K_a$.

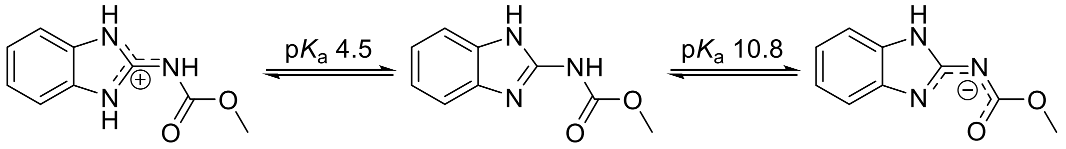


**Scheme S1** – Acid-base speciation of carbendazim.

The species-specific second-order rate constants were calculated by first calculating the fraction of the deprotonated form of carbendazim (p$K_a$ = 10.8±0.3) with the equation S3:

$$\%(\mathrm{deprotonated}) = \frac{10^{\mathrm{pH}-\mathrm{p}K_\mathrm{a}}}{10^{\mathrm{pH}-\mathrm{p}K_\mathrm{a}}+1} \qquad \text{(S3)}$$

p$K_a$ values for deprotonation of carbendazim were measured three times in the literature: Graham-Bryce et al. (1980)[16] determined a value of 10.9, Sancenon and de la Guardia (1994)[17] measured a value of 10.8±0.3, and Gauthier et al. (2000)[17] report 10.6±0.3. Because Graham-Bryce et al. (1980)[16] and Gauthier et al. (2000)[17] did not account for the ionic strength of the solution[18], the p$K_a$, determined by Sancenon and de la Guardia (1994)[17] was selected for this study. Due to the large error of the p$K_a$, the second-order rate constant of the deprotonated species has a large intrinsic error (see below).

The determined apparent second-order rate constants were plotted as a function of the fraction of the deprotonated form of carbendazim (Figure S21). The intercept (x=0) of the linear regressions is the species-specific second-order rate constant of the protonated, neutral form, while the slope is the species-specific second-order rate constant of the deprotonated form (Figure S21). The linear regression of the form y=ax+b gave the following values: a = 4×10$^6$, b = (4.3±0.2)×10$^3$. Thus, the following species-specific second-order rate constants were determined:

$$k_{O_3\text{+carbendazim}} = (4.3\pm0.2)\times10^3\ M^{-1}s^{-1}$$

$$k_{O_3\text{+carbendazim–}H^+} = 4\times10^6\ M^{-1}s^{-1}$$

The species-specific $k_{O_3}$ for the deprotonated species has been calculated on the basis of the p$K_a$ = 10.8. For p$K_a$ = 10.5, the same method would result in $k_{O_3\text{+carbendazim–}H^+}$ = 2×10$^6$ $M^{-1}s^{-1}$ and for p$K_a$ = 11.1 it would result in $k_{O_3\text{+carbendazim–}H^+}$ = 8×10$^6$ $M^{-1}s^{-1}$. Thus, the error margin of the p$K_a$ introduces a high uncertainty in the determined $k$($O_3$, carbendazim–$H^+$). The determined species-specific second-order rate constant of the neutral species is not significantly affected in the range of p$K_a$ = 10.5–11.1.

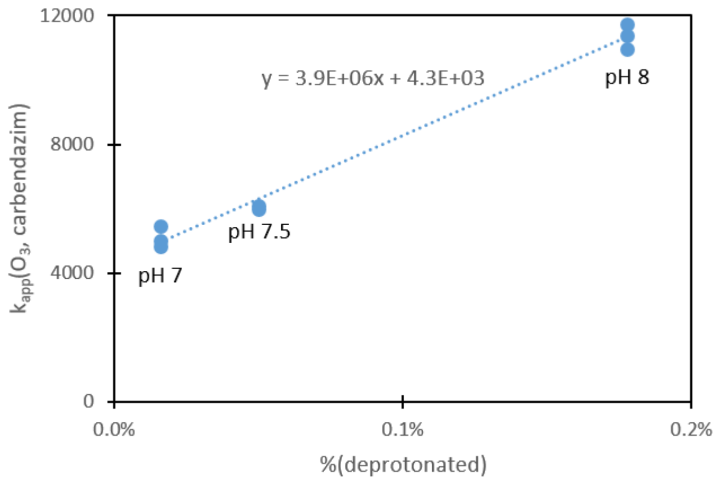


**Figure S21** – Apparent second-order rate constants for the reaction of carbendazim with ozone at different pH, plotted as a function of the fraction of the deprotonated species of carbendazim at the respective pH. The intercept of the linear regression is equivalent to the species-specific second-order rate constant for the protonated, neutral form and the slope corresponds to the species-specific second-order rate constant of the deprotonated form.

# S9 MS Spectra of nitro products

**Table S11** – LC-MS information about the LC-HRMS analysis of nitro compounds formed from model compounds.

| Precursor compound | Chemical Structure | Molecular Formula of nitro compound | Detection mode | m/z | RT (min) |
|---|---|---|---|---|---|
| 2-Aminobenzimidazole | 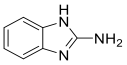 | C7H6N4O2 | + | 179.0568 | 9.06 (major), 12.79 (minor) |
| 4-Chlorophenol | 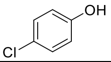 | C6H4ClNO3 | - | 171.9801 | 17.11 |
| 2,5-Dimethylphenol | 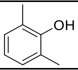 | C8H9NO3 | - | 166.0504 | 16.78 |
| 4-Hydroxybenzoic acid | 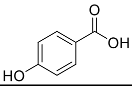 | C7H5NO5 | - | 182.0089 | 14.06 |
| Benzothiazole | 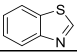 | C7H4N2SO2 | + | 181.0065 | 13.77 |
| Benzoic acid | 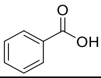 | C7H5NO4 | - | 166.014 | 11.41 and 14.66 |
| Bisoprolol* | 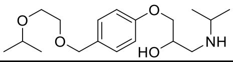 | C18H30N2O6 | + | 371.2181 | 17.52 |
| Bisphenol A | 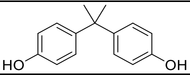 | C15H15NO4 | - | 272.0923 | 18.37 |
| Carbendazim | 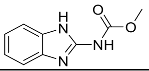 | C9H8N4O4 | + | 237.0625 | 15.07 (major), 16.61 (minor) |
| Diuron | 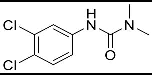 | C9H9Cl2N3O3 | + | 278.0098 | 16.64 (major) and 18.98 (minor) |
| Imidacloprid | 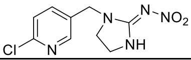 | C9H9ClN6O4 | - | 299.0295 | 12.61 and 13.31 |
| Imidacloprid Urea | 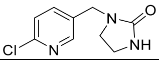 | C9H9ClN4O3 | + | 257.0441 | 11.36, 12.77 and 13.08 (major) |
| Linezolid | 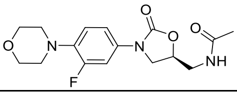 | C16H19FN4O6 | + | 383.1366 | 15.22 (major), and 18.20 (minor) |
| Methadone | 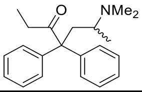 | C21H26N2O3 | + | 355.2021 | 13.73, 14.06 and 18.61 |
| Paracetamol | 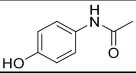 | C8H8N2O4 | + | 197.0562 | 13.05 |
| Phenol | 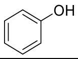 | C6H5NO3 | - | 138.0191 | 14.12 |
| Propranolol | 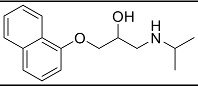 | C16H20N2O4 | + | 305.1501 | 12.78 and 13.14 |
| Ranitidine | 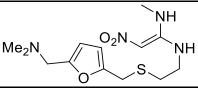 | C13H21N5O5S | Not detected | Not det. | Not det. |
| Resorcinol | 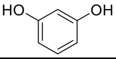 | C6H5NO4 | - | 154.014 | 14.58 |
| Tramadol | 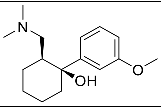 | C16H24N2O4 | + | 309.1814 | 10.23, 10.66, 11.10, and 11.61 |

Nitro compounds were detected in suspect screening by searching for m/z values (with an error within ± 3 ppm) in both positive and negative mode based on the expected molecular formula of the nitro compound (-H +NO2 in comparison to the parent formula). The mode which led to higher sensitivity was used to monitor the peak areas in all samples. Since the nitro compound under conditions in which the precursor compound is known and the transformation consisted of replacement of a hydrogen by $NO_2$ , the compounds are considered to be identified tentatively at confidence levels 2-3 according to Schymanski et al.[19] The extracted ion chromatograms and corresponding MS spectra of the detected nitro compounds are shown below. The molecular ions used for the EIC are highlighted in green in the corresponding MS spectra.

2-Aminobenzimidazole nitro products

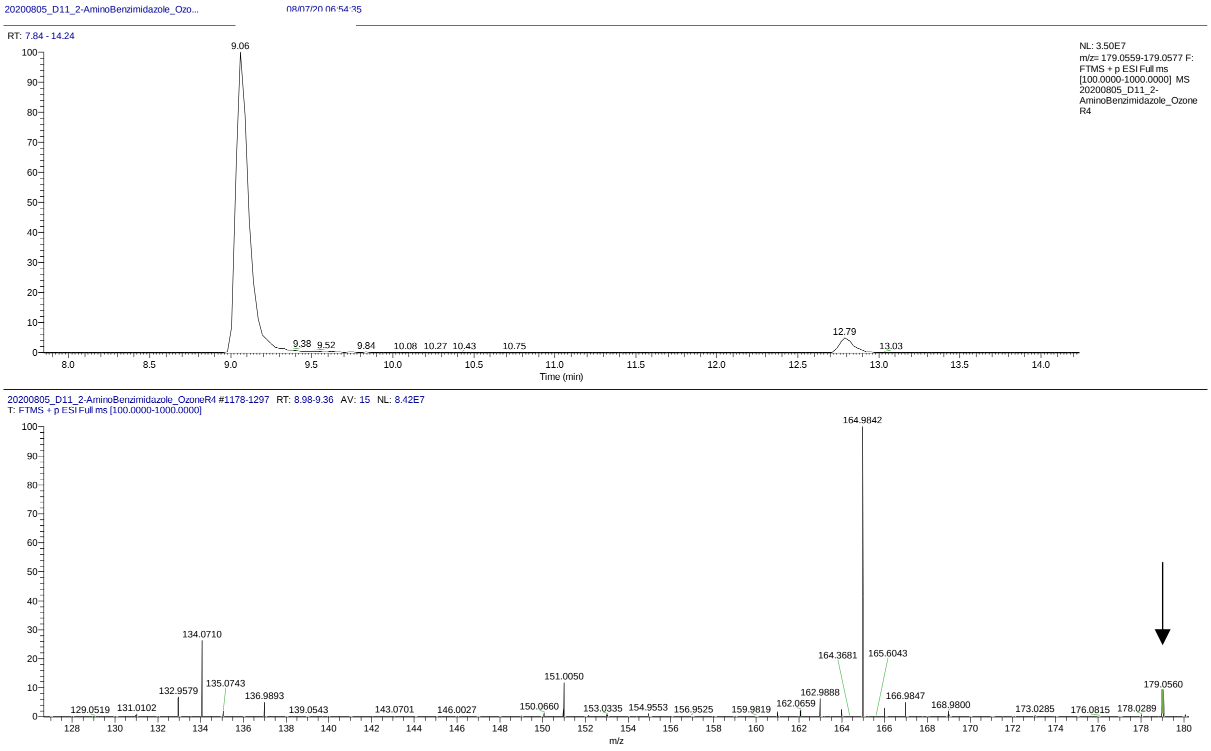

# 4-Chlorophenol nitro product

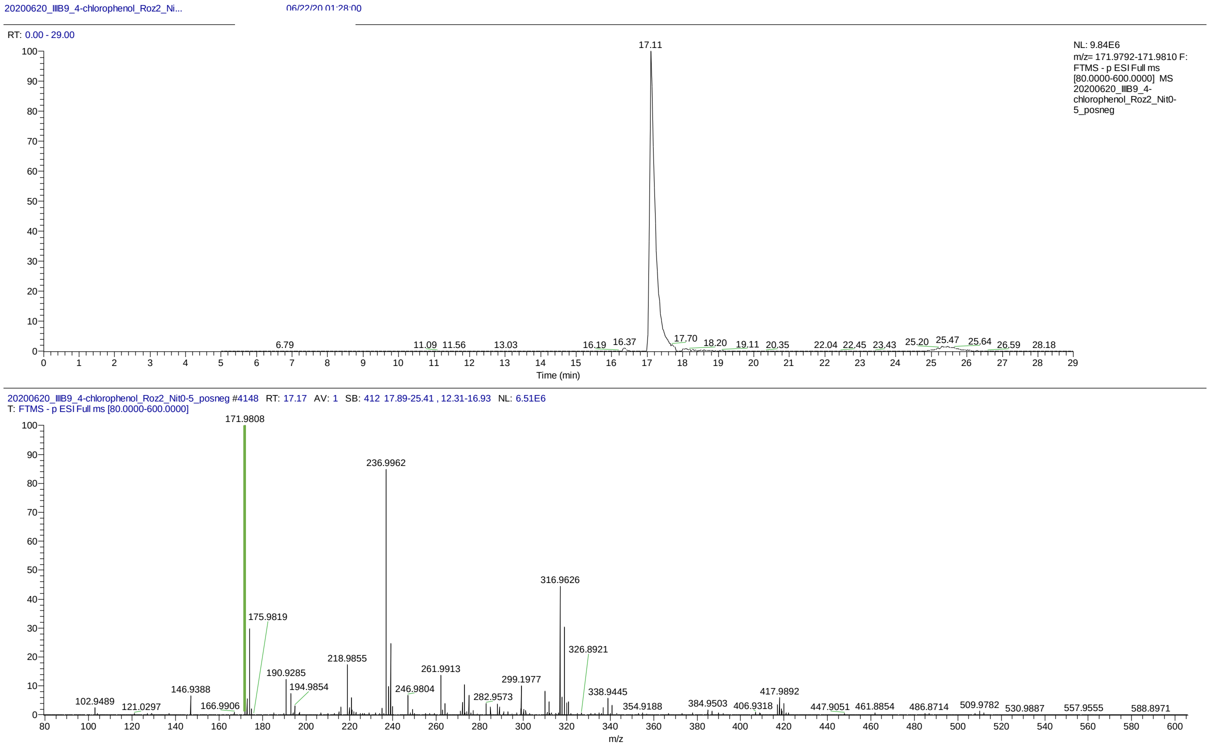


# 2,5-Dimethylphenol nitro product

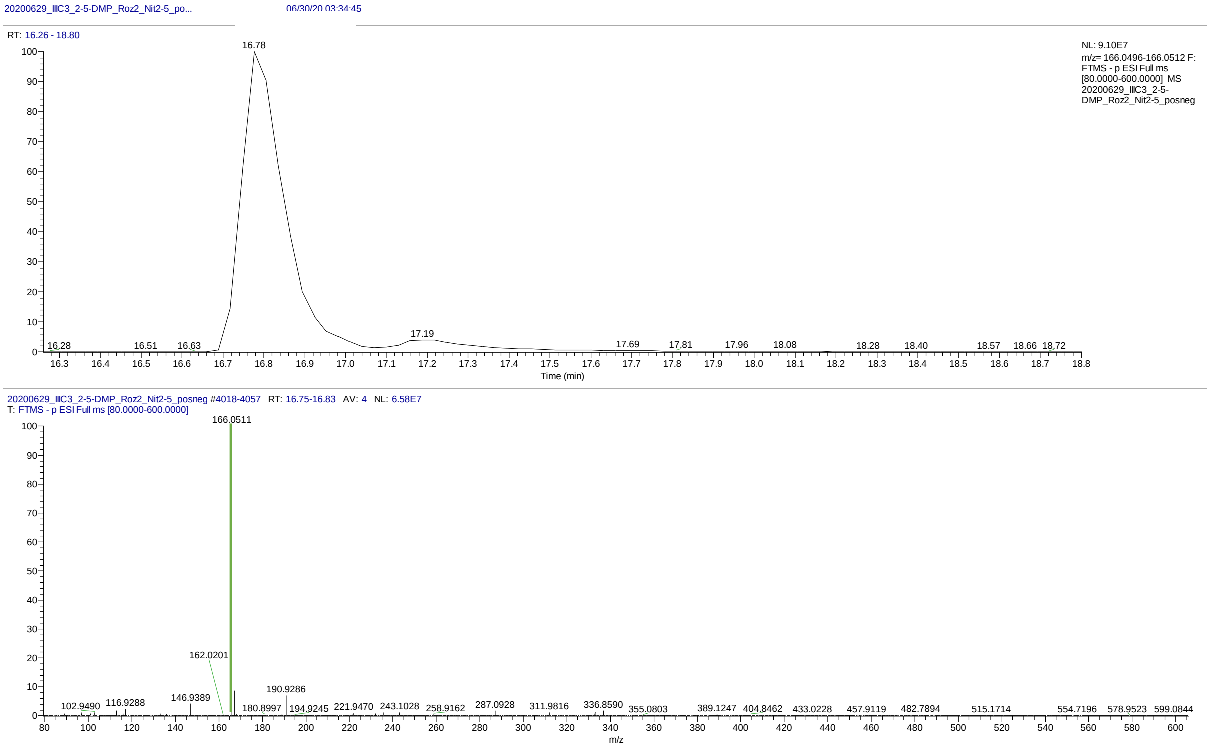

# 4-Hydroxybenzoic acid nitro product

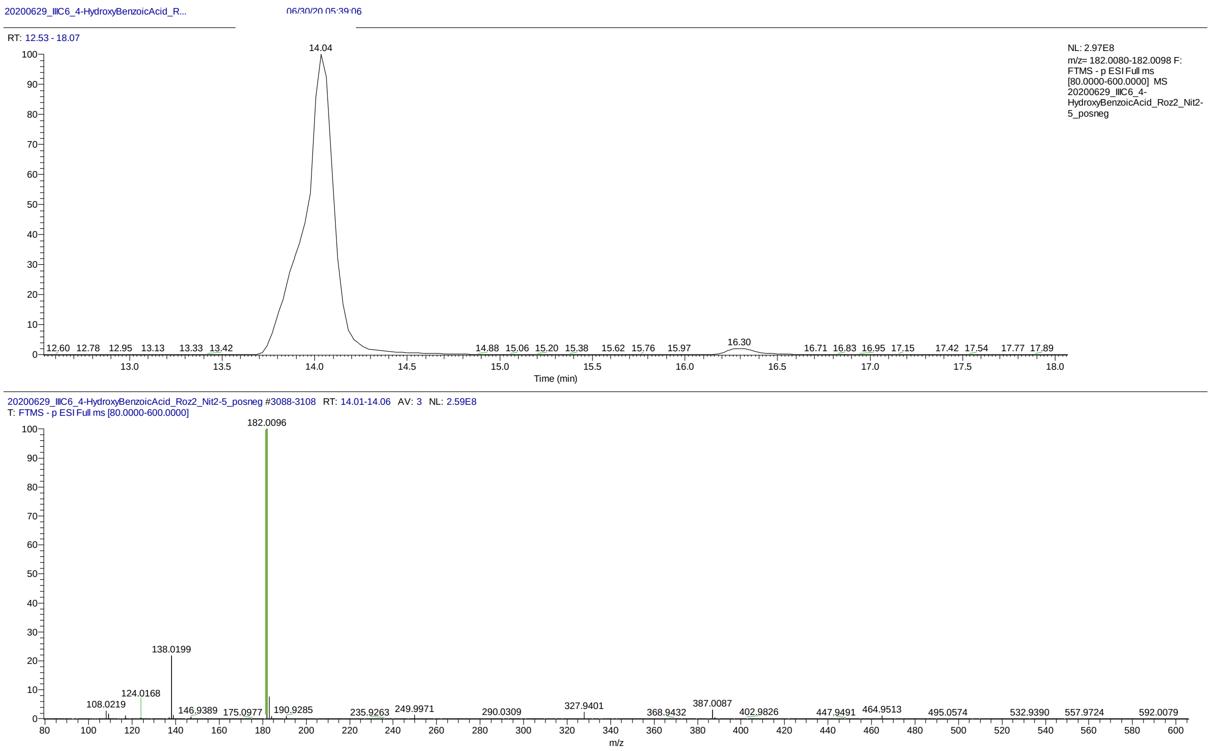


# Benzothiazole nitro product

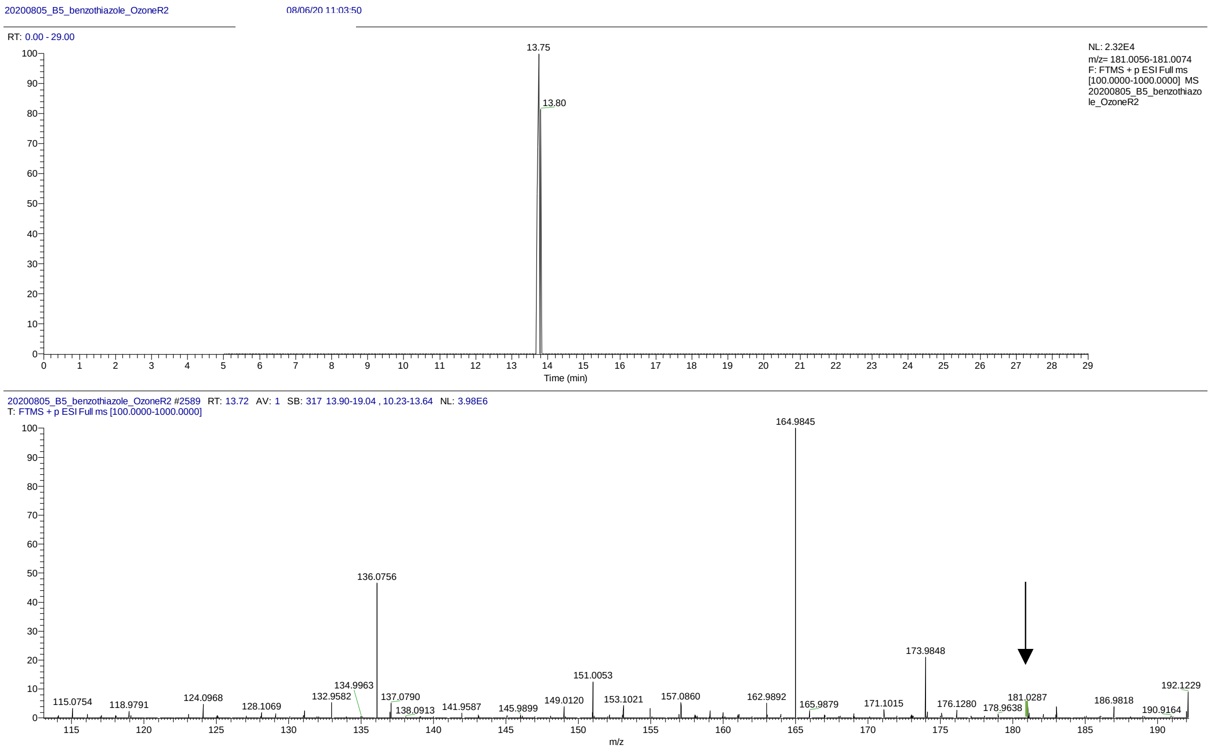

# Benzoic acid nitro product

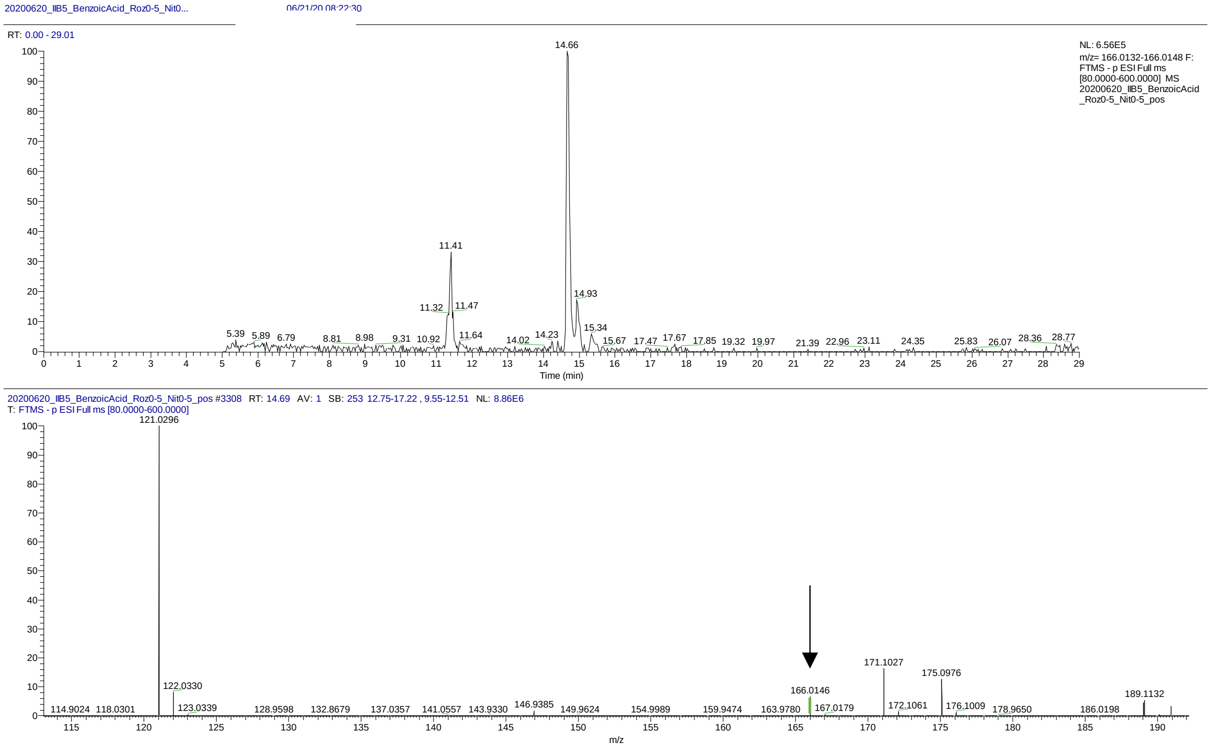


# Bisoprolol nitro product

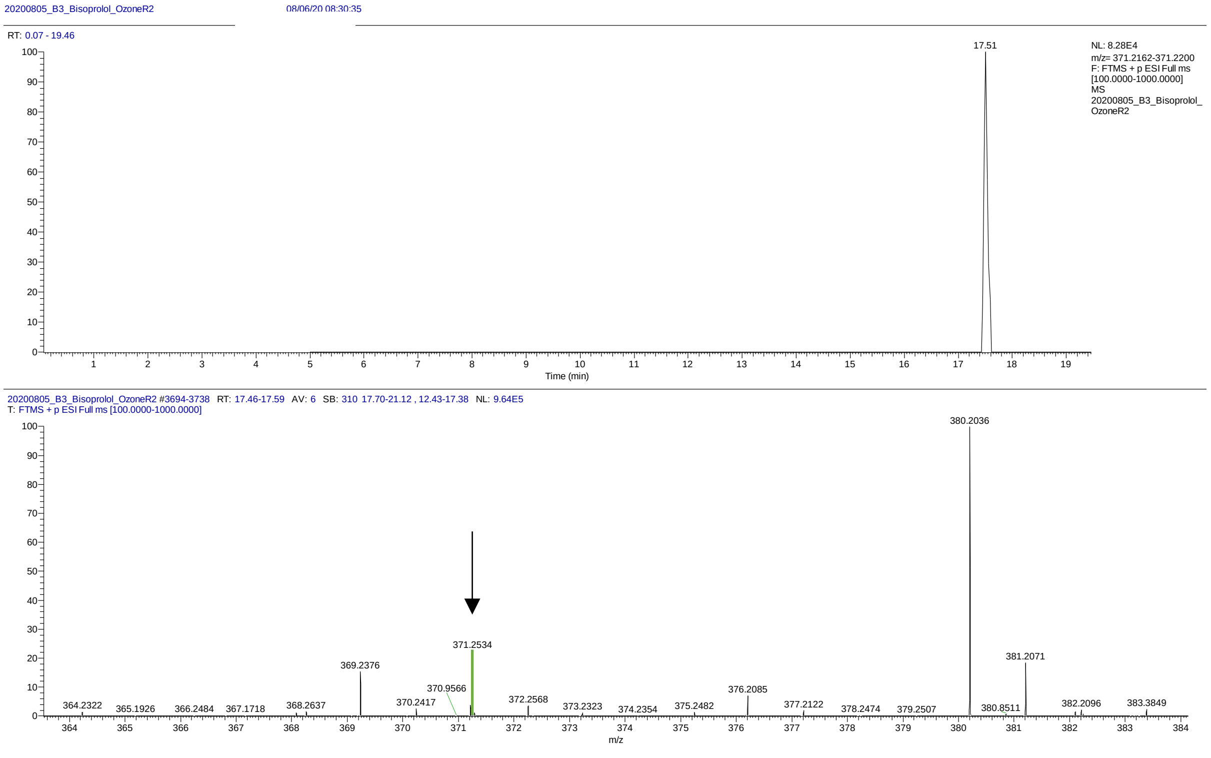

# Bisphenol A nitro product

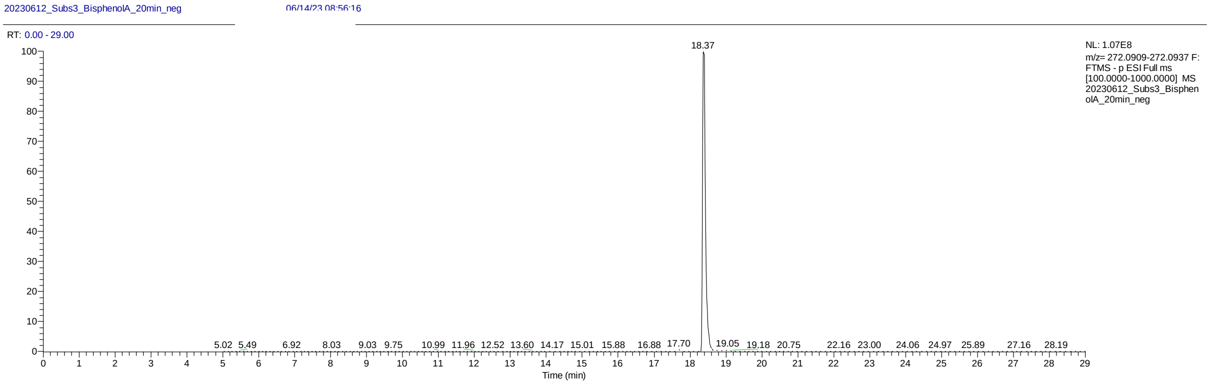


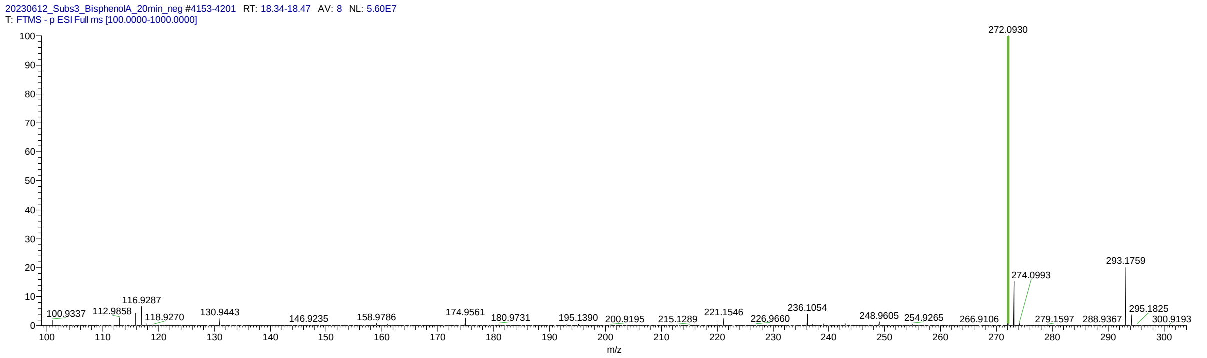


# Carbendazim nitro products

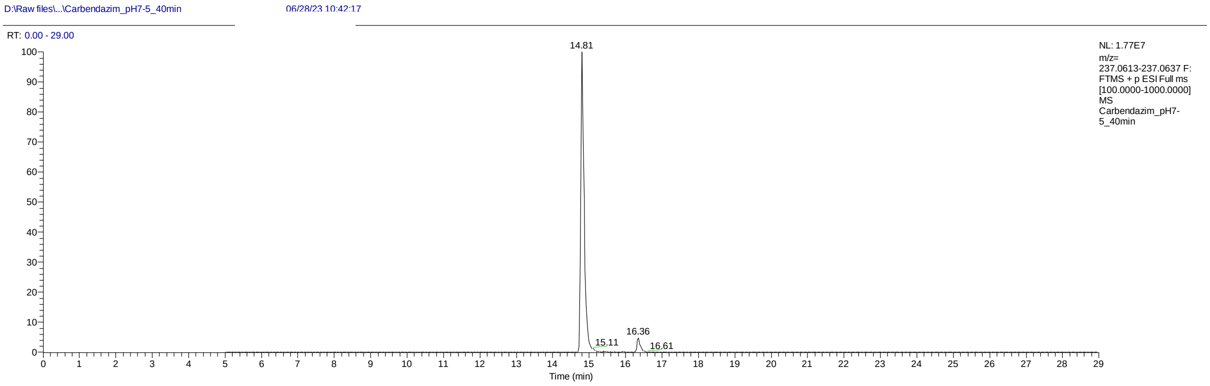


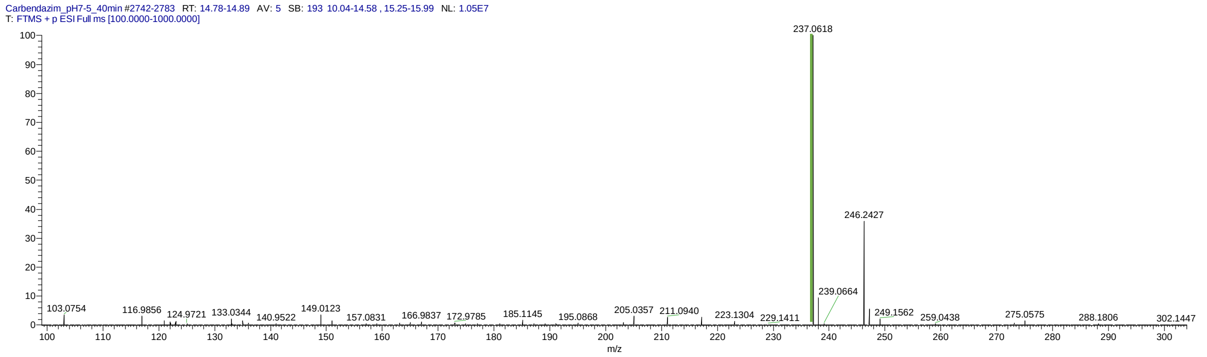

## Diuron nitro products

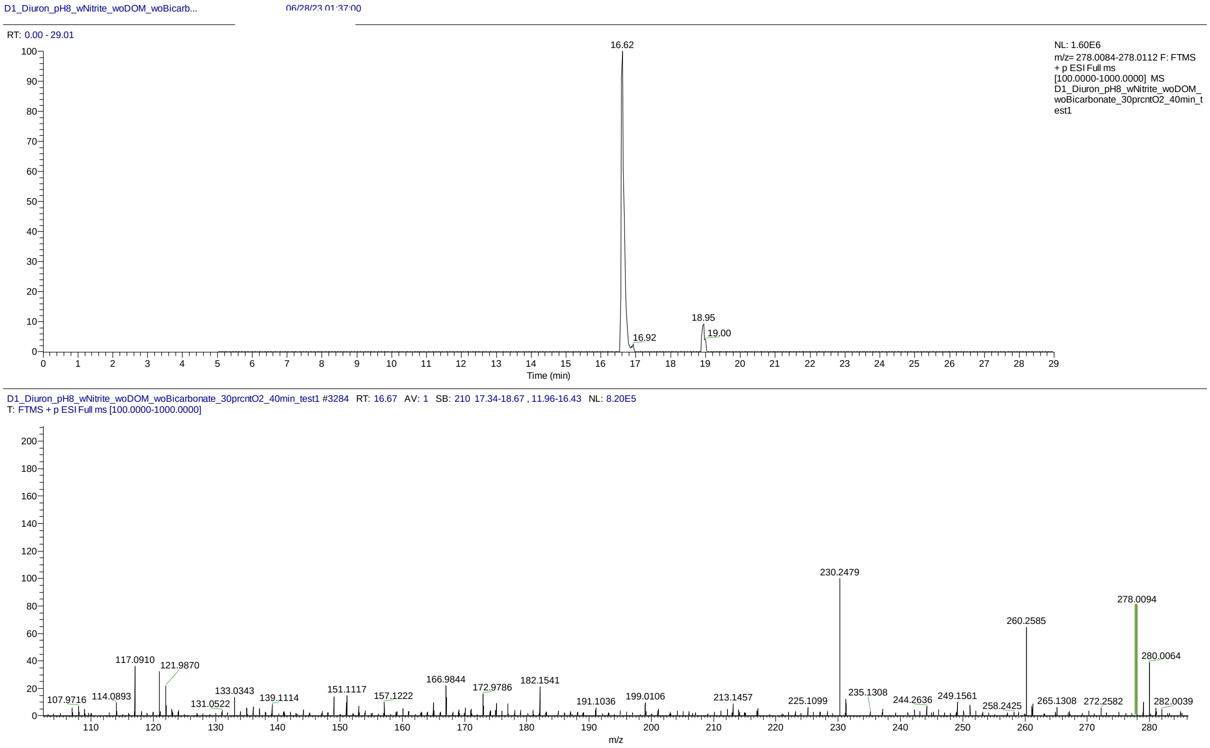


## Imidacloprid nitro products

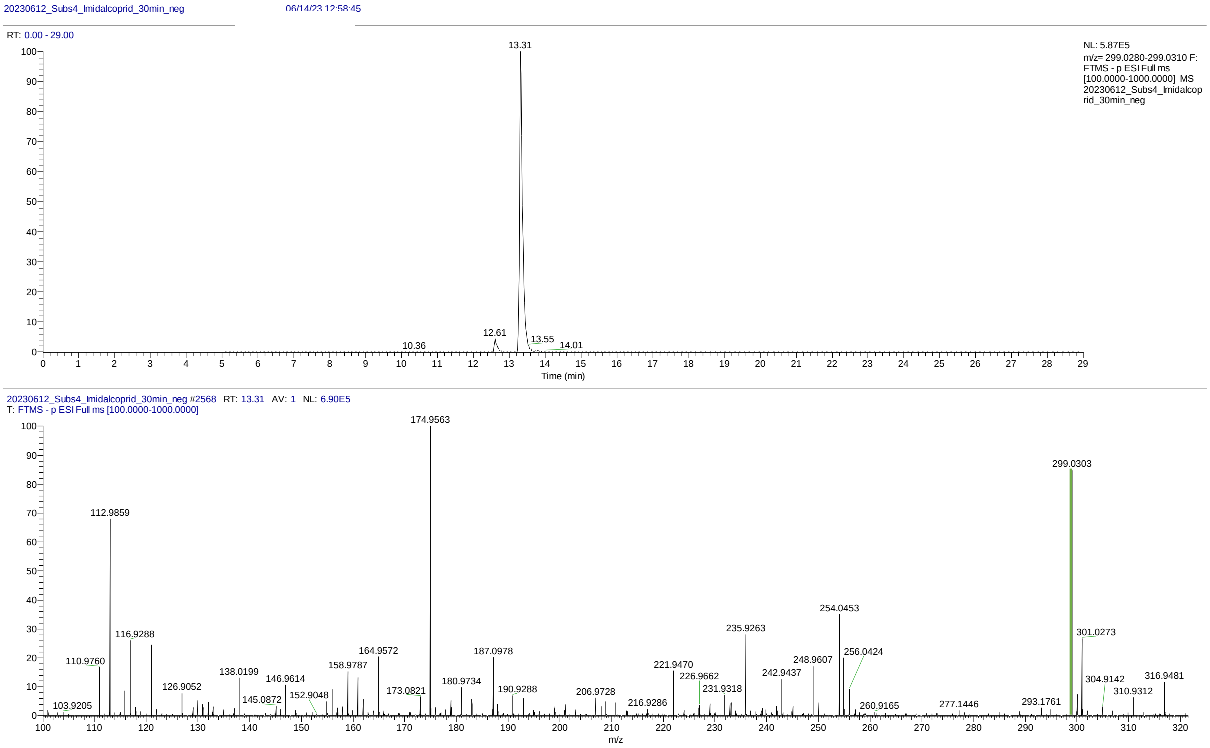

# Imidaclopridurea nitro products

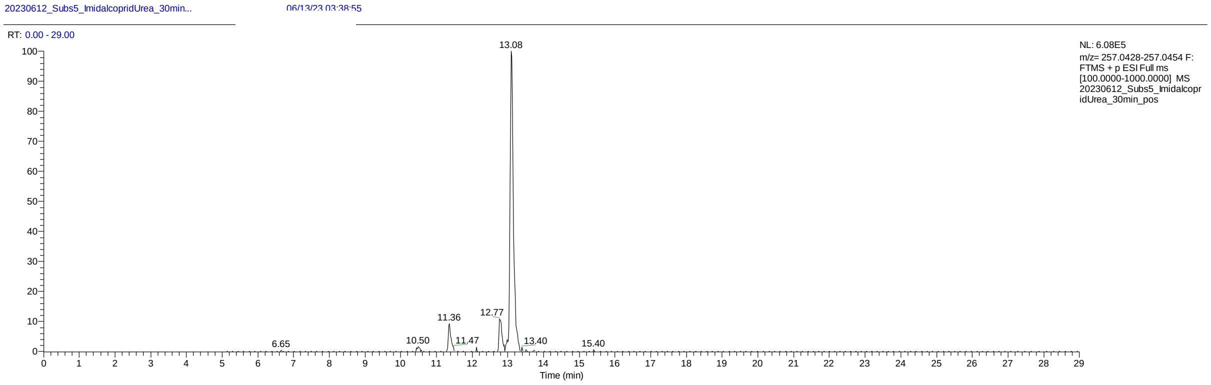


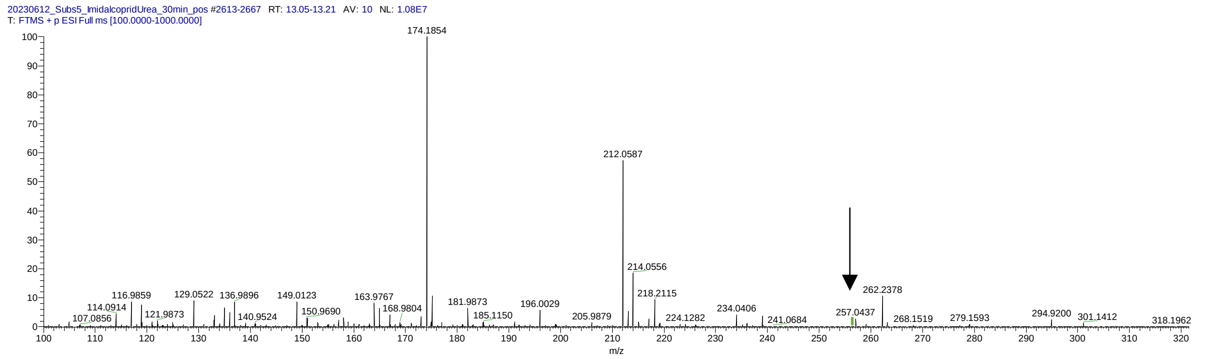


# Linezolid nitro product

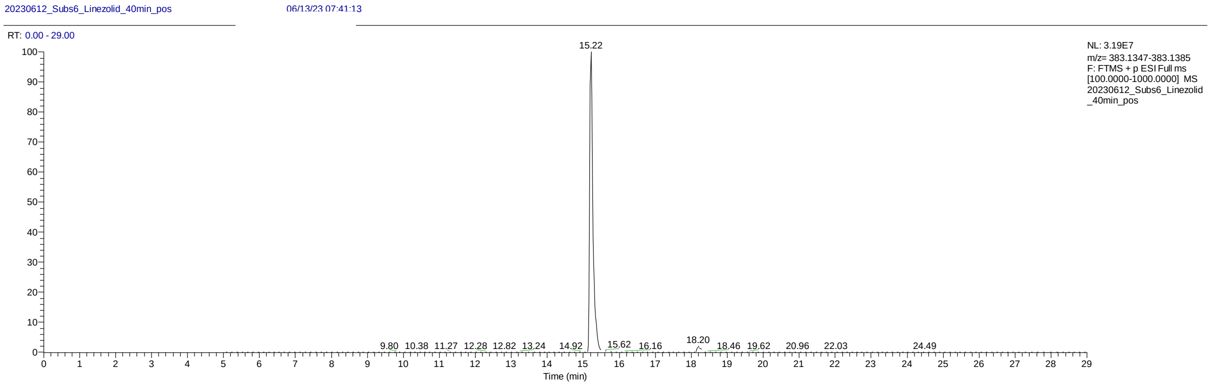


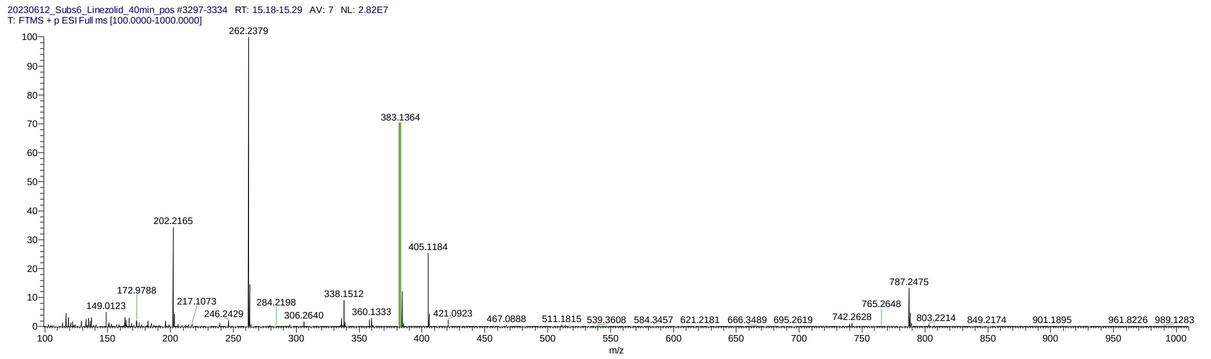

# Methadone nitro product

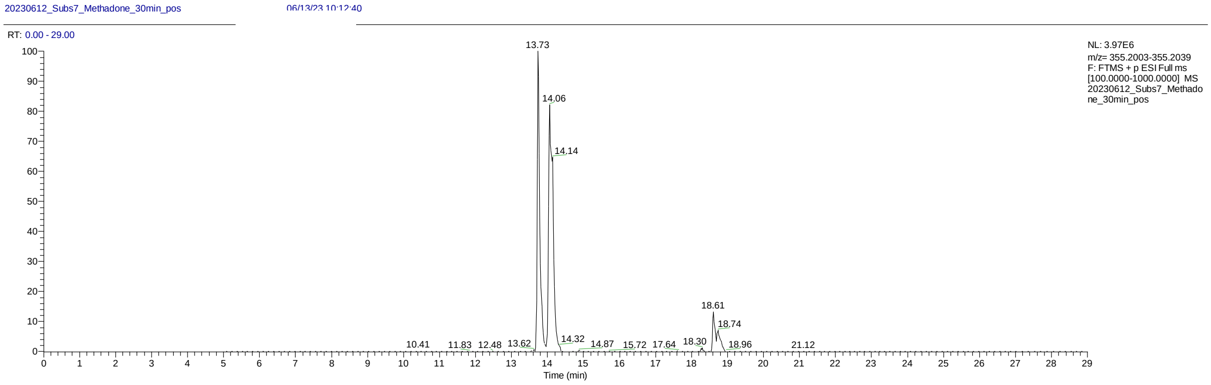


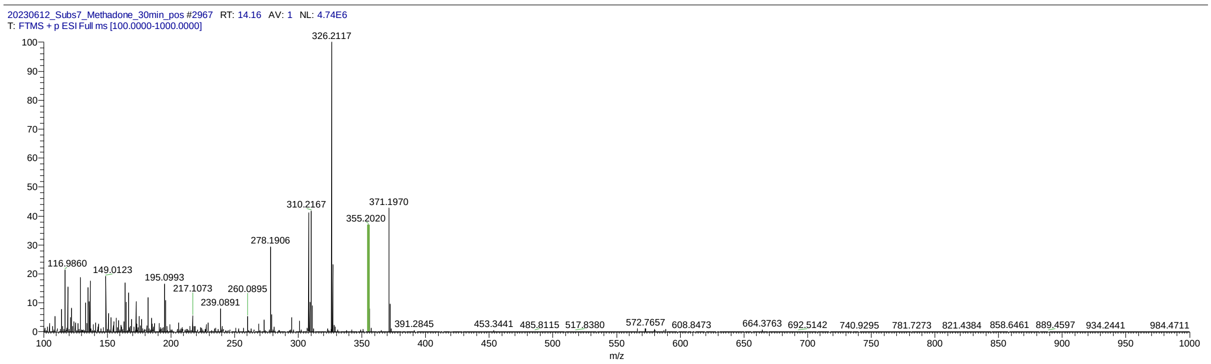


# Paracetamol nitro products

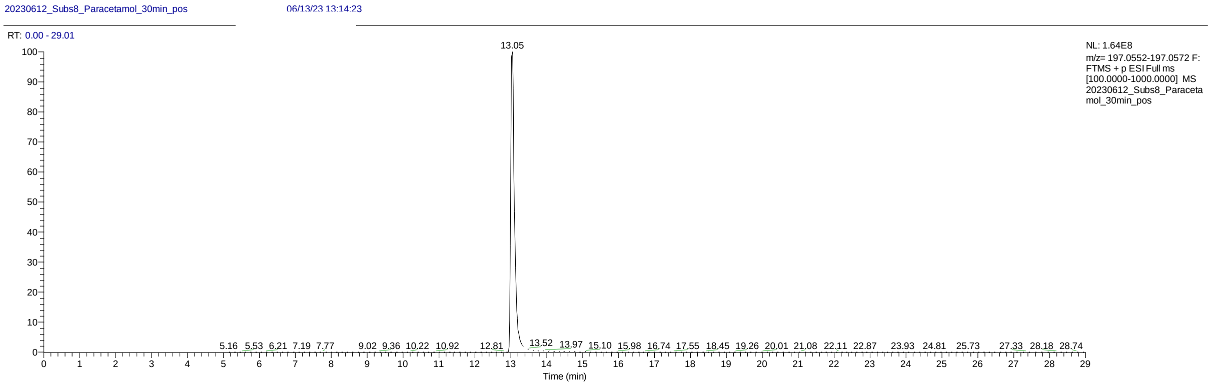


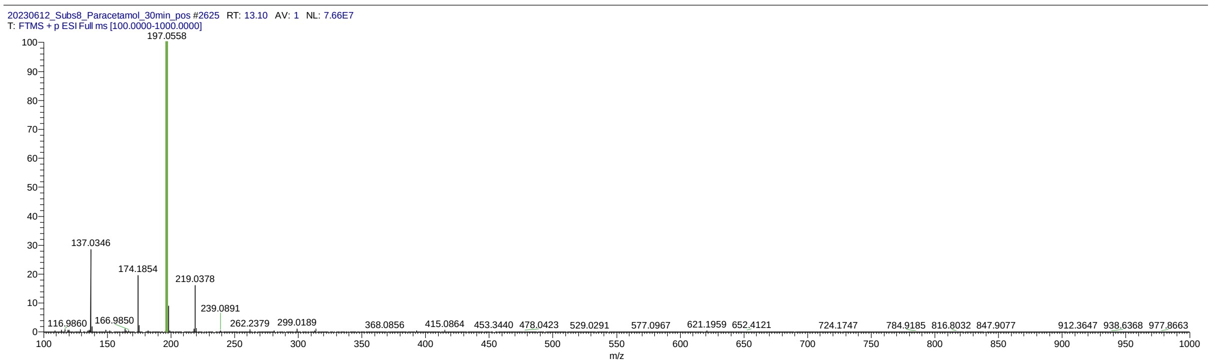

# Phenol nitro product

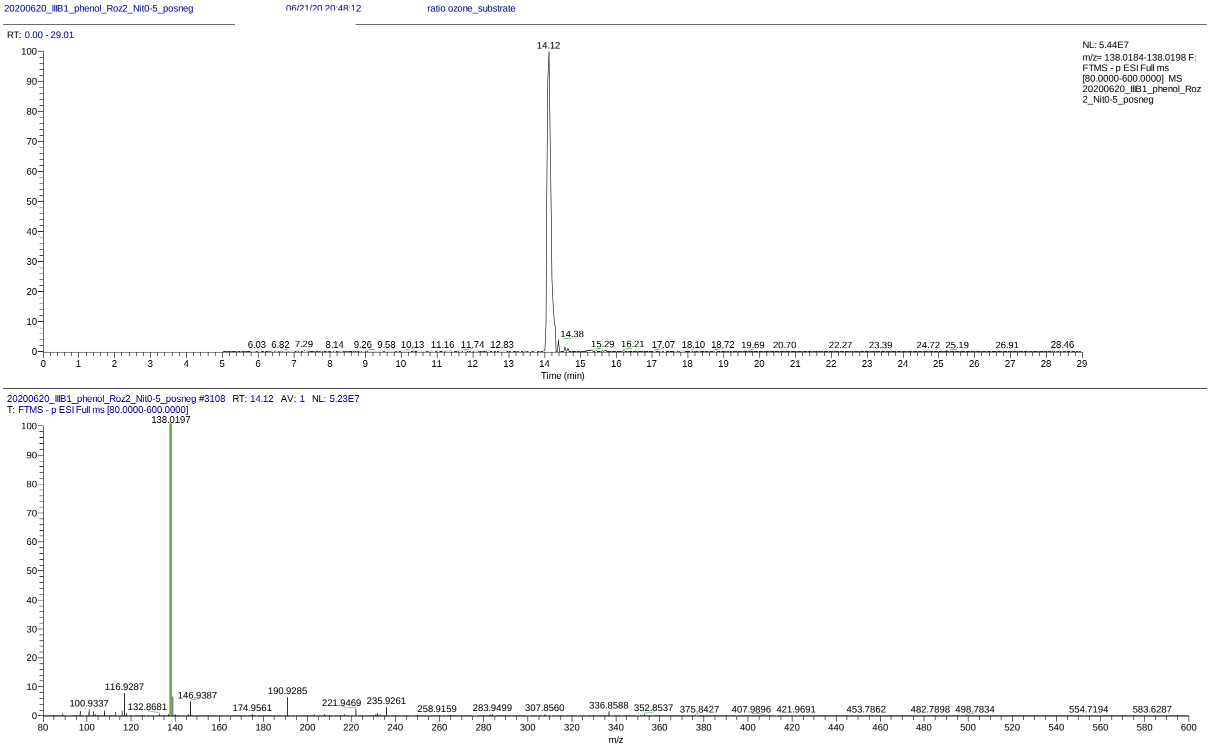


# Propanolol nitro products

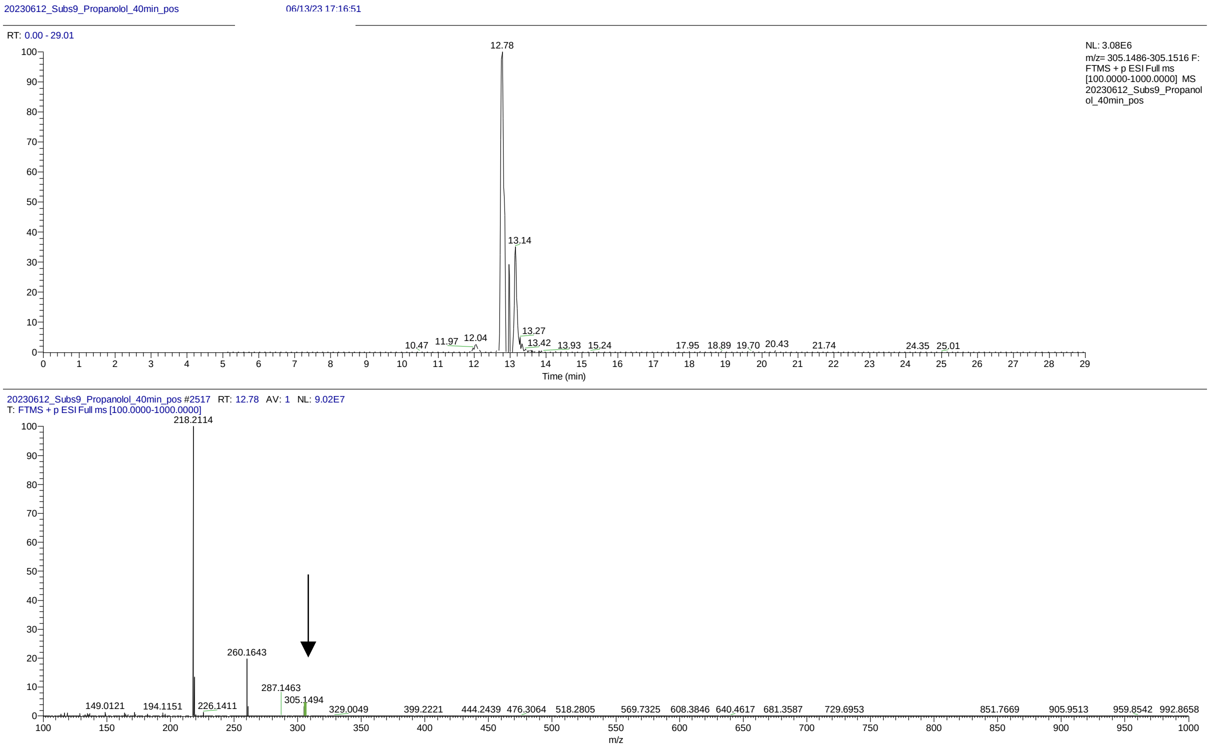

# Resorcinol nitro products

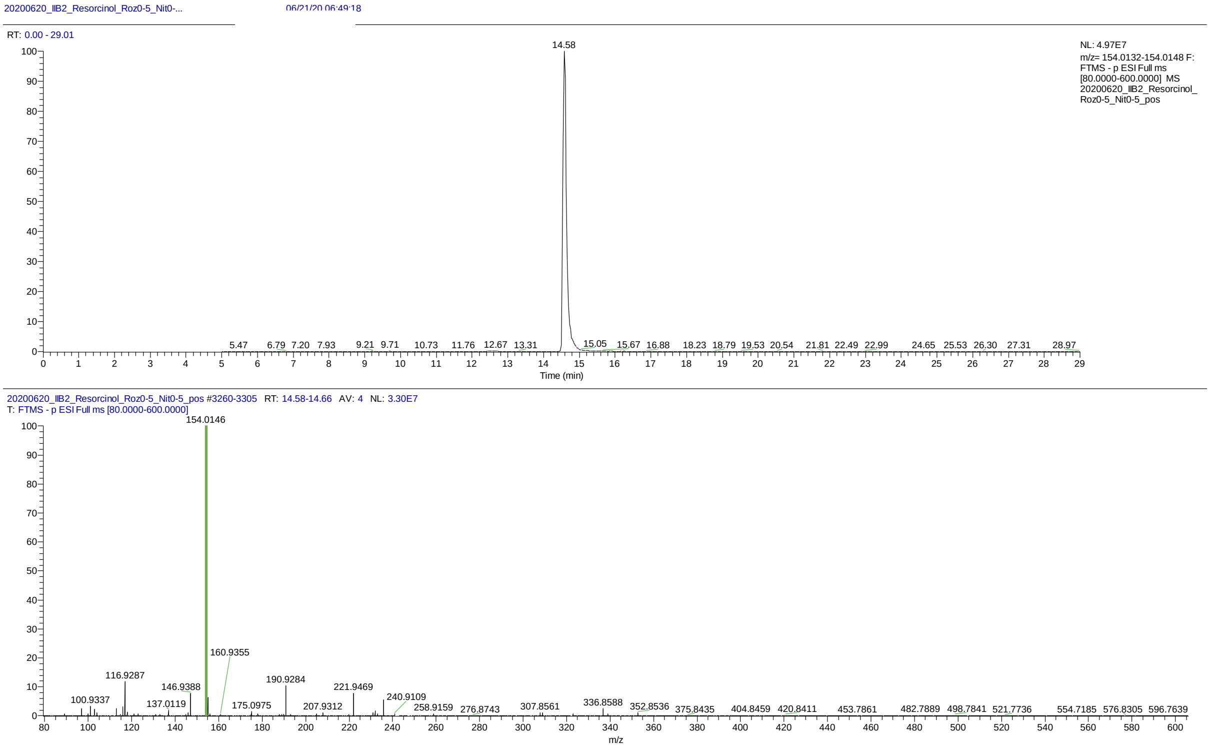


# Tramadol nitro products

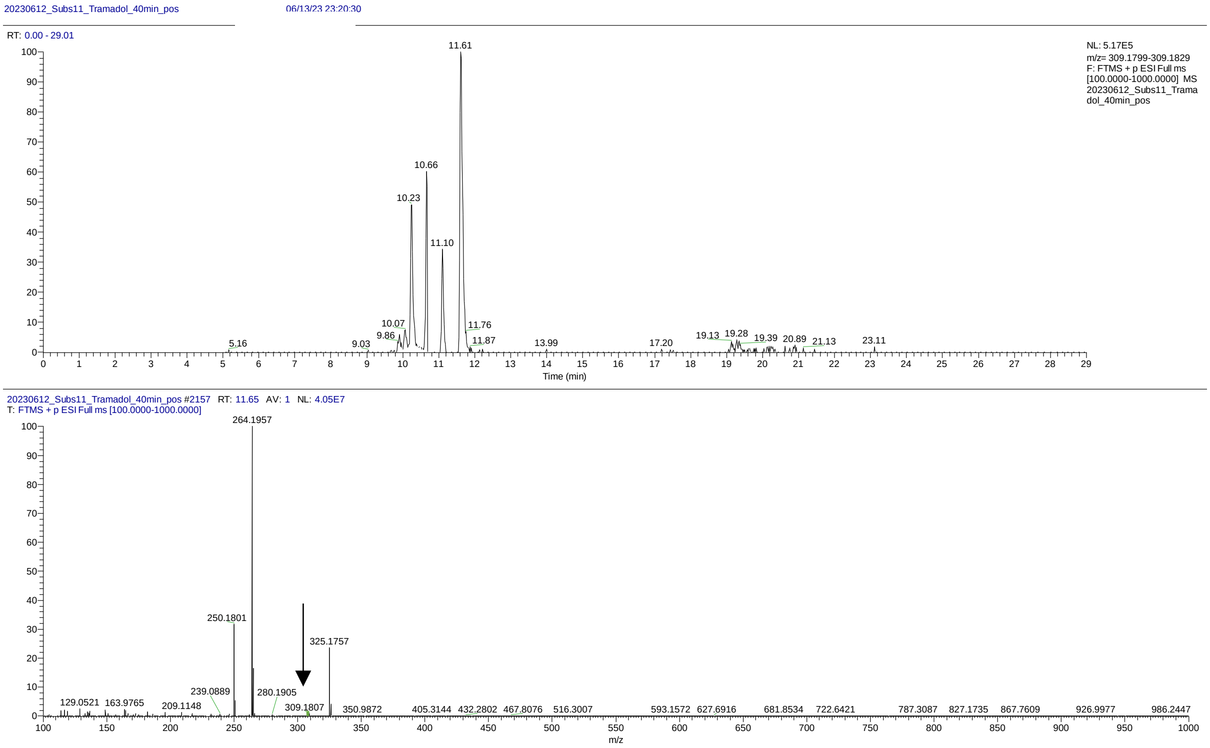

# S10 NMR Experiments

**$^{1}H$ and $^{13}C$ NMR chemical shift assignments of compounds A, B and C (in synthesized nitrodiuron mixture)**

$^{1}H$ and $^{13}C$ NMR data were recorded on a Bruker AV-III 400 spectrometer (Bruker BioSpin AG, Switzerland) at 400.2, and 100.6 MHz, respectively. The 1D $^{1}H$ and $^{13}C$ NMR spectra, as well as the 2D correlated $^{1}H$-$^{13}C$ HSQC and $^{1}H$-$^{13}C$ HMBC NMR experiments were performed at 298 K using the Bruker standard pulse programs and parameter sets on a 5 mm CryoProbe™ Prodigy probe. The $^{1}H$ and $^{13}C$ NMR chemical shifts are calibrated with respect to the resonances of the chloroform solvent at 7.26 and 77.0 ppm, respectively. Coupling constants *J* are reported in Hz and for $^{1}H$ NMR data coupling patterns are described as *s* = singlet, *d* = doublet, *br* = broad and for $^{13}C$ NMR data *s* = quaternary carbon, *d* = CH and *q* = $CH_3$.

- Compound **A** in Figure S22/ 3-(4,5-dichloro-2-nitrophenyl)-1,1-dimethylurea **(nitrodiuron isomer 2):**

$^{1}H$ NMR ($CDCl_3$, 400.2 MHz): δ 10.2 (*s*, *br*, 1H, NH); 9.01 (*s*, 1H, H-6); 8.30 (*s*, 1H, H-3), 3.11 (*s*, 6H, H-8). $^{13}C$ NMR ($CDCl_3$, 100.6 MHz): δ 153.9 (s, C-7); 141.2 (*s*, C-2), 136.5 (*s*, C-5); 133.6 (*s*, C-1); 126.6 (*d*, C-3); 124.9 (*s*, C-4); 122.3 (d, C-6), 36.4 (*q*, 2C, C-8).

- Compound **B** in Figure S22/ 3-(3,4-dichloro-2,6-dinitrophenyl)-1,1-dimethylurea: **(dinitrodiuron)**

$^{1}H$ NMR ($CDCl_3$, 400.2 MHz): δ 8.4 (*s*, *br*, 1H, NH); 8.33 (*s*, 1H, H-5); 3.05 (*s*, 6H, H-8). $^{13}C$ NMR ($CDCl_3$, 100.6 MHz): δ 153.1 (s, C-7); 145.2 (*s*, C-2), 140.1 (*s*, C-6); 132.8 (*s*, C-3); 129.3 (*s*, C-4); 128.8 (*s*, C-1); 127.9 (*d*, C-5); 36.7 (*q*, 2C, C-8).

- Compound **C** in Figure S22 (not fully assignable due to low relative amount)/ 3-(3,4-dichloro-2-nitrophenyl)-1,1-dimethylurea **(nitrodiuron isomer 1):**

$^{1}H$ NMR ($CDCl_3$, 400.2 MHz): δ 8.14 (*d, J*=9.3 Hz, 1H, H-6); 7.54 (*d, J*=9.3 Hz, 1H, H-5); 3.02 (*s*, 6H, H-8). $^{13}C$ NMR ($CDCl_3$, 100.6 MHz): δ 132.6 (*d*, C-5); 122.1 (*d*, C-6).

From $^{1}H$ NMR integrals determined for the dimethyl groups (positions 8) of the three compounds, the following relative amounts have been obtained: 79.3% **A**, 19.3 % **B** and 1.4 % **C** [mol/mol-%].

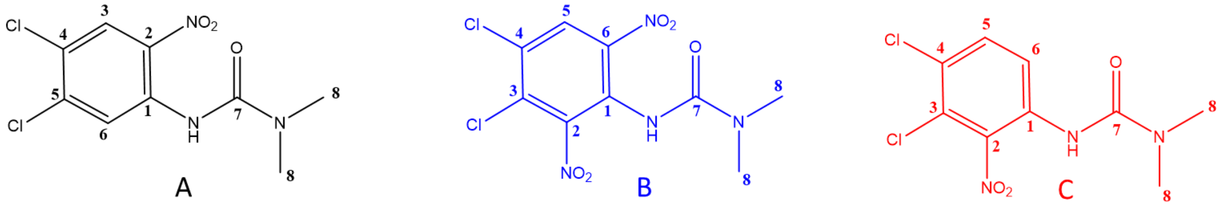


3-(4,5-dichloro-2-nitrophenyl)-1,1-dimethylurea

3-(3,4-dichloro-2,6-dinitrophenyl)-1,1-dimethylurea

3-(3,4-dichloro-2-nitrophenyl)-1,1-dimethylurea

**Figure S22** – Chemical structures identified in "nitro-diuron" sample with indication of positions used for NMR chemical shift assignments.

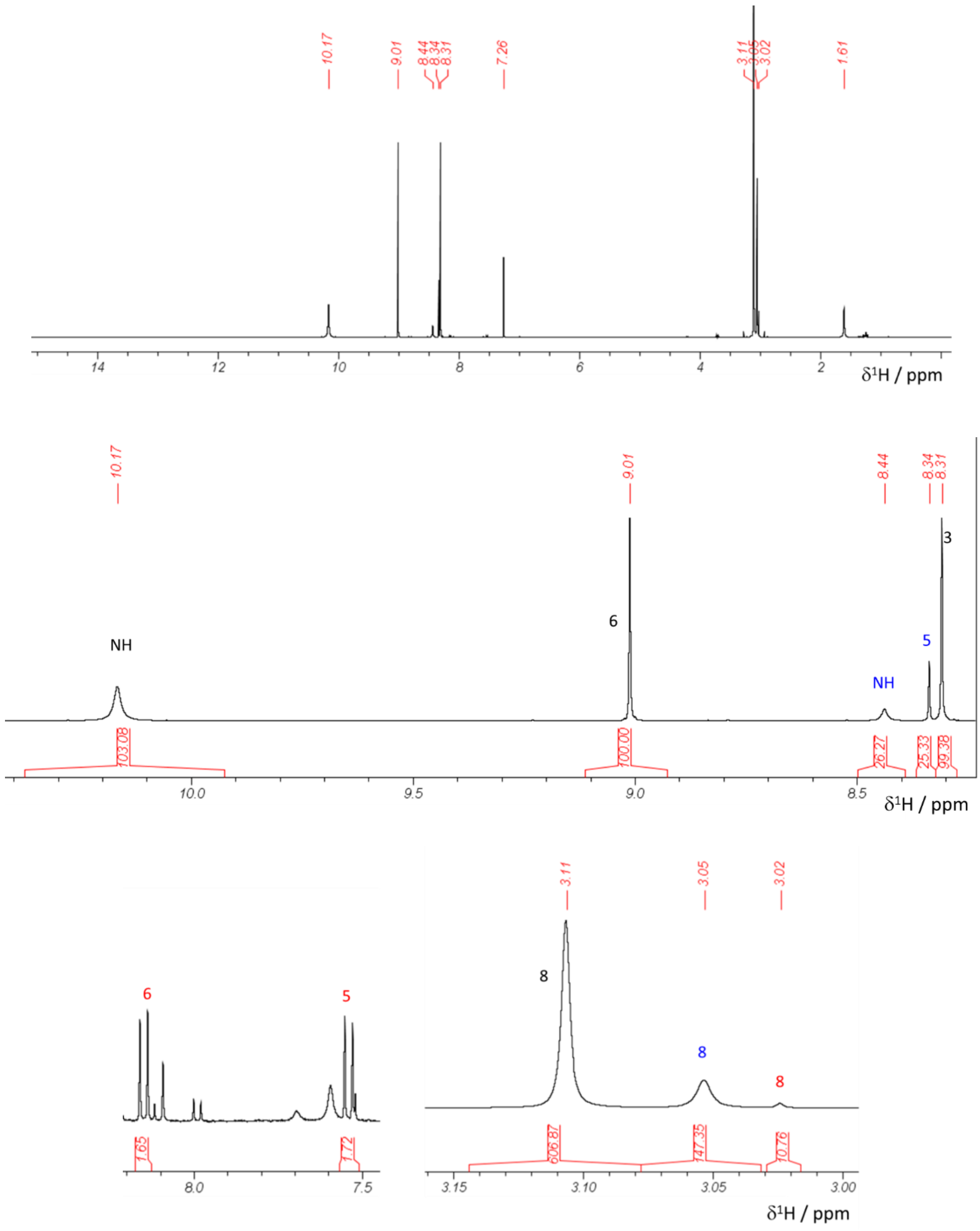


**Figure S23** – $^{1}$H NMR spectra of sample "nitro diuron": Full range and expanded chemical shift regions with assignment of resonances.

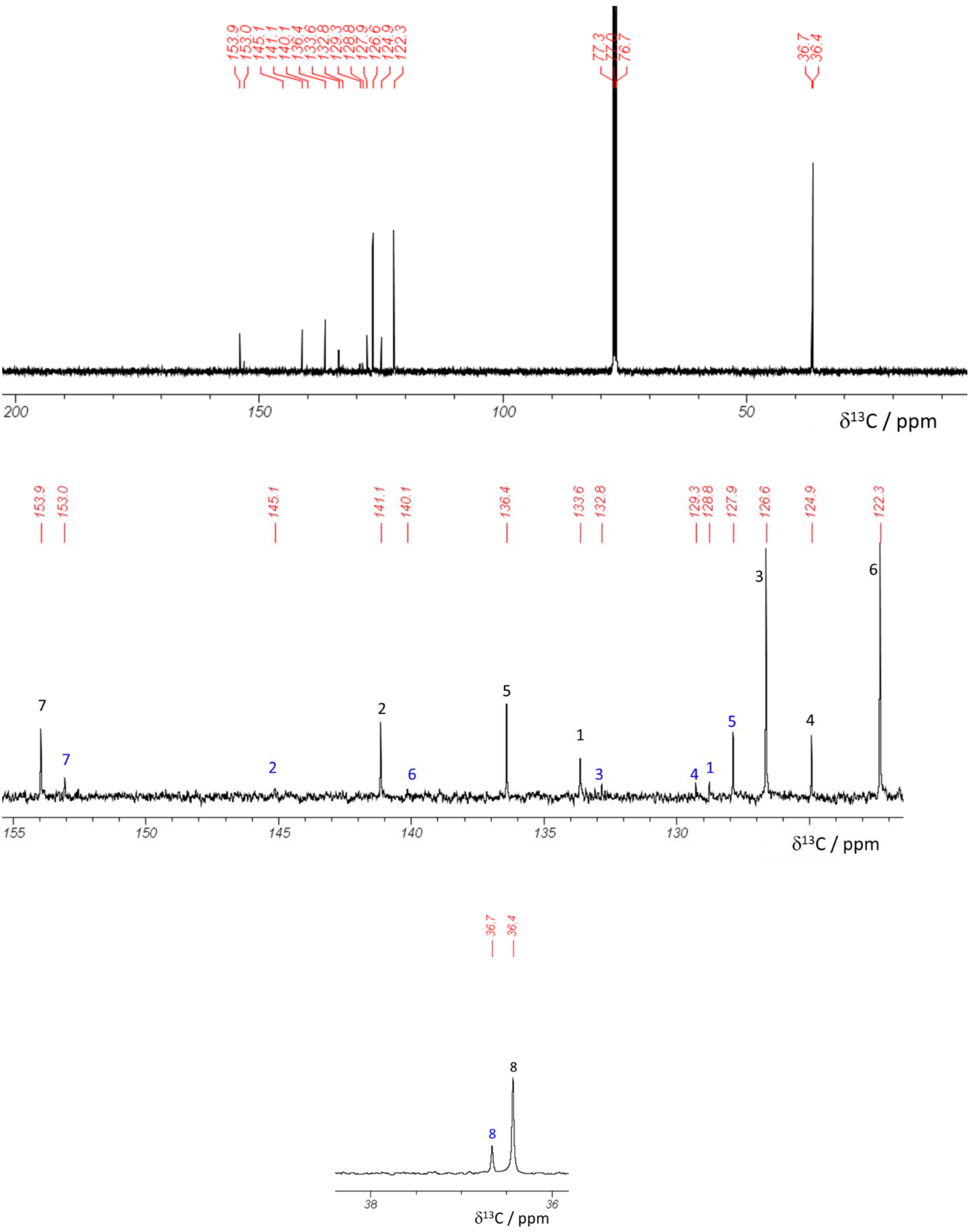


**Figure S24** – $^{13}$C NMR spectra of sample "nitro diuron": Full range and expanded chemical shift regions with assignment of resonances.

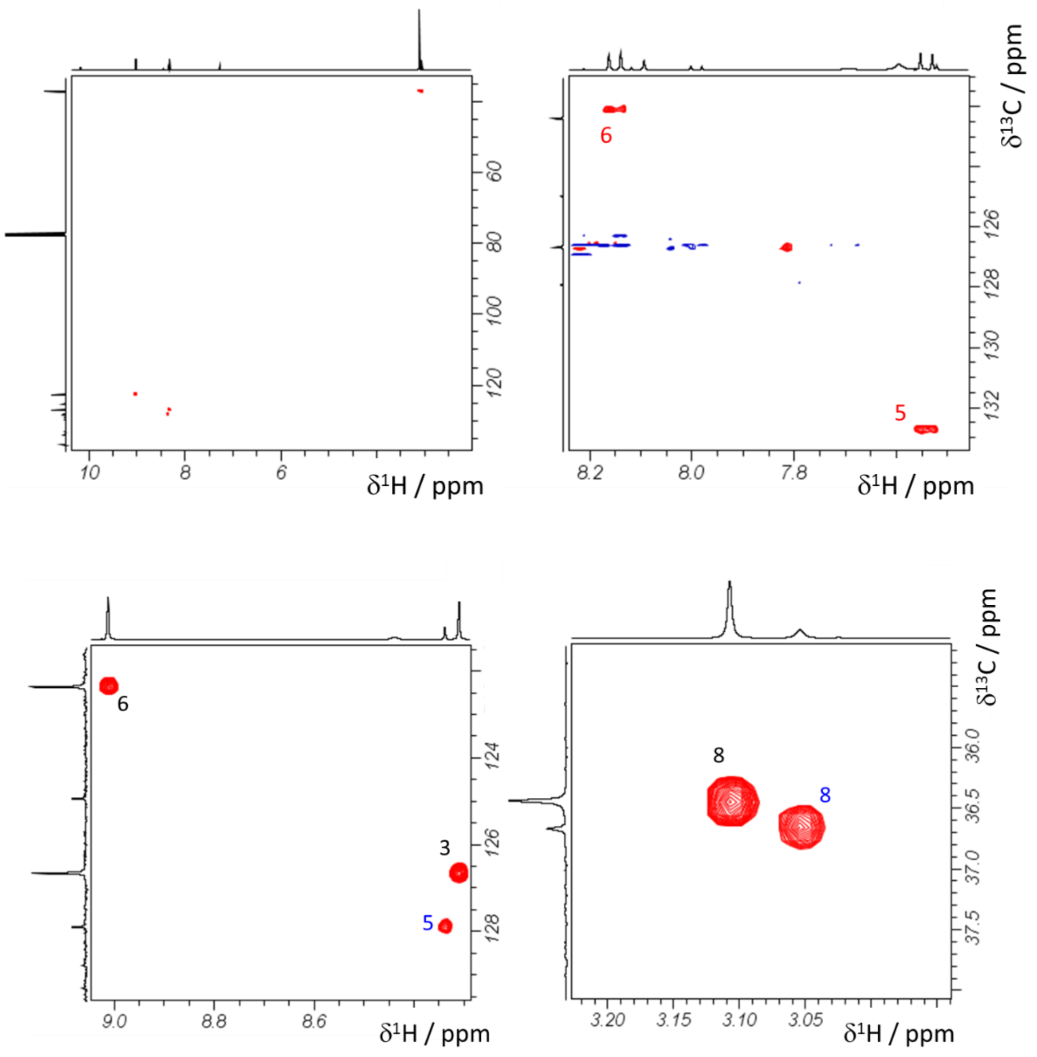


**Figure S25** – $^{1}H$-$^{13}C$ HSQC NMR spectrum of sample "nitro diuron": Full range and expanded chemical shift regions with assignment of resonances.

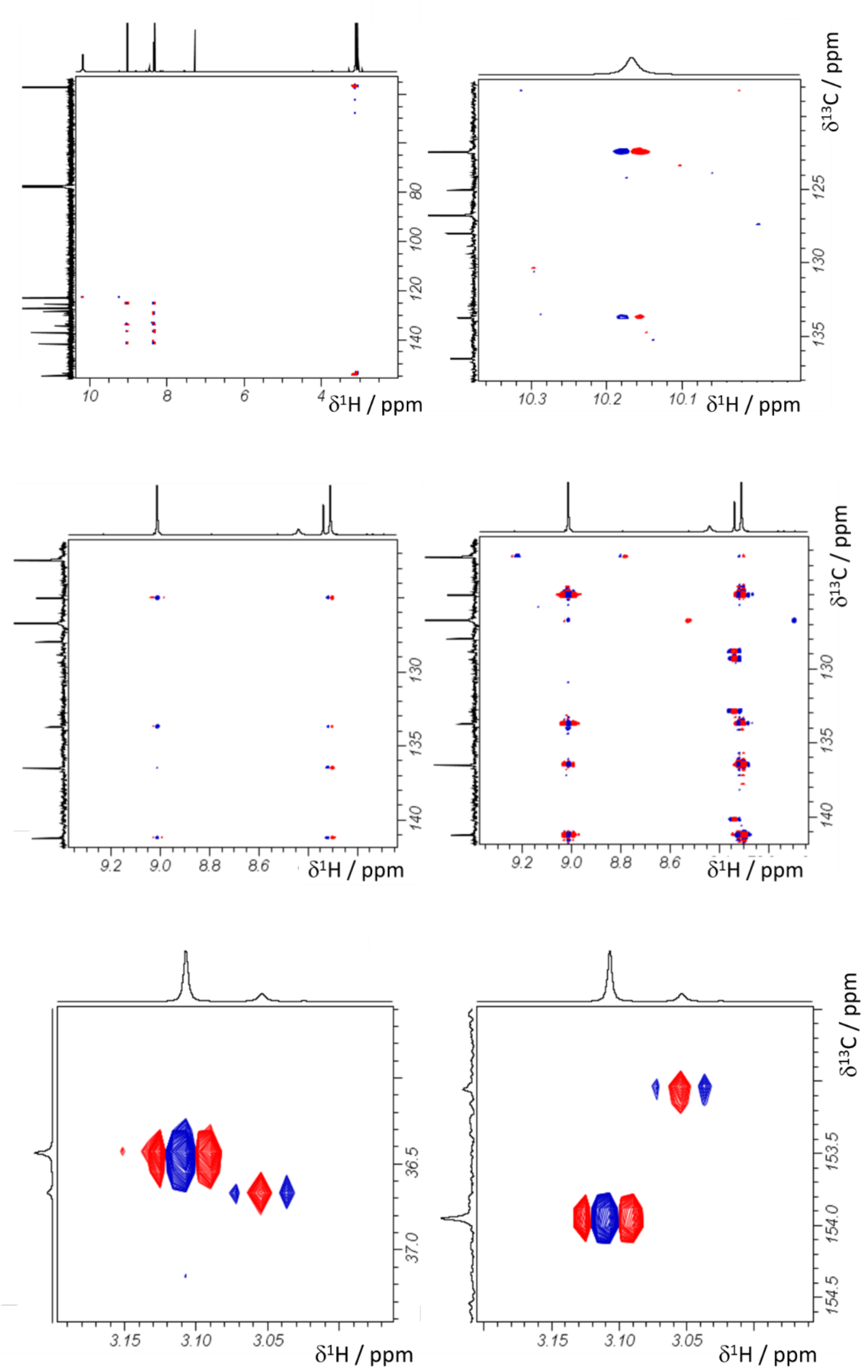


**Figure S22** – $^{1}H$-$^{13}C$ HMBC NMR spectrum of sample "nitro diuron": Full range and expanded chemical shift regions.

## S11 References


(1) Nélieu, S.; Bonnemoy, F.; Bonnet, J. L.; Lefeuvre, L.; Baudiffier, D.; Heydorff, M.; Quéméneur, A.; Azam, D.; Ducrot, P. H.; Lagadic, L. Ecotoxicological effects of diuron and chlorotoluron nitrate-induced photodegradation products: Monospecific and aquatic mesocosm-integrated studies. *Environmental Toxicology and Chemistry* **2010**, *29* (12), 2644-2652.

(2) Bader, H.; Hoigné, J. Determination of Ozone in Water by the Indigo Method. *Water Research* **1981**, *15* (4), 449-456.

(3) von Sonntag, C.; von Gunten, U. Chemistry Of Ozone In Water and Wastewater Treatment. IWA Publishing. **2012**.

(4) Rice, E. W.; Bridgewater, L.; American Public Health, A. *Standard methods for the examination of water and wastewater*; American public health association Washington, DC, 2012.

(5) Bohle, D. S.; Hansert, B.; Paulson, S. C.; Smith, B. D. Biomimetic synthesis of the putative cytotoxin peroxynitrite, ONOO-, and its characterization as a tetramethylammonium salt. *Journal of the American Chemical Society* **1994**, *116* (16), 7423-7424.

(6) Molina, C.; Kissner, R.; Koppenol, W. H. Decomposition kinetics of peroxynitrite: influence of pH and buffer. *Dalton Trans* **2013**, *42* (27), 9898-9905. DOI: 10.1039/c3dt50945a.

(7) Stumm, W.; Morgan, J. J. X. *Aquatic chemistry: chemical equilibria and rates in natural waters*; John Wiley & Sons, 2013.

(8) Solomon, T. The definition and unit of ionic strength. *Journal of Chemical Education* **2001**, *78* (12), 1691.

(9) Maurer, P.; Thomas, C. F.; Kissner, R.; Rüegger, H.; Greter, O.; Röthlisberger, U.; Koppenol, W. H. Oxidation of nitrite by peroxynitrous acid. *The Journal Of Physical Chemistry A* **2003**, *107* (11), 1763-1769.

(10) Naumov, S.; Mark, G.; Jarocki, A.; von Sonntag, C. The Reactions of Nitrite Ion with Ozone in Aqueous Solution – New Experimental Data and Quantum-Chemical Considerations. *Ozone: Science & Engineering* **2010**, *32* (6), 430-434. DOI: 10.1080/01919512.2010.522960.

(11) Pryor, W. A.; Cueto, R.; Jin, X.; Koppenol, W. H.; Ngu-Schwemlein, M.; Squadrito, G. L.; Uppu, P. L.; Uppu, R. M. A practical method for preparing peroxynitrite solutions of low ionic strength and free of hydrogen peroxide. *Free Radical Biology and Medicine* **1995**, *18* (1), 75-83.

(12) Wolf, C.; von Gunten, U.; Kohn, T. Kinetics of Inactivation of Waterborne Enteric Viruses by Ozone. *Environ. Sci. Technol.* **2018**, *52* (4), 2170-2177, Article. DOI: 10.1021/acs.est.7b05111.

(13) Muñoz, F.; von Sonntag, C. Determination of fast ozone reactions in aqueous solution by competition kinetics. *Journal of the Chemical Society, Perkin Transactions 2* **2000**, (4), 661-664.

(14) Flyunt, R.; Leitzke, A.; Mark, G.; Mvula, E.; Reisz, E.; Schick, R.; von Sonntag, C. Determination of• OH, O2•-, and hydroperoxide yields in ozone reactions in aqueous solution. *The Journal of Physical Chemistry B* **2003**, *107* (30), 7242-7253.

(15) Hoigné, J.; Bader, H. Characterization of water quality criteria for ozonation processes. Part II: lifetime of added ozone. **1994**.

(16) Graham-Bryce, I. J.; Nicholls, P. H.; Williams, I. H. Performance and uptake of some carbendazim-producing fungicides applied as seed treatments to spring barley, in relation to their physicochemical properties. *Pesticide Science* **1980**, *11* (1), 1-8. DOI: https://doi.org/10.1002/ps.2780110102.

(17) Sancenón, J.; De la Guardia, M. Micellar enhanced fluorimetric determination of carbendazim in natural waters. *Analytica chimica acta* **1994**, *287* (1-2), 49-57.

(18) Mazellier, P.; Leroy, E.; Legube, B. Photochemical behavior of the fungicide carbendazim in dilute aqueous solution. *Journal of photochemistry and photobiology A: Chemistry* **2002**, *153* (1-3), 221-227.

(19) Schymanski, E. L.; Jeon, J.; Gulde, R.; Fenner, K.; Ruff, M.; Singer, H. P.; Hollender, J. Identifying small molecules via high resolution mass spectrometry: communicating confidence. *Environ Sci Technol* **2014**, *48* (4), 2097-2098. DOI: 10.1021/es5002105.
